# Structural basis of a conserved, broad antigenic region surrounding the five-fold axis of foot-and-mouth disease virus revealed by swine neutralizing antibodies

**DOI:** 10.1371/journal.ppat.1014357

**Published:** 2026-07-14

**Authors:** Qiongqiong Zhao, Shanquan Wu, Fengjuan Li, Lv Lv, Shulun Huang, Jiaxin Yang, Zhanhui Zhu, Ying Sun, Pinghua Li, Yimei Cao, Huifang Bao, Pu Sun, Xingwen Bai, Zhixun Zhao, Jing Zhang, Jinyan Wu, Hong Yuan, Jijun He, Xiangmin Li, Zengjun Lu, Dongsheng Lei, Kun Li

**Affiliations:** 1 State Key Laboratory of Animal Disease Control and Prevention, College of Veterinary Medicine, Lanzhou University, Lanzhou Veterinary Research Institute, Chinese Academy of Agricultural Sciences, Lanzhou, China; 2 National Key Laboratory of Agricultural Microbiology, Hubei Hongshan Laboratory, Huazhong Agricultural University, Wuhan, China; 3 College of Veterinary Medicine, Huazhong Agricultural University, Wuhan, China; 4 School of Physical Science and Technology, Electron Microscopy Centre of Lanzhou University, Lanzhou University, Lanzhou, China; 5 Gansu Province Research Center for Basic Disciplines of Pathogen Biology, Lanzhou, China; 6 Hubei Jiangxia Laboratory, Wuhan, China; 7 Jiangsu Key Laboratory of Zoonosis, Yangzhou University, Yangzhou, China; University of Maryland at College Park: University of Maryland, UNITED STATES OF AMERICA

## Abstract

Foot-and-mouth disease virus (FMDV) exhibits extensive antigenic diversity and limited cross-protection among different serotypes, making the identification of broadly neutralizing antibodies (bnAbs) critical for the development of vaccines that confer broad and effective protection. Here, we isolated four porcine-derived bnAbs that broadly neutralized multiple representative strains from FMDV serotypes O and A. These bnAbs convergently target a conserved antigenic region, with residue 204 (K) towards the VP1 C-terminus serving as a key determinant. Notably, the bnAb pO18-10, which competes with antibodies recognizing the VP1 G-H loop, VP1 C-terminus, and VP3 epitopes, exhibits the broadest neutralization breadth, including cross-neutralization against serotype Asia1. Cryo-electron microscopy (cryo-EM) of pO18-10 in complex with FMDV serotype O (O/18074) revealed a novel cross-protomer antigenic region located around the icosahedral five-fold axis of the viral capsid, with an interaction footprint of up to 1452 Å². pO18-10 engages this epitope through interactions with the VP1 C-terminus and VP3 G-H loop of one protomer, as well as the VP1 E-F loop of an adjacent protomer, demonstrating extensive antigenic coverage. Residues 204 (K) and 210 (K) on VP1 were identified as key determinants of this cross-serotype antigenic site. Furthermore, pO18-10 sterically blocks the integrin receptor αvβ6, effectively neutralizing FMDV by preventing viral attachment. *In vivo*, pO18-10 conferred robust prophylactic and therapeutic protection against FMDV-induced mortality and clinical disease in mice. Collectively, these findings provide a promising neutralizing antibody candidate for immunodiagnostic applications and define a cross-serotype antigenic structure for universal FMDV vaccine design.

## Author summary

Foot-and-mouth disease virus (FMDV) causes highly contagious and economically devastating disease in cloven-hoofed animals. Antigenic structures capable of eliciting cross-protective neutralizing antibody responses are complex and poorly characterized. In this study, we employed four porcine-derived broadly neutralizing antibodies (bnAbs) to identify a cluster of novel epitopes conserved across FMDV serotypes O, A and Asia1. Cryo-EM analysis of the virus-antibody complex revealed the structural characteristics of the VP1 C-terminus, where the VP1 C-terminal region and VP3 G-H loop from one protomer extend to the VP1 E-F loop of an adjacent protomer, forming a spatially conserved antigenic region across FMDV serotypes O, A and Asia1. The bnAb pO18-10 covers a broad antigenic area surrounding the viral five-fold axis and neutralizes the virus effectively by preventing attachment to host cells. pO18-10 exhibited potent neutralization in vitro and demonstrated both prophylactic and therapeutic efficacy in a suckling mouse model of FMDV infection. These findings provide new structural insights into cross-serotype protective antigenic site shared by FMDV serotypes O, A, and Asia1, laying a foundation for the rational design of universal FMDV vaccine molecule.

## Introduction

Foot-and-mouth disease (FMD) is a highly contagious and economically devastating disease affecting cloven-hoofed animals [1]. Its causative agent, foot-and-mouth disease virus (FMDV), is a non-enveloped positive-sense single-stranded RNA virus belonging to the *Aphthoviruses* genus within *Picornaviridae* family [2]. Due to the lack of proofreading by its RNA-dependent RNA polymerase, FMDV exhibits significant genetic and antigenic diversity [3]. There are seven serotypes (O, A, C, SAT1, SAT2, SAT3, and Asia1) of FMDV, with numerous and constantly evolving subtypes within each serotype [4]. Although vaccination is considered an important strategy for controlling FMDV [5], effective cross-protection between serotypes is limited. The structural information available for antigenic sites that can induce cross-reactive neutralizing antibody responses remains limited, hindering the development of universal vaccines with cross-serotype protection [6].

FMDV particles exhibit a smooth, spherical structure with a diameter of approximately 25–30 nm [4]. The structural proteins VP1, VP2, and VP3 are located on the surface of the viral capsid, while VP4 is positioned internally [7]. Previous studies using murine-derived neutralizing antibodies have identified five classical antigenic sites on the surface of FMDV serotype O [8,9]. Site 1 is a linear epitope encompassing the VP1 G-H loop and C-terminus [10]. Site 2 involves the B-C and E-F loops of VP2, while site 3 maps to the VP1 B-C loop [11]. Site 4 is located on the B-B knob of VP3 [12]. Site 5 is a conformational epitope likely formed by interactions between the VP1 G-H loop and adjacent surface residues [13]. Using host-derived (bovine or porcine) neutralizing antibodies and competitive ELISA (cELISA), we previously classified six neutralizing antigenic sites on serotype O FMDV, including the VP1 G-H loop, VP1 C-terminus, site 2, site 4, site 6, and site 7 [14]. Distinct from classical sites 1–5, sites 6 and 7 exhibit unique features of broad antigenic region coverage. Site 6 involves extensive interactions with the VP1 G-H loop, VP1 C-terminus and VP3, whereas site 7 interacts with both VP2 and VP3. The results of established cELISA based on site 6 (pO18-10) showed strong correlation with virus neutralization titers in sera from vaccinated animals [15]. However, the structural basis of site 6 (pO18-10) remains unresolved and requires further investigation by cryo-electron microscopy (cryo-EM).

To date, three distinct cross-serotype antigenic sites have been identified using broadly neutralizing antibodies (bnAbs) targeting FMDV serotypes O, A and Asia1. The first site (cross-site 1) comprises linear epitopes located on the G-H loop of VP1, with the shortest binding epitope being “^145^RGDL^148^”, and the critical antigenic determinant being the RGD + 1 residue [6]. The second site (cross-site 2) is conformational, located around the three-fold axis of the capsid, interacting with residues on the βB, B-C, βC, H-I, and βI regions of VP2 and the B-C and H-I regions of VP3 across two adjacent pentamers, with VP2 D68 as a key determinant [6]. The third site is located in a shallow depression on the viral capsid, contacting the B-C, E-F, and G-H loops of VP1 and the G-H loop of VP3 from two adjacent protomers, with residue 95 of VP1 serving as a crucial antigenic determinant [16]. Additionally, other groups have reported the identification of a broadly neutralizing single-domain antibody from alpacas that can cross-react with FMDV serotypes O, A, Asia1, and C [17]. The antigenic structure of FMDV is complex, and the presence of novel cross-serotype antigenic sites remains to be fully explored.

In this work, we comprehensively characterized four bnAbs (pO18-10, pO18-17, pO18-52, and pO18-53) from the natural host, the pig. Viral neutralization tests (VNT), competitive ELISA (cELISA), enzyme-linked immunosorbent assays (ELISA), and indirect immunofluorescence assays (IFA) indicated that all four bnAbs target the same antigenic region and exhibit cross-serotype neutralizing activity. Previous studies showed that pO18-10 can compete with antibodies targeting multiple antigenic sites, including the VP1 G-H loop, the VP1 C-terminus, and VP3 [14]. To delineate the antigenic structure recognized by pO18-10, we combined cryo-EM analysis with the selection of neutralization-escape mutants, revealing cross-protomer epitopes that constitute a broadly neutralizing antigenic region centered around the five-fold axis. These findings provide a structural basis for cross-serotype antigenic sites on FMDV and offer a foundation for the rational design of universal vaccines capable of eliciting cross-reactive neutralizing antibodies.

## Results

### Identification of four broad cross-serotype neutralizing antibodies against FMDV serotypes O and A from porcine antibody repertoires

**Fig 1:**
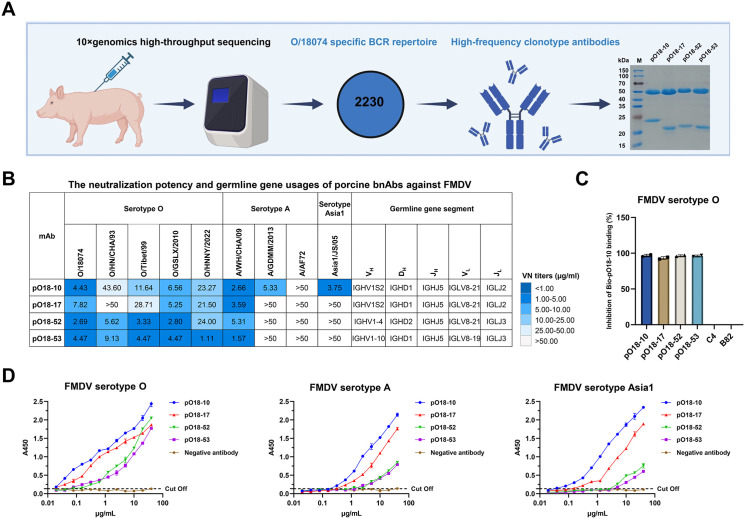
Screening and characterization of porcine-derived monoclonal antibodies against FMDV. **(A)** Workflow for screening FMDV-specific porcine monoclonal antibodies (mAbs). Peripheral blood mononuclear cells (PBMCs) were subjected to negative magnetic selection and fluorescence-activated cell sorting (FACS) to isolate antigen-specific B cells. Enriched cells were then processed for single-cell B cell receptor (BCR) and transcriptome sequencing using the 10 × Genomics platform with customized porcine BCR primers. Antibody expression plasmids were constructed, transfected into 293F cells, and purified to obtain recombinant antibodies. Created in BioRender. zhao, **Z.** (2026) https://BioRender.com/7dik6qh. **(B)** The four mAbs were evaluated for neutralization potency and breadth against representative strains of FMDV serotypes O, A and Asia1 using microneutralization assays. Neutralization titers (VN; µg/ml) are shown and color-coded. The germline gene segments (V_H_, D_H_, J_H_, V_λ_, J_λ_) corresponding to each mAb were identified and characterized. **(C)** Competitive ELISA was performed to assess epitope overlap among six mAbs (pO18-10, pO18-17, pO18-52, pO18-53, C4 and B82), using biotinylated pO18-10 as the detecting antibody. **(D)** Binding of the four porcine mAbs to antigens from O/Tibet/99, A/AF72 and Asia1/JS/05 strains was measured by indirect ELISA.

From a total of 2230 clonotypes derived from an O/18074-specific antibody repertoire, we selected 55 monoclonal antibody (mAb) clones based on their somatic hypermutation (SHM) profiles and clonal frequencies (Fig 1A). These 55 porcine-derived mAbs were successfully expressed in 293F cells and subsequently purified for functional evaluation. To assess their neutralization breadth and potency, we performed virus neutralization tests (VNTs) against a panel of representative epidemic FMDV strains circulating in China, encompassing serotypes O, A, and Asia1. This panel included strains O/HN/CHA/93 and O/18074 of the Cathay topotype, O/Tibet/99 of the ME-SA topotype, O/GSLX/2020 and O/HNNY/2022 of the SEA topotype, A/AF72 from the A22 lineage, A/WH/CHA/09 and A/GDMM/2013 from the SEA97 lineage, and Asia1/JS/05. Cross-neutralization assays revealed four mAbs (pO18-10, pO18-17, pO18-52, and pO18-53) exhibited broad neutralizing activity against multiple strains (O/18074, O/HN/CHA/93, O/Tibet/99, O/GSLX/2020, O/HNNY/2022, A/WH/CHA/09 and/or A/GDMM/2013) across both serotypes O and A, representing a cluster of cross-serotype broadly neutralizing antibodies (bnAbs) against FMDV (Fig 1B). Remarkably, pO18-10 also demonstrated effective neutralization of the Asia1/JS/05 strain, despite the fact that the pig from which the bnAbs were derived had not been vaccinated with the Asia1 antigen. Competitive ELISA analysis revealed that pO18-10 effectively competes with itself (96.7%), pO18-17 (93.1%), pO18-52 (96.4%), and pO18-53 (96.2%), indicating that these bnAbs recognize overlapping epitopes and belong to the same competition group. In contrast, pO18-10 showed no competition with the VP3-specific antibody C4 or the VP2-specific antibody B82, suggesting that it targets a distinct epitope (Fig 1C). We further assessed the reactivity of these bnAbs with FMDV antigens using indirect immunofluorescence assay (IFA) and enzyme-linked immunosorbent assay (ELISA) (Fig 1D and A in S1 Text). As expected, all four bnAbs exhibited reactivity against FMDV serotypes O (O/Tibet/99), A (A/AF72), and Asia1 (Asia1/JS/05), with pO18-10 displaying the strongest cross-serotype reactivity. Although weak fluorescence signals for pO18-17 against A/AF72 and Asia1/JS/05 were observed in IFA, specific binding at concentrations below 1 μg/ml was confirmed by ELISA.

### pO18-10 and pO18-10-like antibody (pO18-17, pO18-52, and pO18-53) escape mutants uncover a cluster of novel epitopes involving VP1 C-terminus and VP3

To explore the epitopes recognized by the four bnAbs, neutralization-escape mutants were selected against FMDV serotype O strains. Specifically, pO18-10 and pO18-17 were used to generate escape mutants of the O/18074 strain, while pO18-52 and pO18-53 were applied to the O/HN/CHA/93 strain. As shown in Table 1, neutralization-escape mutants selected by all four bnAbs exhibited similar substitution patterns, with multiple amino acid substitutions concentrated in both VP1 and VP3. Specifically, residues 99 (D) in the VP1 E-F loop, 204 (K) towards the VP1 C-terminus, and 173 (K) in the VP3 G-H loop were frequently identified in neutralization-escape mutants. All four bnAb-escaping mutants carried an amino acid substitution at residue 204 (K) towards the VP1 C-terminus, highlighting its critical role in antibody-mediated neutralization. Given that pO18-10 showed the broadest neutralizing breadth among the four bnAbs, we further characterized its key antigenic determinants across serotypes by selecting escape mutants against additional FMDV strains representing three different serotypes: O/Tibet/99, A/WH/CHA/09, and Asia1/JS/05. Compared with the O/Tibet/99 strain, the VP1 of A/WH/CHA/09 and Asia1/JS/05 is shorter by one and two amino acids, respectively. Due to these observed differences, substitutions at K204 (O/Tibet/99), K203 (A/WH/CHA/09), and K202 (Asia1/JS/05) occur at an equivalent position, suggesting that residue 204 (K) towards the VP1 C-terminus may be a critical determinant for the cross-serotype neutralization mediated by pO18-10 (Table 2). To assess the genetic stability of the mutations identified in the neutralization-escape mutants (Tables 1 and 2), nineteen mutants were subjected to three additional serial passages, followed by re-sequencing of the corresponding genomic regions. Seven of the nineteen mutants (7/19) remained genetically stable during propagation, with no changes detected at the originally identified amino acid substitution sites. In contrast, twelve mutants (12/19) exhibited alterations at these positions during passaging. Among these, ten mutants ultimately reverted to the wild-type residues during serial propagation. These reversions occurred at residues VP1–99 and VP1–143, and VP3–173 and VP3–174 in the O/18074 strain; VP1–204, VP2–68 and VP3–173 in the O/HN/CHA/93 strain; and VP3–210 in the A/WH/CHA/09 strain. In addition, a synonymous substitution (VP1-T561C) was detected in pO18-17-O/18074-mutant-C at passage 2, which reverted to the original nucleotide by passage 3 (Fig B panel A-E in S1 Text).

**Table 1:** Porcine bnAb escape mutants involved epitopes on the VP1 C-terminus.

mAb	Parent virus	Residue change(s)	Frequency of mutants^*b*^	Neutralization concn^*a*^ (*µ*g/ml)
pO18-10	O/18074	VP1 D99N(**G**AC → **A**AC), VP1 K204R(A**A**G → A**G**G)	2/8	> 400
		VP1 D99N(**G**AC → **A**AC), VP3 V174D(G**T**C → G**A**C)	1/8	> 400
		VP1 D99G(G**A**C → G**G**C), VP1 N143K(AA**C** → AA**A**), VP3 V174D(G**T**C → G**A**C)	1/8	> 400
		VP1 K204R(A**A**G → A**G**G), VP3 D173G(G**A**C → G**G**C)	2/8	> 400
		VP1 K204R(A**A**G → A**G**G), VP3 V174A(G**T**C → G**C**C)	2/8	> 400
pO18-17	O/18074	VP1 K204R(A**A**G → A**G**G)	7/8	> 400
		VP1 D99N(**G**AC → **A**AC), VP1 K204R(A**A**G → A**G**G)	1/8	> 400
pO18-52	O/HN/CHA/93	VP1 E95V(G**A**G → G**T**G), VP1 P208S(**C**CC → **T**CC)	1/8	> 400
		VP1 T174I(A**C**T→A**T**T), VP1 A207K(**GC**A → **AA**A)	1/8	> 400
		VP3 D173N(**G**AC → **A**AC)	5/8	> 400
		VP1 K204R(A**A**G → A**G**G)	1/8	> 400
pO18-53	O/HN/CHA/93	VP1 V11I(**G**TT → **A**TT)	1/7	> 400
		VP2 V107I(**G**TT → **A**TT)	1/7	> 400
		VP4 A42T(**G**CC → **A**CC)	1/7	> 400
		VP3 D173N(**G**AC → **A**AC)	2/7	> 400
		VP3 F209Y(T**T**T → T**A**T)	1/7	> 400
		VP1 K204R(A**A**G → A**G**G)	1/7	> 400

^a^ The neutralization concentration was determined as the lowest antibody concentration that protected cells from CPE.

^b^ Frequencies of the mutants are defined as the number of mutants with the amino acid substitution at the indicated residue/total number of mutants obtained.

**Table 2:** Selection of pO18-10-escaping mutants using the O/Tibet/99, A/WH/CHA/09 and Asia1/JS/05 strains.

mAb	Parent virus	Residue change(s)	Frequency of mutants^*b*^	Neutralization concn^*a*^ (*µ*g/ml)
pO18-10	O/Tibet/99	VP1 K204R(A**A**G → A**G**G)	4/8	> 400
		VP1 K204T(A**A**G → A**C**G)	2/8	> 400
		VP1 K204Q(**A**AG → **C**AG)	1/8	> 400
		VP3 A174D(G**C**T → G**A**T)	1/8	> 400
pO18-10	A/WH/CHA/09	VP1 K203N(AA**A** → AA**C**)	4/8	> 400
		VP1 K203N(AA**A** → AA**T**)	1/8	> 400
		VP1 K203P(**AA**A → **CC**A)	1/8	> 400
		VP1 A138E(G**C**G → G**A**G), VP1 K203N(AA**A** → AA**T**)	1/8	> 400
		VP1 K203Q(**A**AA → **C**AA), VP3 F210Y(T**T**T → T**A**T)	1/8	> 400
pO18-10	Asia1/JS/05	VP1 K202R(A**A**G → A**G**G), VP3 V173A(G**T**G → G**C**G)	4/8	> 400
		VP3 V173G(G**T**G → G**G**G)	2/8	> 400
		VP1 K202T(A**A**G → A**C**G)	1/8	> 400
		VP1 R199L(C**G**T → C**T**T), VP1 K202E(**A**AG → **G**AG)	1/8	> 400

^a^ The neutralization concentration was determined as the lowest antibody concentration that protected cells from CPE.

^b^ Frequencies of the mutants are defined as the number of mutants with the amino acid substitution at the indicated residue/total number of mutants obtained.

### The cryo-EM structure of FMDV-O18-pO18-10 complex uncovers a novel cross-serotype antigenic structure exhibiting broad footprints around the icosahedral five-fold axis of FMDV

**Fig 2:**
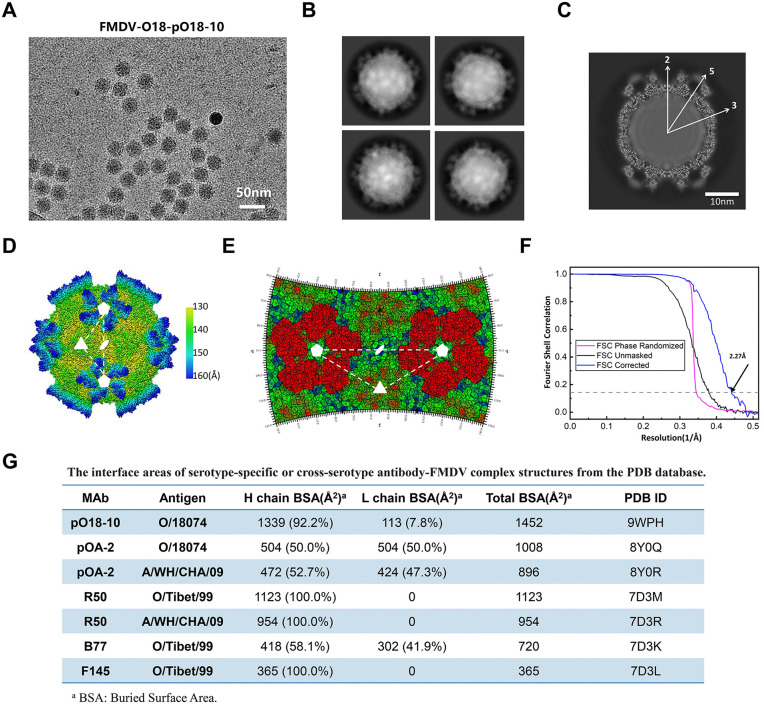
Cryo-EM structure of the FMDV-O18-pO18-10 complex. **(A)** A representative cryo-EM micrograph of the FMDV-O18-pO18-10 complex, Scale bar, 50 nm. **(B)** Selected two-dimensional class averages illustrating the distribution of bound antibodies on the viral capsid surface, Box size, 46.5 nm. **(C)** Central cross sections obtained through cryo-EM maps are shown with icosahedral 2-, 3-, and 5-fold axes. **(D)** Rendered images of the FMDV-O18-pO18-10 complex. Depth cueing with color is used to indicate the radius (< 130 Å, yellow; 140-150 Å, from green to cyan; > 160 Å, blue). The icosahedral 5- and 3-fold axes are represented by pentagons and triangles, respectively. **(E)** Footprint of pO18-10 on the FMDV surface were produced using RIVEM [46]. **(F)** Fourier shell correlation (FSC) of the final 3D reconstruction after gold-standard refinement using RELION. The resolution corresponding to an FSC of 0.143 is shown. FSC curves before (gray) and after (pink) masking, as well as the post-correction curve (purple) accounting for mask effects using phase randomization, are plotted. **(G)** Representative structures of antibody-FMDV complexes available in the PDB, including serotype-specific and cross-serotype antibodies, showing interaction interfaces with the antibody heavy and light chains and the total contact surface area.

**Fig 3:**
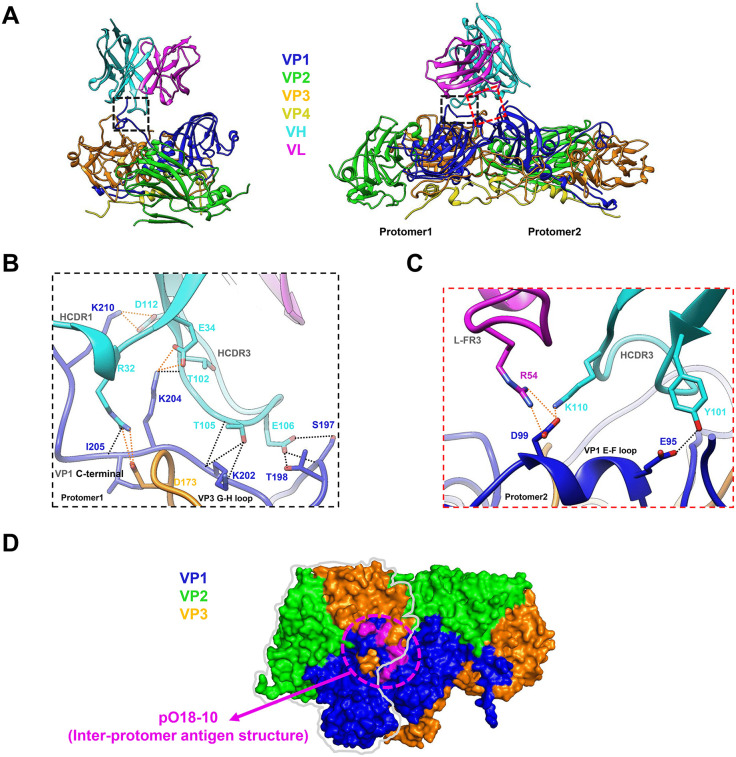
Interactions between the pO18-10 and FMDV-O18. **(A)** Cartoon representation of two adjacent protomers showing the interaction interface between pO18-10 scFv and the capsid of the O/18074 strain. The heavy chain and light chain of pO18-10 are in light blue and purple, respectively. Capsid proteins VP1, VP2, VP3 and VP4 are shown in blue, green, orange and earthy yellow, respectively. **(B-C)** Expanded views of the interaction interface highlighting the VP1 C-terminal and VP3 G-H loop within protomer 1 (B) as well as the VP1 E-F loop within protomer 2 **(C)**. Hydrogen bonds and salt bridges in the interaction interface are marked separately by black and orange dashed lines. **(D)** The epitope footprint of pO18-10 on the surface of two protomers from two adjacent pentamers of FMDV. One protomer (comprising VP1-VP4) is circled in a grey line. The epitope includes residues VP1 197, 198, 202, 204, 205 and 210, and VP3 173 from one protomer, and VP1 95 and 99 from the adjacent protomer, was marked in pink.

Next, we determined the cryo-EM structure of the pO18-10 single-chain fragment variable (scFv) in complex with the O/18074 strain of FMDV (FMDV-O18-pO18-10). Cryo-EM micrographs indicated that the scFv binds around the five-fold axis of the FMDV capsids, with a total of 60 scFv copies bound to each FMDV particle (Fig 2A to 2E). The final resolution of the cryo-EM three-dimensional (3D) reconstruction was 2.27 Å estimated by the Fourier shell correlation (FSC) 0.143 cutoff (Fig 2F). Local resolution analysis showed that the reconstruction ranges from ~2.0 Å to ~3.2 Å, including at the epitope-paratope interface (Fig C panel A in S1 Text), supporting reliable model building. Representative cryo-EM densities for the FMDV-O18-pO18-10 protomer, the VP1 C-terminus, and the antibody-virus interface are shown in Fig C panels B–D in S1 Text, with well-defined density enabling unambiguous assignment of interacting residues (Fig C panels C and E in S1 Text). Notably, compared with the previously reported FMDV-O18 structure (PDB: 8Y0Q), in which this region is poorly ordered, the VP1 C-terminus in our complex displays clear density and adopts a distinct conformation (Fig C panels D and E in S1 Text). Structural analysis of the FMDV-O18-pO18-10 complex revealed that pO18-10 engages the FMDV capsid by interacting with VP1 and VP3 from one protomer and VP1 from a neighboring protomer through both its heavy (VH) and light (VL) chains, indicating a relatively broad antigenic coverage with a total surface area of approximately 1452 Å² centered around the five-fold axis of FMDV. The antigenic footprints show more extensive coverage than any other serotype-specific or cross-serotype antibody-FMDV complex structures currently available in PDB database (Fig 2G and D in S1 Text). The interaction is predominantly mediated by the VH domain (92.2%), with a smaller contribution from VL (7.8%), as shown by PISA (Fig 3A and Table B in S1 Text). In the FMDV-O18-pO18-10 complex, the VH domain accounts for the majority of the buried surface area at the interface, forming 9 hydrogen bonds and 8 salt bridges, whereas the VL domain contributes only 3 salt bridges and plays a minor role in the interaction (Table 3).

**Table 3:** FMDV O/18074 with pO18-10 interaction residues.

FMDV Domain	FMDV Residue	Distance(Å)	pO18-10 Residue	pO18-10 CDR
**Protomer 1** **VP1 C-terminus**	I194(CD1)	3.5	S108(OG)	HCDR3
	P196(CA)	3.3	E106(OE2)	HCDR3
	**S197(N)**	**3.1**	**E106(OE2)**	**HCDR3**
	**S197(OG)**	**3.8**	**E106(OE1)**	**HCDR3**
	**T198(OG1)**	**3.9**	**E106(OE2)**	**HCDR3**
	H201(ND1)	3.2	E106(CG)	HCDR3
	**K202(N)**	**3.5**	**T105(OG1)**	**HCDR3**
	**K202(O)**	**3.0**	**T105(N)**	**HCDR3**
	**K202(O)**	**3.5**	**T105(OG1)**	**HCDR3**
	Q203(CA)	4.0	G104(CA)	HCDR3
	**K204(NZ)**	**3.0**	**T102(O)**	**HCDR3**
	*K204(NZ)*	*2.9*	*E34(OE1)*	*HCDR1*
	*K204(NZ)*	*2.8*	*E34(OE2)*	*HCDR1*
	**I205(O)**	**3.7**	**R32(NH2)**	**HCDR1**
	K210(CG)	3.6	Y101(CE1)	HCDR3
	*K210(NZ)*	*3.4*	*D112(OD1)*	*HCDR3*
	*K210(NZ)*	*3.1*	*D112(OD2)*	*HCDR3*
	Q211(O)	3.3	K3(CD)	H-FR1
	Q211(O)	3.4	F28(CB)	HCDR1
**Protomer 1** **VP3 G-H loop**	*D173(OD1)*	*3.4*	*R32(NH2)*	*HCDR1*
	*D173(OD2)*	*3.4*	*R32(NH1)*	*HCDR1*
	V174(CG2)	3.5	S54(OG)	HCDR2
	V174(CG1)	3.9	T105(CG2)	HCDR3
**Protomer 2** **VP1 B-C loop**	E47(CD)	3.4	T57(CG2)	L-FR3
**Protomer 2** **VP1 E-F loop**	**E95(OE1)**	**2.7**	**Y101(OH)**	**HCDR3**
	A96(N)	3.4	Y103(CD2)	HCDR3
	*D99(OD2)*	*3.2*	*K110(NZ)*	*HCDR3*
	*D99(OD2)*	*3.6*	*R54(NH1)*	*L-FR3*
	*D99(OD1)*	*3.1*	*R54(NH2)*	*L-FR3*
**Protomer 2** **VP1 βH**	K169(NZ)	3.3	R54(NH2)	L-FR3

The interaction residues were computed using the CCP4 [47] hydrogen bond distance cutoff of 4 Å and the salt-bridge distance cutoff of 4 Å. The bold font refers to a hydrogen bond. The italic font refers to salt bridge.

On one protomer, residues in VP1 C-terminus (_O18-VP1_I194, _O18-VP1_P196, _O18-VP1_S197, _O18-VP1_T198, _O18-VP1_H201, _O18-VP1_K202, _O18-VP1_Q203, _O18-VP1_K204, _O18-VP1_I205, _O18-VP1_K210 and _O18-VP1_Q211) and VP3 G-H loop (_O18-VP3_D173 and _O18-VP3_V174) interact with residues across the VH of pO18-10. Notably, extensive contacts are mediated by HCDR3 (_VH_Y101, _VH_T102, _VH_G104, _VH_T105, _VH_E106, _VH_S108 and _VH_D112), along with contributions from HCDR1 (_VH_F28, _VH_R32 and _VH_E34), H-FR1 (_VH_K3), and HCDR2 (_VH_S54) (Fig 3B). Meanwhile, residues in the VP1 B-C loop (_O18-VP1_E47), VP1 E-F loop (_O18-VP1_E95, _O18-VP1_A96 and _O18-VP1_D99) and VP1 βH (_O18-VP1_K169) from another protomer interact with residues in L-FR3 (_VL_R54, _VL_T57), as well as HCDR3 (_VH_Y101, _VH_Y103 and _VH_K110) (Fig 3C). The side chains of _O18-VP1_S197 and _O18-VP1_T198 form hydrogen bond contacts with the _VH_E106 side chain. Additionally, the residues _O18-VP1_K202, _O18-VP1_K204, _O18-VP1_I205 and _O18-VP1_E95 also form hydrogen bond contacts with the _VH_T105, _VH_T102, _VH_R32 and _VH_Y101, respectively. Meanwhile, the side chains of _O18-VP1_K204, _O18-VP1_K210, _O18-VP3_D173 and _O18-VP1_D99 form salt bridges with the side chains of _VH_E34, _VH_D112, _VH_R32, _VH_K110 and _VL_R54, respectively. Obviously, residue 204 (K) towards the VP1 C- terminus involved both hydrogen bond and salt bridge contacts, suggesting a key antigenic determinant in the interface. These interactions enable pO18-10 to simultaneously engage the C-terminus and E-F loop of VP1 located on different protomers. The footprint of key residues involved in the epitope of pO18-10 was mapped on the surface of FMDV across two neighboring protomers (Fig 3D).

### Key determinants of pO18-10 and pO18-10-like antibodies reveal a novel cross-serotype antigenic site

**Fig 4:**
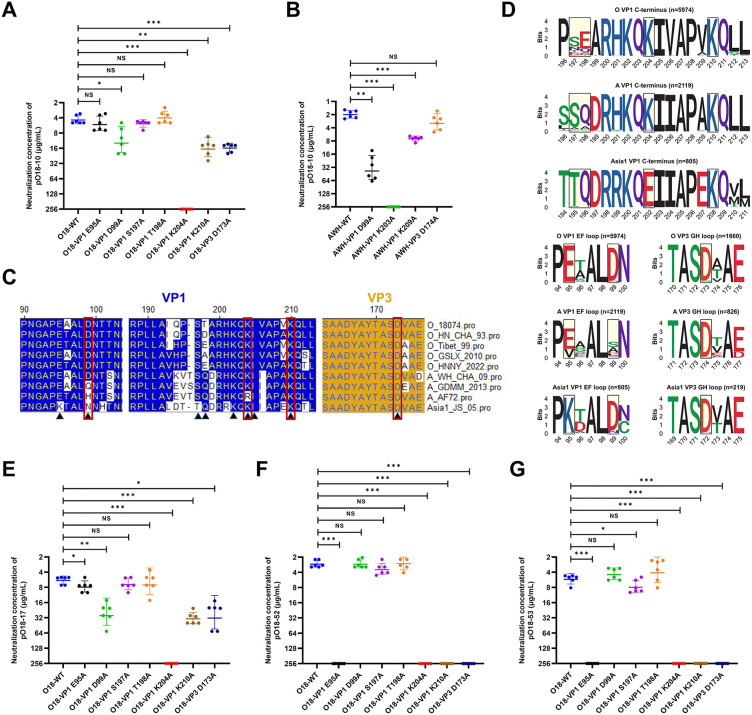
Functional analysis of pO18-10 and related bnAbs against wild-type and mutant FMDV strains. **(A)** Neutralization efﬁcacy of pO18-10 against the wild-type (O/18074) and mutant viruses (VP1 E95A, VP1 D99A, VP1 S197A, VP1 T198A, VP1 K204A, VP1 K210A, and VP3 D173A) was assessed by microneutralization assay. **(B)** Neutralization efﬁcacy of pO18-10 against the wild-type (A/WH/CHA/09) and mutant (VP1 D99A, VP1 K203A, VP1 K209A, and VP3 D174A) viruses was assessed by microneutralization assay. **(C)** Sequence alignment of VP1 and VP3 regions from representative FMDV strains of serotypes O, A and Asia 1. **(D)** Full-length amino acid sequences of available FMDV serotypes O, A, and Asia1 were downloaded from the National Center for Biotechnology Information (NCBI) database as of June 30, 2025. Sequence logos were generated for the VP1 (serotype O, *n* = 5,974; A, *n* = 2,119; Asia1, *n* = 805) and the VP3 (O, *n* = 1,660; A, *n* = 826; Asia1, *n* = 219). **(E to G)** Neutralization efﬁcacy of pO18-17 **(E)**, pO18-52 **(F)**, and pO18-53 **(G)** against the wild-type (O/18074) and the same panel of site-directed mutants as in **A**. The neutralization concentration represented the lowest antibody concentration required to fully prevent CPE. The experiments were independently conducted in triplicate. The neutralization differences between wild-type and its mutants were determined using unpaired T test (Holm-Sidak method, with α = 0.05) in GraphPad Prism 9.5.1. * Indicates an extremely significant difference to wild-type at P < 0.05. ** Indicates an extremely significant difference to wild-type at P < 0.01. *** Indicates an extremely significant difference to wild-type at P < 0.001. NS indicates no significant difference.

To further validate the crucial determinants of FMDV serotype O for pO18-10, we substituted alanine for specific capsid residues involved in forming hydrogen bonds or salt bridges at the interface of the virus-antibody complex. A total of seven single-substitution mutants were successfully rescued (Fig F panel A in S1 Text) and assessed for neutralization by pO18-10. As shown in Fig 4A, amino acid substitutions at VP1 positions 99, 204, and 210, as well as VP3 position 173, markedly reduced neutralization potency, indicating that these four residues constitute key antigenic determinants recognized by pO18-10 in serotype O. Next, to examine whether these determinants are conserved across serotypes, alanine substitutions at positions _VP1_99, _VP1_203, _VP1_209 and _VP3_174 on serotype A (because of residues deletion at positions 140, 141 and the addition at position 197 on VP1 of A/WH/CHA/09 strain in alignment with serotype O), corresponding to those positions _VP1_99, _VP1_204, _VP1_210 and _VP3_173 on serotype O, were introduced into the A/WH/CHA/09 strain (serotype A). Four single-residue mutants were successfully rescued (Fig F panel B in S1 Text), and used for cross-neutralization assay with pO18-10. As shown in Fig 4B, amino acid substitutions at VP1 positions 99, 203, and 209 in serotype A (A/WH/CHA/09) significantly reduced pO18-10 neutralization. In particular, both the _O18-VP1_K204A and _AWH-VP1_K203A mutants completely escaped antibody-mediated neutralization (VN titer > 250 μg/ml) (Fig 4A and 4B), highlighting the functional importance of this residue in both serotypes O and A. However, sequence alignment revealed that the critical VP1 residue 203/204 (K) is not strictly conserved across all representative laboratory strains of different FMDV serotypes (Fig 4C). Alignment of viral capsid protein sequences available in GenBank (NCBI, as of June 30^th^, 2025) revealed that _VP1_204 (K) is conserved in both serotypes O and A, whereas _VP1_210 (K) and _VP3_173 (D) are highly conserved across serotypes O, A, and Asia1. Notably, VP1 is predominantly 213 amino acids in length in serotypes O and A, and 211 amino acids in Asia1, while VP3 lengths are 220, 221 and 219 amino acids for serotypes O, A and Asia1, respectively. However, residues at positions _VP1_95 and _VP1_99 appeared diverse among these serotypes. For Asia1, VP1–99 shows a bias toward Asp (D), VP1–210 is conserved as Lys (K), and VP3–173, although variable, also tends toward Asp (D), consistent with patterns observed in types O and A. Thus, we concluded that the conserved residues _VP1_204 (K) and _VP1_210 (K), located towards the VP1 C-terminus, contribute to cross-serotype neutralization and represent crucial determinants of the cross-serotype antigenic site (Fig 4D).

To determine whether the interface residues on serotype O also affect the neutralizing activity of other pO18-10-like antibodies (pO18-17, pO18-52 and pO18-53), cross-neutralization assays were performed against each variant. For pO18-17, amino acid substitutions at VP1 positions 95, 99, 204, and 210, as well as VP3 position 173, significantly reduced virus neutralization titer, with the VP1–204 substitution resulting in complete escape from neutralization (VN titer >250 μg/ml) (Fig 4E). For pO18-52, amino acid substitutions at VP1 positions 95, 204, and 210 and VP3 position 173 each conferred complete escape from neutralization (VN titer >250 μg/ml) (Fig 4F). For pO18-53, amino acid substitutions at VP1 positions 95, 197, 204, and 210, as well as VP3 position 173, markedly impaired neutralization, with substitutions at VP1 positions 95, 204, 210 and VP3 position 173 leading to complete escape (VN titer >250 μg/ml) (Fig 4G). Collectively, these findings demonstrate that the pO18-10 and pO18-10-like antibodies convergently target the same antigenic site, while the key determinants differentially interact with diverse antibodies within this broadly neutralizing antibody lineage.

### The porcine-derived bnAb pO18-10 neutralizes FMDV by blocking virus attachment to cells

**Fig 5:**
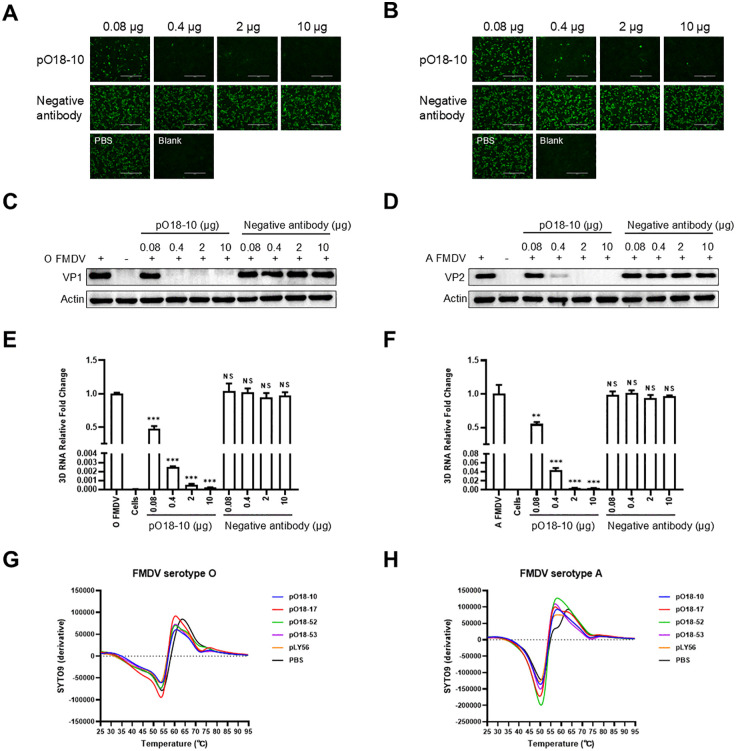
Identification of cross-serotype neutralization mechanisms of pO18-10 against FMDV serotypes O and A. **(A-F)** The inhibition effect of pO18-10 on viral attachment to BHK-21 cells. Different amounts of pO18-10 were respectively pre-incubated with FMDV serotype O (O/18074 strain) **(A, C, E)** or serotype A (A/WH/CHA/09 strain) **(B, D, F).** The viruses were quantified through detecting VP1 protein by IFA **(A, B)** and Western blotting **(C, D)** and 3D gene by qRT-PCR **(E, F)**. The experiments were performed in triplicate. The data differences between conditions with virus only and different bnAb treatments were assessed using unpaired T-test (Holm-Sidak method, α = 0.05) in GraphPad Prism 9.5.1.*** Indicates an extremely significant difference at P < 0.001. ** Indicates a very significant difference at P < 0.01. NS indicates no significant difference. **(G, H)** Thermostability assay of FMDV serotypes O and A in the presence of four porcine-derived bnAbs. The Y-axis indicates the derivate of raw fluorescence representing the level of viral RNA release. PBS was used as a blank control.

Cell attachment is the initial step in the viral entry process, and blocking this interaction has been identified as a critical mechanism for virus neutralization by antibodies [18,19]. To further understand the mechanisms underlying FMDV neutralization and protection, we examined the inhibitory effects of pO18-10 on FMDV binding to BHK-21 cells at two distinct stages: pre- and post-attachment. For pre-attachment stage, the serotype O FMDV was first incubated with pO18-10 at different doses, then the non-neutralized virus was allowed to attach BHK-21 cells. As shown in Fig 5, we observed a significant reduction in immunofluorescence signals (Fig 5A), structural protein expression (Fig 5C), and viral RNA levels (Fig 5E) in BHK-21 cells following incubation with pO18-10, compared to the control group treated with a negative porcine mAb (PLY57). The consistent results were also observed for antibodies incubation with serotype A FMDV (Fig 5B, 5D and 5F). Moreover, structural superposition between FMDV-integrin (generally αvβ6) and FMDV-antibody complexes revealed notable steric clashes between pO18-10 and the integrin receptor, suggesting that FMDV neutralization by pO18-10 is likely facilitated by blocking the virus-receptor interaction through steric hindrance (Fig G in S1 Text). These results showed that pO18-10 is capable of blocking virus attachment to cells. For post-attachment assay, pO18-10 was incubated with the BHK-21 cells pre-absorption with FMDV and tested whether affecting viral entry. Specifically, as shown in Fig H in S1 Text, pO18-10 did not obviously affect viral entry into the cells, with only a partial reduction in viral RNA observed at high antibody concentrations. This phenomenon may be influenced by the detection method used, possibly owing to the greater sensitivity of quantitative RT-qPCR.

Next, we determined whether pO18-10 neutralizes FMDV via an alternative mechanism, such as destabilization of virion structure and initiation of viral RNA release or more violent mechanism by dissociation of viral particle. We investigated their effect on the stability of FMDV upon binding with all these four antibodies by PaSTRy assays using SYTO9 dye to detect exposure of viral RNA. Similar to the negative porcine mAb (PLY57) and the PBS control, binding of pO18-10 and pO18-10-like antibodies did not alter the stability of FMDV particles, with consistent results observed for both FMDV serotypes O and A (Fig 5G and 5H). These findings indicate that pO18-10 mediates cross-serotype neutralization of FMDV primarily by blocking viral attachment to host cells.

### Prophylactic and therapeutic efficacies of the porcine-derived bnAb pO18-10

**Fig 6:**
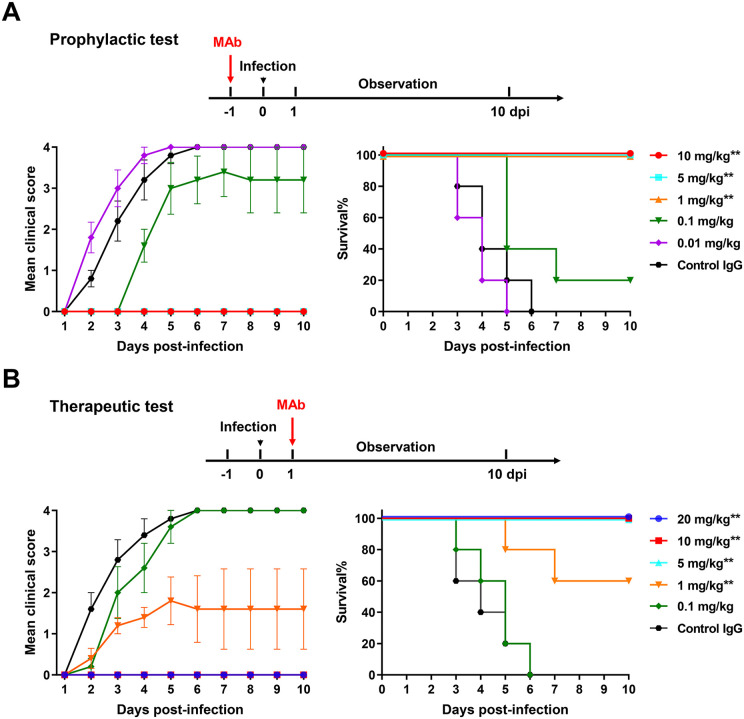
Prophylactic and therapeutic efficacy of pO18-10 against FMDV infection in suckling KM mice. **(A)** Prophylactic efficacy of pO18-10 against lethal challenge with O/18074. Clinical scores (left) and survival (right) curves of suckling KM mice (n = 5 per group) treated with 10, 5, 1, 0.1, or 0.01 mg/kg pO18-10, or pO18-36 (10 mg/kg) 24 h before lethal challenge are shown. **(B)** Therapeutic efficacy of pO18-10 against lethal challenge with O/18074. Clinical scores (left) and survival (right) curves of suckling KM mice (n = 5 per group) treated with 20, 10, 5, 1, or 0.1 mg/kg pO18-10, or pO18-36 (20 mg/kg) 24 h after lethal challenge are shown. Upper panel: study outline. Lower panel: clinical score and survival. Clinical scores were graded as follows: 0, healthy; 1, reduced mobility; 2, limb weakness; 3, limb paralysis; 4, death. For survival data, the Log-rank (Mantel-Cox) test was used to evaluate significance. *** Indicates an extremely significant difference at P < 0.001. ** Indicates a very significant difference at P < 0.01. * Indicates a significant difference at P < 0.05. All error bars represent SEM.

The protective effects of bnAb pO18-10 was assessed in a Kunming (KM) suckling mice model of FMDV infection, which was based on the mouse-adapted strain FMDV/O/18074. For the prophylaxis study, groups of 2-day-old KM suckling mice were subcutaneously injected in the dorsal neck region with 10, 5, 1, 0.1 or 0.01 mg/kg of pO18-10, or with 10 mg/kg of control IgG (pO18-36). Twenty-four hours later, the mice were challenged with FMDV/O/18074. Survival and clinical scores were recorded daily and were depicted in Fig 6A. Mice treated with the control IgG started to exhibit characteristic clinical symptoms, including reduced mobility, limb weakness and paralysis, at 2 days post-infection (dpi), and all of the mice in the two groups eventually died. In contrast, all of the mice treated with pO18-10 at doses of 1 mg/kg and above were completely protected from death, and all of these mice showed no clinical signs of illness.

To evaluate the therapeutic efficacy of bnAb pO18-10, groups of 2-day-old KM suckling mice were infected with FMDV/O/18074 and 1 day later treated with 20, 10, 5, 1 or 0.1 mg/kg of pO18-10, or 20 mg/kg of control IgG (pO18-36). Survival and clinical scores were monitored daily (Fig 6B). Mice receiving the control IgG began to exhibit clear clinical symptoms at 2 dpi and showed significant mortality rate (100%) by 6 dpi. In contrast, mice administered pO18-10 at doses of 5 mg/kg and above showed 100% survival rates at 10 dpi, and none of the mice displayed any signs of infection during the course of the study. These results demonstrate that pO18-10 confers potent prophylactic and therapeutic protection against FMDV infection in vivo.

## Discussion

To date, four cross-serotype antigenic structures (cross-site 1–4) have been determined on FMDV using a panel of 15 broadly neutralizing antibodies (bnAbs) from natural hosts (Fig E in S1 Text). Specifically, bnAbs targeting cross-site 1 and cross-site 2 were derived from 216 clonotype antibodies shared between the porcine O/18074- and A/AF72-specific B cell libraries. The cross-site 3-related bnAb R50 was isolated from a natural bovine host using single B cell isolation and bnAbs targeting cross-site 4 were identified in this study from the porcine O/18074-specific B cell library. Considering the number of antibodies targeting each cross-site, the newly resolved cross-site 4 (recognized by pO18-10 and pO18-10-like antibodies) may represent the second most immunodominant cross-site, after cross-site 1, which is recognized by a group of 9 bnAbs. Notably, the footprints of pO18-10 showed the widest antigenic coverage on the surface of FMDV. This unique binding mode makes pO18-10 a promising candidate for establishing a competitive ELISA to evaluate protective serum responses following FMDV vaccination. A previous study reported that the bovine bnAb R50 recognizes an epitope located in a shallow depression between the two-fold and five-fold axes, contacting VP1 and VP3 from adjacent protomers [16]. The neutralizing site recognized by R50 includes the B-C loop (residues 50 and 52), E-F loop (residues 94–95), and G-H loop (residues 157 and 159–160) of VP1, along with the G-H loop of VP3 (residues 173 and 177). While pO18-10 and R50 share a partially overlapping epitope, the key determinant for cross-serotype neutralization by pO18-10 is residue 204 of VP1, whereas that for R50 is residue 95 of VP1. It is noteworthy that R50 exerts its neutralizing activity by promoting the dissociation of FMDV, whereas pO18-10 inhibits viral attachment to host cells by competing with the αvβ6 integrin receptor.

Although previous analyses focused on common clonotypes [6], the incidental identification of bnAbs from type O-specific clonotypes suggests that restricting analyses to common clonotypes may underestimate the breadth of neutralizing responses. The cross-serotype activity of pO18-10 further indicates that rare, serotype-restricted clones can recognize structurally conserved epitopes. Together, these findings highlight a potential limitation of current screening strategies and suggest that integrating both common and serotype-specific clonotypes may be necessary to fully define the landscape of protective antibody responses.

The protruding G-H loop of VP1 has long been recognized as an immunodominant region, with numerous serotype- or strain-specific monoclonal antibodies (mAbs) targeting this loop in serotypes O, A, C, and Asia1 [20–23]. However, the precise conformation of the VP1 G-H loop on the intact FMDV virion remains unresolved. Previous competitive ELISA assays demonstrated that pO18-10 broadly competes with antibodies targeting antigenic sites involving the VP1 G-H loop, VP1 C-terminus, and VP3, and shows immunodominance in sera from different host species, including cattle, sheep, and pigs [14]. Previously, we developed a competitive ELISA assay to assess vaccine-induced immunity against serotype A using the serotype A-specific bnAb W145, which targets the VP1 G-H loop and reliably measures antibody responses while confirming that W145 recognizes an immunodominant site [24]. Similarly, based on the immunodominance of pO18-10, we established a competitive ELISA to quantify immune responses following serotype O vaccination [14]. The site 4-type mAb C4 recognizes discontinuous epitopes across multiple regions of VP3, including the B-B knob (E58, D60, and V61), βB (T65), the B-C loop (T68 and R72), the E-F loop (E134 and K135), and the H-I loop (H191, D195, and D197). It also contacts VP2 from the adjacent protomer, including the B-C loop (T71 and S72) and H-I loop (Q196 and K198) of [25]. Although the epitopes of pO18-10 and C4 do not overlap, competitive ELISA assays revealed strong competition between the two mAbs, likely due to steric hindrance arising from the broad antigenic coverage of pO18-10.

Notably, pO18-10 failed to neutralize the A/AF72 strain, and sequence alignment revealed that the VP1 residues at positions 99 and 204, which serve as key contact sites for pO18-10, are not conserved in this strain (Fig 4C). To further assess their role, residues VP1 H99 and R204 in A/AF72 were substituted to match those in serotype O, and the recombinant viruses were successfully rescued. However, neutralization assays showed that these single substitutions alone were insufficient to restore pO18-10-mediated neutralization (Fig I in S1 Text), suggesting that pO18-10’s inability to neutralize A/AF72 may involve a combination of multiple residue differences or a more complex conformational epitope.

Passive immunotherapy using mAbs has shown significant efficacy against FMD and other viral diseases [17,26,27]. For instance, a single 20 μg dose of Japanese encephalitis virus (JEV)-specific mAbs (2F2 and 2H4) was sufficient to completely clear viral infection from murine brain tissue and provided 100% survival in challenged mice [27]. Given the highly contagious nature of FMD, emergency administration of immune sera or mAbs may offer rapid prophylactic protection during outbreaks [28,29]. Previous studies have shown that a cocktail of VHH single-domain mAbs, such as M8 and M23, can confer partial protection in a guinea pig challenge model [26]. Subsequent research demonstrated that treatment with M8 and M170 significantly reduces infection rates and delays the onset of clinical symptoms, although complete protection was not achieved [17]. In the present study, we demonstrated that pO18-10 conferred complete protection in KM suckling mice at doses ≥1 mg/kg for prophylactic administration and ≥5 mg/kg for therapeutic intervention. These results confirm the potent in vivo protective capacity of pO18-10 and support its potential as a therapeutic candidate for FMDV. While mAbs are unlikely to replace vaccination, they could provide rapid, short-term protection in outbreaks, serve as a bridge until vaccine-induced immunity develops, or protect high-value animals, highlighting their potential as a complementary tool in FMD control.

Evidence suggests that prophylactic peptide vaccines targeting the VP1 G-H loop in combination with T-cell peptides may confer effective experimental protection in swine [30,31]. Furthermore, while linear epitopes of the G-H loop provide a foundation, the inclusion of complex conformational epitopes is likely critical for eliciting high-titer neutralizing antibodies. Given that single-epitope strategies often prove insufficient in livestock, future vaccine design should focus on multi-component platforms that combine T-cell stimulation with structurally authentic B-cell epitopes to ensure a more comprehensive and durable immune response. It is well established that FMDV can readily adapt to use heparan sulfate (HS) as a receptor during passage in BHK cells, a process frequently associated with amino acid substitutions at residues 56 (H56R) or 58 (K56E) of VP3 [32,33]. In our study, however, sequencing of the neutralization-resistant mutants showed that residues 56 and 58 in VP3 remained unchanged. One possible explanation is that the parental virus used in this study had already adapted to BHK cells and acquired the ability to utilize HS as a receptor prior to the selection experiment. Furthermore, structural superposition between FMDV-integrin and FMDV-antibody complexes revealed notable obvious clashes between pO18-10 and the integrin receptor, suggesting that the neutralization was achieved primarily through blocking the interaction with the integrin receptor.

In summary, our study defines the structural basis of the FMDV-specific bnAb pO18-10, which exhibits broad antigenic coverage by targeting the five-fold axis of the viral capsid. We further elucidate its mechanism of action. pO18-10 confers potent in vivo protection and represents a promising candidate for therapeutic development. These findings provide important insights for the rational design of next-generation FMDV vaccines and antibody-based interventions.

## Materials and methods

**In vitro expression of porcine mAbs.** Porcine-derived mAbs (pO18-10, pO18-17, pO18-52 and pO18-53) were produced from peripheral blood mononuclear cells (PBMCs) of pigs sequentially immunized with two serotypes of FMDV O and A using single B-cell antibody library technology, as described in our previous report [6].The selected pair of VH and VL genes were codon-optimized and respectively cloned into porcine heavy chain (CH-pcDNA3.4) and light chain (Cκ-pcDNA3.4 or Cλ-pcDNA3.4) expression vectors to obtain the whole antibody-expressing plasmids VH-CH-pcDNA3.4 and VL-Cκ-pcDNA3.4/or VL-Cλ-pcDNA3.4, as described in our previous report [34]. The recombinant formatted single-chain fragment variable (scFv) was designed by joining VH and VL fragments using a flexible linker (GGGGSGGGGSGGGGS) and a His tag (HHHHHH) added at the C-terminus. The final codon-optimized (*Cricetulus griseus*) scFv gene was cloned into pcDNA3.4 expression vector. The scFv or the antibody expressing plasmids with a light-to-heavy chain ratio of 3:2 was transfected into the suspended 293F cells (Invitrogen, USA) and continued cultivation for 5 days. The expressed mAbs in supernatants were initially purified through Ni-chelating affinity chromatography and further purified by size exclusion chromatography using a Superdex 200 increase 10/300 column in an AKTA plus protein purification system (GE Life Sciences). The concentration of expressed mAbs was determined by measuring the absorbance values at a wavelength of 280 nm (A280).

**Virus neutralization test.** The porcine mAbs were titrated for viral neutralizing activity against multiple FMDV serotypes O, A and Asia1 strains, including O/18074, O/HN/CHA/93 strains (Cathay topotype), O/Tibet/99 strain (ME-SA topotype), O/GSLX/2010 and O/HNNY/2022 strains (SEA topotype), A/AF72 strain (A22 lineage), A/WH/CHA/09 and A/GDMM/2013 strains (SEA97 lineage) in Asia topotype and Asia1/JS/05 strain, as well as the rescued virus by using a microneutralization assay as previously described [35]. Briefly, antibody samples were 2-fold serially diluted in 96-well cell culture plates in a total volume of 50 μl, and then 100 TCID_50_ of FMDV in 50 μl of culture media was added to each well. After incubation for 1 h at 37˚C, ~ 5 × 10^4^ BHK-21 cells in 100 μl medium were added to each well. Normal cell wells, 0.1, 1, 10 and 100 TCID_50_ virus control wells in duplicate were used in each plate. The plates were incubated at 37˚C under 5% CO_2_ conditions for 48 h before observing cytopathic effect (CPE). The experimental results were acceptable when complete CPE and no CPE appeared separately in 0.1 TCID_50_ and 100 TCID_50_ virus control wells. The endpoint titers were calculated as the reciprocal of the last antibody dilution to neutralize 100 TCID_50_ FMDV in 50% of the wells. Neutralizing activity is expressed as the VN titer, which was calculated as the initial antibody concentration divided by the endpoint titer.

**Enzyme-Linked Immunosorbent Assay (ELISA).** A competitive ELISA consistent with the previous experiments was used to assess whether pO18-10, pO18-17, pO18-52, pO18-53, C4 and B82 belong to the same class of antibodies [14]. pO18-10 was biotinylated (Bio-pO18-10) using the EZ-Link Sulfo-NHS-LC biotin reagent (Thermo Fisher Scientific, USA) following the manufacturer’s instructions. Initially, 0.5 µg/ml E32 was coated in 96-well ELISA plates overnight at 4°C. After three washes with PBST, the 146S antigens at 1 µg/ml were added into plates and captured for 2 h at room temperature. After three washes with PBST, the plates were blocked with 1% gelatin in PBS at 37°C for 2 h. After three washes, competitor mAbs were initially diluted to 50 µg/ml and then subjected to eight rounds of 2-fold dilutions to compete with Bio-pO18-10, followed by incubation at 37°C for 1 h. After five washes with PBST, HRP-conjugated streptavidin (diluted 1:30,000) was added, and the plates were incubated at 37°C for 15 min. Following another five washes with PBST, 100 μl of TMB substrate was added to each well, and the plates were incubated at 37°C for 15 min. The process was stopped by adding equal volumes of 2 M H_2_SO_4_. The optical density at at 450 nm (A450) was measured on a microplate reader. The ELISA plate included four wells of PBS control (100% nonreactivity) and four wells of Bio-pO18-10 control. The highest and lowest absorbance values at A450 were excluded, and the average of the remaining two wells was calculated. The percentage inhibition of competitor mAb to Bio-pO18-10 binding was calculated using the formula: Inhibition (%) = (A450 of Biotin-mAb control- A450 of competitor mAb) / (A450 of Biotin-mAb control- A450 of PBS control). The inhibition rates of all competing mAbs at the same optimal concentration are plotted, and only results with an inhibition rate greater than 0 are shown.

Indirect ELISA was used to assess the reactivity of the mAbs (pO18-10, pO18-17, pO18-52 and pO18-53) with the FMDV 146S antigen. In the indirect ELISA experiments, 200 ng/well viral antigen was coated in 96-well plates overnight at 4°C. The plates were then washed three times with PBST (PBS buffer plus 0.05% Tween 20) and blocked with 1% gelatin in PBS at 37°C for 2 h. After three washes, the mAbs at a concentration of 0–40 μg/ml was added and incubated at 37°C for 1 h. The plates were washed three times with PBST, and then HRP-conjugated goat anti-porcine IgG (GenScript, China) at a dilution of 1:5,000 was added to the wells. The plates were incubated at 37°C for 30 min and washed three times with PBST. The color was developed by adding 100 μl of tetramethylbenzidine (TMB) substrate (Pierce, Life Technology) incubated at 37°C for 15 min. The process was stopped by adding equal volumes of 2 M H_2_SO_4_. The optical density at 450 nm (A450) was measured on a microplate reader (Bio-Rad).

**Selection of neutralization-escape mutants using porcine-derived bnAbs.** Neutralization escape mutants were generated by consecutive passages of FMDV in BHK-21 cells under the pressure of bnAbs according to a previous report, with minor modifications [10]. The representative FMDV strains (O/18074 and O/HN/CHA/93) were employed to select mutants against these mAbs. Briefly, 10-fold serial dilutions of FMDV in 50 μl were incubated with 50 μl of various concentrations of bnAbs (20 μg/ml to 50 μg/ml) in 96-well microplates. The mixtures were used to infect 100 μl of BHK-21 cells (10^6^ cells/ml), which were incubated at 37°C for 48 h to allow virus propagation to occur. First-passage viruses were harvested from wells seeded with the highest dilution of virus that produced an approximately 80–100% cytopathic effect (CPE). Subsequent rounds of pressure selection were performed in 24-well plates, in which the passaged virus (200 μl) was incubated with an equal volume of a 2-fold concentration of antibodies in each well containing 400 μl of BHK-21 cells. The harvested virus was subjected to several rounds of selection until it completely escaped neutralization after addition of bnAbs at concentrations of at least 400 μg/ml. The P1 region of the obtained neutralization escape mutants was amplified by one-step reverse transcription-PCR (RT-PCR), as described previously [36], using the primer pair Pan204+ (ACCTCCAACGGGTGGTACGC)/NK61 (GACATGTCCTCTTGCATCTG) and subsequently verified by sequencing. Mutated amino acids were determined by aligning the entire mutant P1 region to the sequence of its initial parent virus.

**Preparation of a single-chain variable fragment of bnAb pO18-10.** The recombinant formatted single-chain fragment variable (scFv) was designed by joining VH and VL genes using a flexible linker (GGGGSGGGGSGGGGS) and a His tag (HHHHHH) added at the C-terminus. The final optimized scFv gene was cloned into pcDNA3.4 expression vector. For scFv expression, the plasmid was transfected into the suspended 293F cells (Invitrogen, USA) according to the manufacturer’s instructions. The expressed scFv was initially purified with a HisTrap Excel column. The eluate obtained was concentrated using a 10-kDa ultrafiltration tube and further purified by size exclusion chromatography using a Superdex-200 increase 10/300 column and an AKTA plus protein purification system (GE Life Sciences, USA). The purity and size of the scFv antibody were assessed by SDS-PAGE. The concentration of the final scFv obtained was determined by measuring the absorbance values at a wavelength of 280 nm (A280).

**Virus production and purification.** FMDV (O/18074) was propagated in the baby hamster kidney cells (BHK-21) that have been cultured adherently in MEM medium using a 5-layer cell factory system (NEST, China) at 37˚C with 5% CO_2_ for 48 h. After incubation with FMDV under the same conditions for 12 h, the cell supernatant was collected following three freeze-thaw cycles. Viruses in supernatant were inactivated by treating with 1.2% Binary ethylenimine (BEI) at 30˚C for 28 h, followed by the addition of a stop solution containing 4% sodium thiosulfate to neutralize the effect of BEI. The deactivated viruses were checked in BHK-21 cells for three successive passages, and no CPE was observed within each passage for a duration of 48 h, indicating complete viral inactivation. For purification of 146S antigen, approximately 1 liter of the inactivated viruses were precipitated by incubating at 4˚C overnight in 8% (w/v) PEG 6,000. The precipitated virus antigens were harvested by centrifugation at 3,500 g for 1 h at 4˚C, followed by resuspension in 50 ml PBS (137 mM NaCl, 2.7 mM KCl, 50 mM Na_2_HPO_4_ and 10 mM KH_2_PO_4_, pH = 7.6). Subsequently, viral antigens were pelleted through a cushion of 30% (wt/vol) sucrose in PBS by centrifugation at 35,000 × g for 4 h. The sucrose was removed from the pellet, and 500 μl of PBS was added to cover the pellet. The supernatant was further purified over a 20–60% sucrose gradient and fractionated by centrifugation at 35,000 g for 4 h at 4˚C.The fractions were analyzed by negative-stain electron microscopy, and the fraction corresponding to 146S antigen was transferred to a 100-kDa-molecular-weight-cutoff (MWCO) centrifugal filter for buffer exchange with PBS to remove the sucrose. The concentrations of the 146S antigens were quantified by a spectrophotometer at 260 nm (where an optical density of 7.6 = 1 mg/ml), and immediately used for subsequent experiments.

**Cryo-EM sample preparation and data collection.** FMDV O/18074 146S was mixed with scFv at a molar ratio of 1:180 and incubated for 10 minutes at 4 °C. A 4 μl sample of this complex was applied to a glow-discharged 400 mesh grid (Quantifoil Au 1.2/1.3). Grids were blotted for 5 s at 4 °C and 100% humidity before rapid plunge-frozen in liquid ethane using a Vitrobot mark IV (Thermo Fisher, USA). Cryo-EM data acquisition was carried out on a 300 kV Titan Krios G3i (Thermo Fisher, USA) microscope equipped with a direct electron detector (K3 Bioquantum, Gatan) at Lanzhou University. Image stacks were recorded as 40-frame movies over 3.0 seconds exposures, with a defocus range of -2.0 to -1.0 μm, at a calibrated magnification of ×105,000X, resulting in a pixel size of 0.83 Å.

**Image processing and three-dimensional reconstruction.** The raw movies stacks were processed in cryoSPARC [37]. Frame alignment and dose weighting were performed to generate motion-corrected micrographs. The contrast transfer function (CTF) estimation was performed using CTFFIND4 [38]. Approximately 400 particles were manually picked and subjected to 2D classification to generate templates. Particles were then automatically picked up based on the templates and subjected to multiple rounds of 2D classification. Subsequent ab-initio reconstruction, homogeneous refinement and non-uniform refinement were all carried out in cryoSPARC. The resolution of the final map was assessed using the standard FSC = 0.143 criterion. The data collection and refinement statistics are summarized in Table A in S1 Text.

**Model building and data analysis.** The initial model of the FMDV-O18-pO18-10 complex was constructed by rigid-body fitting the previously determined FMDV-O18 structure (PDB:8Y0Q) [6] and an AlphaFold2-predicted model of pO18-10 [39] into the cryo-EM density map using UCSF Chimera [40]. The complex model was then iteratively refined through cycles of real-space refinement in Phenix [41] and manual adjustment in Coot [42]. The refinement was performed until convergence as evaluated by model-to-map fit with valid geometrical parameters. The final model was validated using MolProbity [43]. The refinement statistics are presented in Table A in S1 Text.

**Rescue of site-directed FMDV mutants by reverse genetics.** Full-length cDNAs of FMDV serotype O were generated using an existing pOFS plasmid that contained the entire P1 gene of O/18074. Full-length cDNAs of FMDV serotype A were generated using an existing pQQN plasmid with the entire P1 gene of A/WH/CHA/09. Site-directed mutagenesis was applied to introduce nucleic acid mutations to produce full-length cDNAs with single amino-acid substitutions [44]. All mutant constructs were confirmed by nucleotide sequencing. The site-directed FMDV mutant viruses were rescued as previously described [45]. Briefly, *Not* I-linearized mutant plasmids were transfected into BSR/T7 cells using Lipofectamine 2000 according to the manufacturer’s instructions. The transfected cells were monitored daily for CPE appearance. At 72 h post-transfection, the cells were harvested and passaged in BHK-21 cells. After 3 rounds of passaging, the mutant virus titers were determined in BHK cells by calculating the 50% tissue culture infectious dose (TCID_50_), subsequently used to perform microneutralization assay as described above.

**Inhibition of virus attachment assay.** Different amounts of pO18-10 and control mAb (PLY57) were mixed with 5 × 10^5^ TCID_50_s of FMDV (O/18074 strain or A/WH/CHA/09 strain) in MEM medium, followed by incubation at 37˚C for 1 h. The mixtures were loaded on pre-cooling BHK-21 cells (approximately 5 × 10^5^ cells per treatment) and incubated at 4˚C for 1 h to allow virus attachment. After three thorough washes with cold PBS to remove unbound virus, the cells were further cultured for a duration of 4 h to enhance quantitation of the virus, then the final cells were harvested and examined by immunofluorescence staining, WB, and qRT-PCR assays. For the post-attachment inhibition assay, the BHK-21 cells were pre-adsorbed with equal amount of virus, and then were incubated with different amounts of pO18-10 and control mAb (PLY57) at 37˚C for 1 h. After washing with cold PBS to remove unbound virus, the cells were further cultured for a duration of 4 h, subsequently the samples were collected and analyzed as below. Immunofluorescence staining was performed in 24-well plate, and added to cattle-derived FMDV-specific antibody (E32) 200 μl/well at a concentration of 5 μg/ml for 1 h at 37˚C, followed by incubation with rabbit anti-cattle FITC-IgG (Thermo-Fisher, USA) at a 1:1,000 dilution for 1 h at 37˚C. Plates were washed three times with PBS and observed under an FL Imaging System (Life Technology, USA). WB was performed to 12% sodium dodecyl sulfate-polyacrylamide gel electrophoresis (SDS-PAGE) and electro-transferred to a methanol-activated PVDF membrane, and blocking with 5% non-fat milk in PBS overnight at 4˚C. The membrane was probed with porcine-derived bnAb (pOA-20) diluted to 2 μg/ml and rabbit anti-β-actin polyclonal antibody at a 1:2,000 dilution. The expression levels of indicated proteins were normalized with GAPDH. The qRT-PCR assays were performed with a pair of FMDV-3D-gene specific primers (forward primer, ACTGGGTTTTAYAAACCTGTGATG; reverse primer, TCAACTTCTCCTGKATGGTCCCA), and a pair of GAPDH-specific primers (forward primer: TGACGTGCCGCCTGGAGAAA; reverse primer: AGTGTAGCCCAAGATGCCCTTCAG). The relative expressions of FMDV-3D gene were normalized to β-actin. Data analysis was performed using the 2^-△△Ct^ method relative to the control group. The threshold cycle (C_T_) values of all samples were first normalized to the C_T_ value of β-actin and then compared to the C_T_ value of the control (samples treated with virus only). The inhibition efficiency of virus attachment for a given treatment group was defined as the FMDV-3D gene copy number for the group as a percentage of that for the control (which was treated with the virus only).

**Thermofluor assay.** Thermofluor experiments were performed using a RT-PCR instrument (ABI, Thermo Fisher Scientific) to evaluate FMDV 146S particle stability after incubation with each bnAb. In thin-walled PCR plates (ABI, Thermo Fisher Scientific), a 50-μl reaction volume was established using mixtures of 1.0 μg of 146S particles plus 1.5 μg antibody (∼60 antibody molecules per FMDV virion) and 5 μM SYTO9 (Invitrogen, USA). For all assays, the melt temperature was set from 25°C to 95°C in 0.5-°C increments with intervals of 1s. Fluorescence was evaluated with excitation and emission wavelengths of 490 nm and 516 nm, respectively. The dynamics of viral RNA release from virions with temperature were detected by increases in the fluorescence signal. Three independent assays were performed for each analysis. Data sets exported from the PCR machine were visualized using GraphPad Prism software version 9.5.1.

**Prophylactic and the therapeutic efficacy studies in Kunming (KM) suckling mice.** The protective efficacy of anti-FMDV bnAb was assessed in a KM suckling mice model of FMDV infection based on the mouse-adapted strain FMDV/O/18074. For the prophylactic experiment, groups of 2-day-old KM suckling mice were subcutaneously injected in the dorsal neck with 10, 5, 1, 0.1 or 0.01 mg/kg of pO18-10, or 10 mg/kg of control IgG pO18-36 (non-neutralizing mAb to FMDV). After 24 h, the mice were s.c. injected with 5,024 TCID_50_ of FMDV/O/18074 strain. For the therapeutic assay, groups of 2-day-old KM mice were s.c. injected with 5,024 TCID_50_ of FMDV/O/18074 strain. After 24 h, the mice were s.c. injected with 20, 10, 5, 1 or 0.1 mg/kg of pO18-10, or 20 mg/kg of control IgG pO18-36. For the prophylactic and therapeutic assays, all infected mice were observed daily for survival and clinical score for 10 days. Clinical scores were graded as follows: 0, healthy; 1, reduced mobility; 2, limb weakness; 3, limb paralysis; 4, death.

## Supporting information

S1 Text Fig A. Identification of the reactivity of porcine mAbs with different serotypes of FMDV using indirect immunofluorescence assay (IFA). BHK-21 cells infected respectively with multiple FMDV serotypes O, A and Asia1 strains, including O/18074, O/HN/CHA/93 strains (Cathay topotype), O/Tibet/99 strain (ME-SA topotype), O/GSLX/2010 and O/HNNY/2022 strains (SEA topotype), A/AF72 strain (A22 lineage), A/WH/CHA/09 and A/GDMM/2013 strains (SEA97 lineage) in Asia topotype and Asia1/JS/05 strain. The working concentration of the pO18-10 was 5µg/ml, followed by incubation with rabbit anti-pig FITC (diluted 1:200 in PBS). The cells were observed under an FL Imaging System (Life Technology, USA). The experiments were independently conducted in triplicate. **Fig B. Genetic stability of amino acid substitutions in neutralization-escape mutants during serial passaging in BHK-21 cells.** Nineteen neutralization-escape mutants were subjected to three serial passages in BHK-21 cells and sequenced. Panels **A-E** show the sequencing comparisons of mutants from pO18-10-O/18074 **(A)**, pO18-17-O/18074 **(B)**, pO18-52-O/HN/CHA/93 **(C)**, pO18-53-O/HN/CHA/93 **(D)**, and pO18-10-A/WH/CHA/09 **(E)**. The P1 region of each virus was amplified by one-step reverse transcription-PCR (RT-PCR) using primers Pan204+ (ACCTCCAACGGGTGGTACGC) and NK61 (GACATGTCCTCTTGCATCTG) and verified by sequencing. Amino acid substitutions were determined by alignment with the wild-type and parent virus sequence. In the panels, red boxes indicate amino acid substitutions, blue boxes indicate reversion substitutions, and green boxes indicate synonymous nucleotide substitutions. **Fig C. Structural features of the FMDV-O18-pO18-10 complex. (A)** Local resolution map of the intact FMDV-O18-pO18-10 complex reconstruction. **(B)** Cryo-EM density map for a single protomer in the complex, with atomic model fitted. **(C)** Structural comparison between the FMDV-O18-pO18-10 complex and the previously reported FMDV-O18 structure (PDB: 8Y0Q), with the VP1 C-terminal region magnified. **(D)** Close-up view of the VP1 C-terminus, showing the cryo-EM density and fitted model in both the FMDV-O18-pO18-10 complex and in 8Y0Q. **(E)** Key regions at the antibody-virus interface, with cryo-EM density and fitted models for the VP1 B-C loop, VP3 G-H loop, HCDR1, a segment of HCDR3, a segment of L-FR3. **Fig D. Antibody-FMDV complex structures and corresponding epitope footprints on the serotype O antigen.** Epitope footprints of the five mAbs pO18-10, pOA-2, R50, B77, and F145 mapped onto the surface of the serotype O FMDV antigen. VP1, VP2, VP3, and VP4 of the protomer are shown in blue, green, orange, and earthy yellow, respectively. Antibody-FMDV binding sites are highlighted in magenta. **Fig E. The recognized four cross-serotype antigenic structures of natural host bnAbs against FMDV.** Footprints of four cross-serotype antigenic sites on surface of three protomers of FMDV. One promoter comprising VP1, VP2, VP3 and VP4 was circled in grey/blue/red line. Cross-site 1 consisted of VP1 143, 145–148 and 151 position residues was marked in red. The related bnAbs pOA-1, pOA-6, pOA-7, pOA-8, pOA-9, pOA-13, pOA-17, pOA-19, and pOA-20 were identified from 216 clonotype antibodies shared between the porcine O/18074- and A/AF72-specific B cell libraries. Cross-site 2 that consisted of VP2 65, 68, 71, 72, 77 and 195 position residues on one protomer and VP3 68, 69, 70 and 195 position residues on another protomer, was marked in white. The related bnAb pOA-2 was identified from the same set of 216 shared clonotype antibodies derived from the porcine O/18074- and A/AF72-specific B cell libraries. Cross-site 3 that consisted of VP1 50, 52, 94, 95, 157, 159 and 160 position residues on one protomer and VP3 173 and 177 position residues on another protomer, was marked in blue. The related bnAb R50 was isolated from a natural bovine host using single B cell antibody isolation technology. Cross-site 4 that consisted of VP1 197, 198, 202, 204, 205, 210 and VP3 173 position residues on one protomer and VP1 95 and 99 position residues on another protomer, was marked in orange. The related bnAbs pO18-10, pO18-17, pO18-52, and pO18-53 were identified in this study from the porcine O/18074-specific B cell library. **Fig F. Identification of the rescued single-substitution mutants by plaque formation assay. (A)** The wild-type (O/18074) and rescued mutants (VP1 E95A, VP1 D99A, VP1 S197A, VP1 T198A, VP1 K204A, VP1 K210A and VP3 D173A) formed in BHK-21 cells, and the sizes were correlated to the CPE patterns. **(B)** The wild-type (A/WH/CHA/09 strain) and rescued mutants (VP1 D99A, VP1 K203A, VP1 K209A and VP3 D174A) formed in BHK-21 cells, and the sizes were correlated to the CPE patterns. **Fig G. Binding modes of FMDV integrin receptor and antibody.** Binding modes of FMDV integrin receptor (αvβ6) and bnAb pO18-10. Superposition of FMDV-αvβ6 with FMDV-O18-pO18-10. VP1, VP2, VP3, and VP4 of the protomer are shown in blue, green, orange, and earthy yellow, respectively. The αv and β6 chains of integrin (αvβ6) and pO18-10 are drawn in cartoon representation and colored in cyan, limon and magenta, respectively. Black dashed circles show significant clashes between antibody (pO18-10) and integrin receptor. **Fig H. Effect of pO18-10 on virus at the post-attachment stage.** The BHK-21 cells were respectively incubated with FMDV serotype O (O/18074 strain) **(A, C, E)** or serotype A (A/WH/CHA/09 strain) **(B, D, F)** at 4°C for 1h. Subsequently, the cells were treated with different amounts of pO18-10 at 4°C for 1h, the cells were washed with cold PBS to remove unbound virus, then the cells were further cultured for a duration of 4 hours. The viruses were quantified through detecting VP1 protein by IFA **(A, B)** and Western blotting **(C, D)** and 3D gene by qRT-PCR **(E, F)**. The experiments were independently conducted in triplicate. The data differences between conditions with virus only and different bnAb treatments were assessed using unpaired T-test (Holm-Sidak method, α = 0.05) in GraphPad Prism 9.5.1. *** Indicates an extremely significant difference at P < 0.001. ** Indicates a very significant difference at P < 0.01. * Indicates a significant difference at P < 0.05. NS indicates no significant difference. **Fig I. Validation of key pO18-10 residues against wild-type and mutant A/AF72 FMDV strains.** The wild-type (A/AF72) and rescued mutants (VP1 H99D and VP1 R204K) were generated in BHK-21 cells, and the neutralization efficacy of pO18-10 against both the wild-type and mutant viruses was evaluated using a microneutralization assay. **Table A. Cryo-EM data collection and refinement statistics. Table B. Interface identification and interaction analysis of pO18-10 with FMDV O/18074.**
(DOCX)

## References

[1] Perry BD, Rich KM. Poverty impacts of foot-and-mouth disease and the poverty reduction implications of its control. Vet Rec. 2007;160(7):238–41. doi: 10.1136/vr.160.7.238 17308024

[2] Zell R, Delwart E, Gorbalenya AE, Hovi T, King AMQ, Knowles NJ, et al. ICTV virus taxonomy profile: picornaviridae. J Gen Virol. 2017;98(10):2421–2. doi: 10.1099/jgv.0.000911 28884666 PMC5725991

[3] Domingo E, Escarmís C, Baranowski E, Ruiz-Jarabo CM, Carrillo E, Núñez JI, et al. Evolution of foot-and-mouth disease virus. Virus Res. 2003;91(1):47–63. doi: 10.1016/s0168-1702(02)00259-9 12527437

[4] Bachrach HL. Foot-and-mouth disease. Annu Rev Microbiol. 1968;22:201–44. doi: 10.1146/annurev.mi.22.100168.001221 4301615

[5] Kamel M, El-Sayed A, Castañeda Vazquez H. Foot-and-mouth disease vaccines: recent updates and future perspectives. Arch Virol. 2019;164(6):1501–13. doi: 10.1007/s00705-019-04216-x 30888563

[6] Li F, Wu S, Lv L, Huang S, Zhang Z, Zerang Z, et al. Discovery, recognized antigenic structures, and evolution of cross-serotype broadly neutralizing antibodies from porcine B-cell repertoires against foot-and-mouth disease virus. PLoS Pathog. 2024;20(10):e1012623. doi: 10.1371/journal.ppat.1012623 39405339 PMC11508087

[7] Belsham GJ, Kristensen T, Jackson T. Foot-and-mouth disease virus: prospects for using knowledge of virus biology to improve control of this continuing global threat. Virus Res. 2020;281:197909. doi: 10.1016/j.virusres.2020.197909 32126297

[8] McCahon D, Crowther JR, Belsham GJ, Kitson JD, Duchesne M, Have P, et al. Evidence for at least four antigenic sites on type O foot-and-mouth disease virus involved in neutralization; identification by single and multiple site monoclonal antibody-resistant mutants. J Gen Virol. 1989;70 (Pt 3):639–45. doi: 10.1099/0022-1317-70-3-639 2471793

[9] Pfaff E, Thiel HJ, Beck E, Strohmaier K, Schaller H. Analysis of neutralizing epitopes on foot-and-mouth disease virus. J Virol. 1988;62(6):2033–40. doi: 10.1128/JVI.62.6.2033-2040.1988 2835507 PMC253288

[10] Xie QC, McCahon D, Crowther JR, Belsham GJ, McCullough KC. Neutralization of foot-and-mouth disease virus can be mediated through any of at least three separate antigenic sites. J Gen Virol. 1987;68 (Pt 6):1637–47. doi: 10.1099/0022-1317-68-6-1637 2438378

[11] Aktas S, Samuel AR. Identification of antigenic epitopes on the foot and mouth disease virus isolate O1/Manisa/Turkey/69 using monoclonal antibodies. Rev Sci Tech. 2000;19(3):744–53. doi: 10.20506/rst.19.3.1244 11107617

[12] Barnett PV, Samuel AR, Pullen L, Ansell D, Butcher RN, Parkhouse RM. Monoclonal antibodies, against O1 serotype foot-and-mouth disease virus, from a natural bovine host, recognize similar antigenic features to those defined by the mouse. J Gen Virol. 1998;79 (Pt 7):1687–97. doi: 10.1099/0022-1317-79-7-1687 9680132

[13] Crowther JR, Farias S, Carpenter WC, Samuel AR. Identification of a fifth neutralizable site on type O foot-and-mouth disease virus following characterization of single and quintuple monoclonal antibody escape mutants. J Gen Virol. 1993;74 (Pt 8):1547–53. doi: 10.1099/0022-1317-74-8-1547 8393912

[14] Zhao Q, Li F, Huang S, Xing X, Sun Y, Li P, et al. Remapping the spatial distribution of neutralizing sites and their immunodominance on the capsid of different topotypes of FMDV serotype O by site-directed competitive ELISA for detection of neutralizing antibodies. Microbiol Spectr. 2025. doi: 10.1128/spectrum.03344-24:e0334424 PMC12131818 40372081

[15] Cao Y, Li F, Xing X, Zhang H, Zhao Q, Sun P, et al. Preparation and application of porcine broadly neutralizing monoclonal antibodies in an immunoassay for efficiently detecting neutralizing antibodies against foot-and-mouth disease virus serotype O. Microbiol Spectr. 2025;13(2):e0223424. doi: 10.1128/spectrum.02234-24 39772731 PMC11792482

[16] He Y, Li K, Cao Y, Sun Z, Li P, Bao H, et al. Structures of Foot-and-mouth Disease Virus with neutralizing antibodies derived from recovered natural host reveal a mechanism for cross-serotype neutralization. PLoS Pathog. 2021;17(4):e1009507. doi: 10.1371/journal.ppat.1009507 33909694 PMC8081260

[17] Dong H, Liu P, Bai M, Wang K, Feng R, Zhu D, et al. Structural and molecular basis for foot-and-mouth disease virus neutralization by two potent protective antibodies. Protein Cell. 2022;13(6):446–53. doi: 10.1007/s13238-021-00828-9 33599962 PMC9095805

[18] Hewat EA, Verdaguer N, Fita I, Blakemore W, Brookes S, King A, et al. Structure of the complex of an Fab fragment of a neutralizing antibody with foot-and-mouth disease virus: positioning of a highly mobile antigenic loop. EMBO J. 1997;16(7):1492–500. doi: 10.1093/emboj/16.7.1492 9130694 PMC1169753

[19] Mateu MG, Rocha E, Vicente O, Vayreda F, Navalpotro C, Andreu D, et al. Reactivity with monoclonal antibodies of viruses from an episode of foot-and-mouth disease. Virus Res. 1987;8(3):261–74. doi: 10.1016/0168-1702(87)90020-7 2446442

[20] Avendaño C, Celis-Giraldo C, Ordoñez D, Díaz-Arévalo D, Rodríguez-Habibe I, Oviedo J, et al. Evaluating the immunogenicity of chemically-synthesised peptides derived from foot-and-mouth disease VP1, VP2 and VP3 proteins as vaccine candidates. Vaccine. 2020;38(23):3942–51. doi: 10.1016/j.vaccine.2020.04.006 32307277

[21] Bachrach HL, Moore DM, McKercher PD, Polatnick J. Immune and antibody responses to an isolated capsid protein of foot-and-mouth disease virus. J Immunol. 1975;115(6):1636–41. doi: 10.4049/jimmunol.115.6.1636 171309

[22] Kleid DG, Yansura D, Small B, Dowbenko D, Moore DM, Grubman MJ, et al. Cloned viral protein vaccine for foot-and-mouth disease: responses in cattle and swine. Science. 1981;214(4525):1125–9. doi: 10.1126/science.6272395 6272395

[23] Mansuroğlu B, Derman S, Kızılbey K, Canım Ateş S, Mustafaeva Akdeste Z. Synthetic peptide vaccine for Foot-and-Mouth Disease: synthesis, characterization and immunogenicity. Turkish Journal of Biochemistry. 2020;45(6):859–68. doi: 10.1515/tjb-2020-0110

[24] Cao Y, Li K, Xing X, Zhu G, Fu Y, Bao H, et al. Development and validation of a competitive ELISA based on bovine monoclonal antibodies for the detection of neutralizing antibodies against foot-and-mouth disease virus serotype A. J Clin Microbiol. 2022;60(4):e0214221. doi: 10.1128/jcm.02142-21 35254106 PMC9020353

[25] Li K, He Y, Wang L, Li P, Wang S, Sun P, et al. Two cross-protective antigen sites on foot-and-mouth disease virus Serotype O structurally revealed by broadly neutralizing antibodies from cattle. J Virol. 2021;95(21):e0088121. doi: 10.1128/JVI.00881-21 34406868 PMC8513477

[26] Harmsen MM, van Solt CB, Fijten HPD, van Keulen L, Rosalia RA, Weerdmeester K, et al. Passive immunization of guinea pigs with llama single-domain antibody fragments against foot-and-mouth disease. Vet Microbiol. 2007;120(3–4):193–206. doi: 10.1016/j.vetmic.2006.10.029 17127019

[27] Qiu X, Lei Y, Yang P, Gao Q, Wang N, Cao L, et al. Structural basis for neutralization of Japanese encephalitis virus by two potent therapeutic antibodies. Nat Microbiol. 2018;3(3):287–94. doi: 10.1038/s41564-017-0099-x 29379207

[28] Blancou J. History of the control of foot and mouth disease. Comp Immunol Microbiol Infect Dis. 2002;25(5–6):283–96. doi: 10.1016/s0147-9571(02)00026-7 12365805

[29] McCullough KC, Crowther JR, Butcher RN, Carpenter WC, Brocchi E, Capucci L, et al. Immune protection against foot-and-mouth disease virus studied using virus-neutralizing and non-neutralizing concentrations of monoclonal antibodies. Immunology. 1986;58(3):421–8. 3015780 PMC1453459

[30] Wang CY, Chang TY, Walfield AM, Ye J, Shen M, Chen SP, et al. Effective synthetic peptide vaccine for foot-and-mouth disease in swine. Vaccine. 2002;20(19–20):2603–10. doi: 10.1016/s0264-410x(02)00148-2 12057619

[31] Cao N, Li Y, Zhao Q, Yao M, Ren X, Tian L, et al. Self-assembled nanoparticle vaccines elicit robust protective immune responses against type O foot-and-mouth disease virus infection. ACS Nano. 2025;19(37):33134–55. doi: 10.1021/acsnano.5c04881 40926523

[32] Lee G, Hwang J-H, Kim A, Park J-H, Lee MJ, Kim B, et al. Analysis of amino acid mutations of the foot-and-mouth disease virus Serotype O using both heparan sulfate and JMJD6 receptors. Viruses. 2020;12(9):1012. doi: 10.3390/v12091012 32927791 PMC7551012

[33] Subramaniam S, Das B, Biswal JK, Ranjan R, Pattnaik B. Antigenic variability of foot-and-mouth disease virus serotype O during serial cytolytic passage. Virus Genes. 2017;53(6):931–4. doi: 10.1007/s11262-017-1494-3 28718047

[34] Li K, Zhu G, Zhou S, Sun P, Wang H, Bao H, et al. Isolation and characterization of porcine monoclonal antibodies revealed two distinct serotype-independent epitopes on VP2 of foot-and-mouth disease virus. J Gen Virol. 2021;102(7):10.1099/jgv.0.001608. doi: 10.1099/jgv.0.001608 34280085

[35] Li K, Wang S, Cao Y, Bao H, Li P, Sun P, et al. Development of foot-and-mouth disease virus-neutralizing monoclonal antibodies derived from plasmablasts of infected cattle and their germline gene usage. Front Immunol. 2019;10:2870. doi: 10.3389/fimmu.2019.02870 31867017 PMC6908506

[36] Bai X, Bao H, Li P, Sun P, Kuang W, Cao Y, et al. Genetic characterization of the cell-adapted PanAsia strain of foot-and-mouth disease virus O/Fujian/CHA/5/99 isolated from swine. Virol J. 2010;7:208. doi: 10.1186/1743-422X-7-208 20807416 PMC2939563

[37] Punjani A, Rubinstein JL, Fleet DJ, Brubaker MA. cryoSPARC: algorithms for rapid unsupervised cryo-EM structure determination. Nat Methods. 2017;14(3):290–6. doi: 10.1038/nmeth.4169 28165473

[38] Mindell JA, Grigorieff N. Accurate determination of local defocus and specimen tilt in electron microscopy. J Struct Biol. 2003;142(3):334–47. doi: 10.1016/s1047-8477(03)00069-8 12781660

[39] Jumper J, Evans R, Pritzel A, Green T, Figurnov M, Ronneberger O, et al. Highly accurate protein structure prediction with AlphaFold. Nature. 2021;596(7873):583–9. doi: 10.1038/s41586-021-03819-2 34265844 PMC8371605

[40] Pettersen EF, Goddard TD, Huang CC, Couch GS, Greenblatt DM, Meng EC, et al. UCSF Chimera--a visualization system for exploratory research and analysis. J Comput Chem. 2004;25(13):1605–12. doi: 10.1002/jcc.20084 15264254

[41] Liebschner D, Afonine PV, Baker ML, Bunkóczi G, Chen VB, Croll TI, et al. Macromolecular structure determination using X-rays, neutrons and electrons: recent developments in Phenix. Acta Crystallogr D Struct Biol. 2019;75(Pt 10):861–77. doi: 10.1107/S2059798319011471 31588918 PMC6778852

[42] Emsley P, Lohkamp B, Scott WG, Cowtan K. Features and development of Coot. Acta Crystallogr D Biol Crystallogr. 2010;66(Pt 4):486–501. doi: 10.1107/S0907444910007493 20383002 PMC2852313

[43] Williams CJ, Headd JJ, Moriarty NW, Prisant MG, Videau LL, Deis LN, et al. MolProbity: More and better reference data for improved all-atom structure validation. Protein Sci. 2018;27(1):293–315. doi: 10.1002/pro.3330 29067766 PMC5734394

[44] Bai XW, Bao HF, Li PH, Ma XQ, Sun P, Bai QF, et al. 2019. Engineering responses to amino acid substitutions in the VP0- and VP3-coding regions of PanAsia-1 strains of foot-and-mouth disease virus serotype O. J Virol. 2019;93. 10.1128/JVI.02278-18 PMC6430551 30700601

[45] Li P, Bai X, Sun P, Li D, Lu Z, Cao Y, et al. Evaluation of a genetically modified foot-and-mouth disease virus vaccine candidate generated by reverse genetics. BMC Vet Res. 2012;8:57. doi: 10.1186/1746-6148-8-57 22591597 PMC3488552

[46] Xiao C, Rossmann MG. Interpretation of electron density with stereographic roadmap projections. J Struct Biol. 2007;158(2):182–7. doi: 10.1016/j.jsb.2006.10.013 17116403 PMC1978246

[47] Winn MD, Ballard CC, Cowtan KD, Dodson EJ, Emsley P, Evans PR, et al. Overview of the CCP4 suite and current developments. Acta Crystallogr D Biol Crystallogr. 2011;67(Pt 4):235–42. doi: 10.1107/S0907444910045749 21460441 PMC3069738

