# Supplementary material for: Structural basis of a conserved, broad antigenic region surrounding the five-fold axis of foot-and-mouth disease virus revealed by swine neutralizing antibodies
Source: PLoS Pathog. 2026 Jul 14;22(7):e1014357. doi: 10.1371/journal.ppat.1014357 (PMC13367737; doi:10.1371/journal.ppat.1014357)
Supplement: S1 Text — BHK-21 cells infected respectively with multiple FMDV serotypes O, A and Asia1 strains, including O/18074, O/HN/CHA/93 strains (Cathay topotype), O/Tibet/99 strain (ME-SA topotype), O/GSLX/2010 and O/HNNY/2022 strains (SEA topotype), A/AF72 strain (A22 lineage), A/WH/CHA/09 and A/GDMM/2013 strains (SEA97 lineage) in Asia topotype and Asia1/JS/05 strain. The working concentration of the pO18-10 was 5µg/ml, followed by incubation with rabbit anti-pig FITC (diluted 1:200 in PBS). The cells were observed under an FL Imaging System (Life Technology, USA). The experiments were independently conducted in triplicate. Fig B. Genetic stability of amino acid substitutions in neutralization-escape mutants during serial passaging in BHK-21 cells. Nineteen neutralization-escape mutants were subjected to three serial passages in BHK-21 cells and sequenced. Panels A-E show the sequencing comparisons of mutants from pO18-10-O/18074 (A), pO18-17-O/18074 (B), pO18-52-O/HN/CHA/93 (C), pO18-53-O/HN/CHA/93 (D), and pO18-10-A/WH/CHA/09 (E). The P1 region of each virus was amplified by one-step reverse transcription-PCR (RT-PCR) using primers Pan204+ (ACCTCCAACGGGTGGTACGC) and NK61 (GACATGTCCTCTTGCATCTG) and verified by sequencing. Amino acid substitutions were determined by alignment with the wild-type and parent virus sequence. In the panels, red boxes indicate amino acid substitutions, blue boxes indicate reversion substitutions, and green boxes indicate synonymous nucleotide substitutions. Fig C. Structural features of the FMDV-O18-pO18-10 complex. (A) Local resolution map of the intact FMDV-O18-pO18-10 complex reconstruction. (B) Cryo-EM density map for a single protomer in the complex, with atomic model fitted. (C) Structural comparison between the FMDV-O18-pO18-10 complex and the previously reported FMDV-O18 structure (PDB: 8Y0Q), with the VP1 C-terminal region magnified. (D) Close-up view of the VP1 C-terminus, showing the cryo-EM density and fitted model in both the FMDV-O18-pO18- [file ppat.1014357.s001.docx]

**Supplementary Figures**


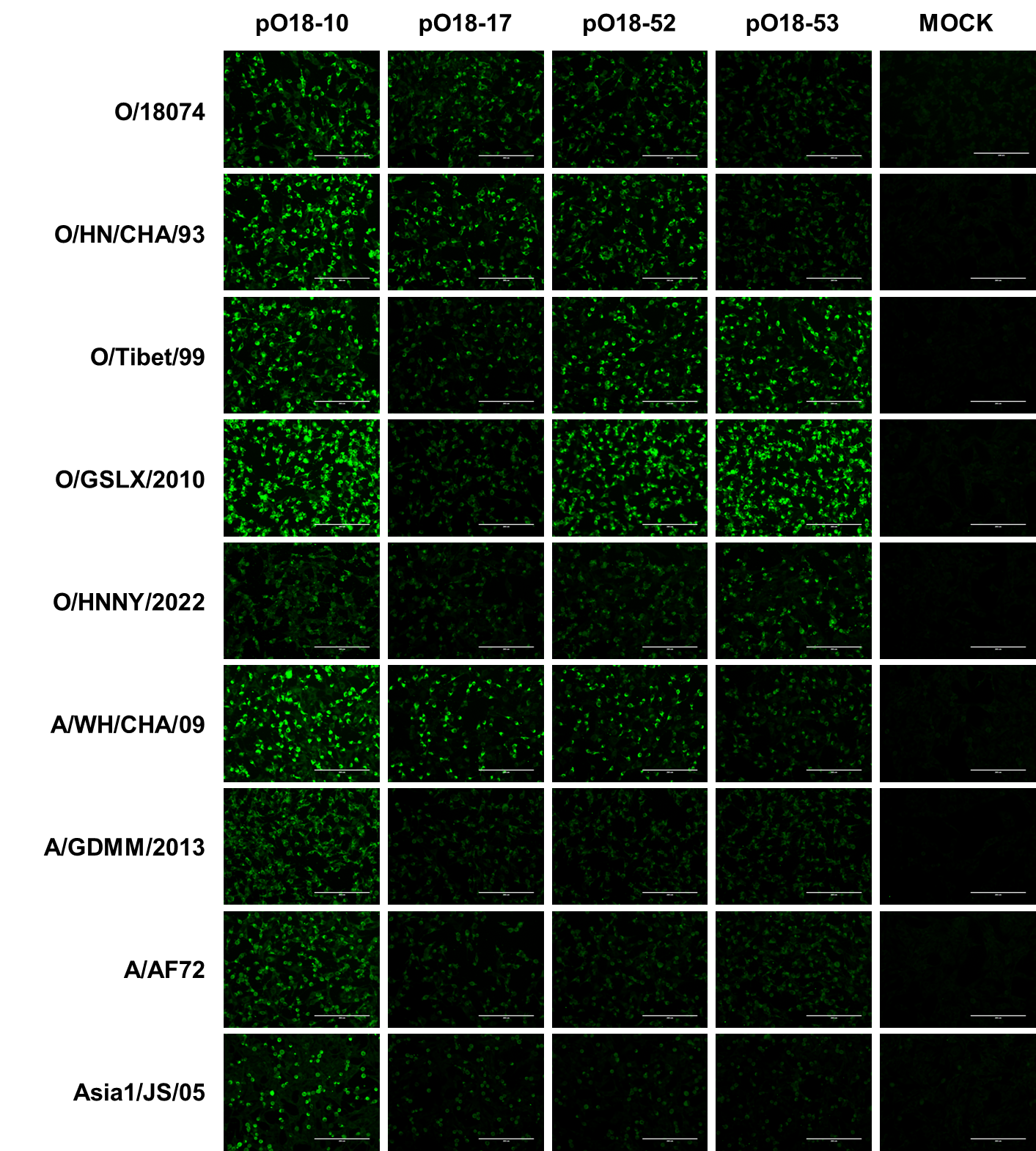


**Fig A.** **Identification of the reactivity of porcine mAbs with different serotypes of FMDV using indirect immunofluorescence assay (IFA).** BHK-21 cells infected respectively with multiple FMDV serotypes O, A and Asia1 strains, including O/18074, O/HN/CHA/93 strains (Cathay topotype), O/Tibet/99 strain (ME-SA topotype), O/GSLX/2010 and O/HNNY/2022 strains (SEA topotype), A/AF72 strain (A22 lineage), A/WH/CHA/09 and A/GDMM/2013 strains (SEA97 lineage) in Asia topotype and Asia1/JS/05 strain. The working concentration of the pO18-10 was 5µg/ml, followed by incubation with rabbit anti-pig FITC (diluted 1:200 in PBS). The cells were observed under an EVOS® FL Imaging System (Life Technology, USA). The experiments were independently conducted in triplicate.

**
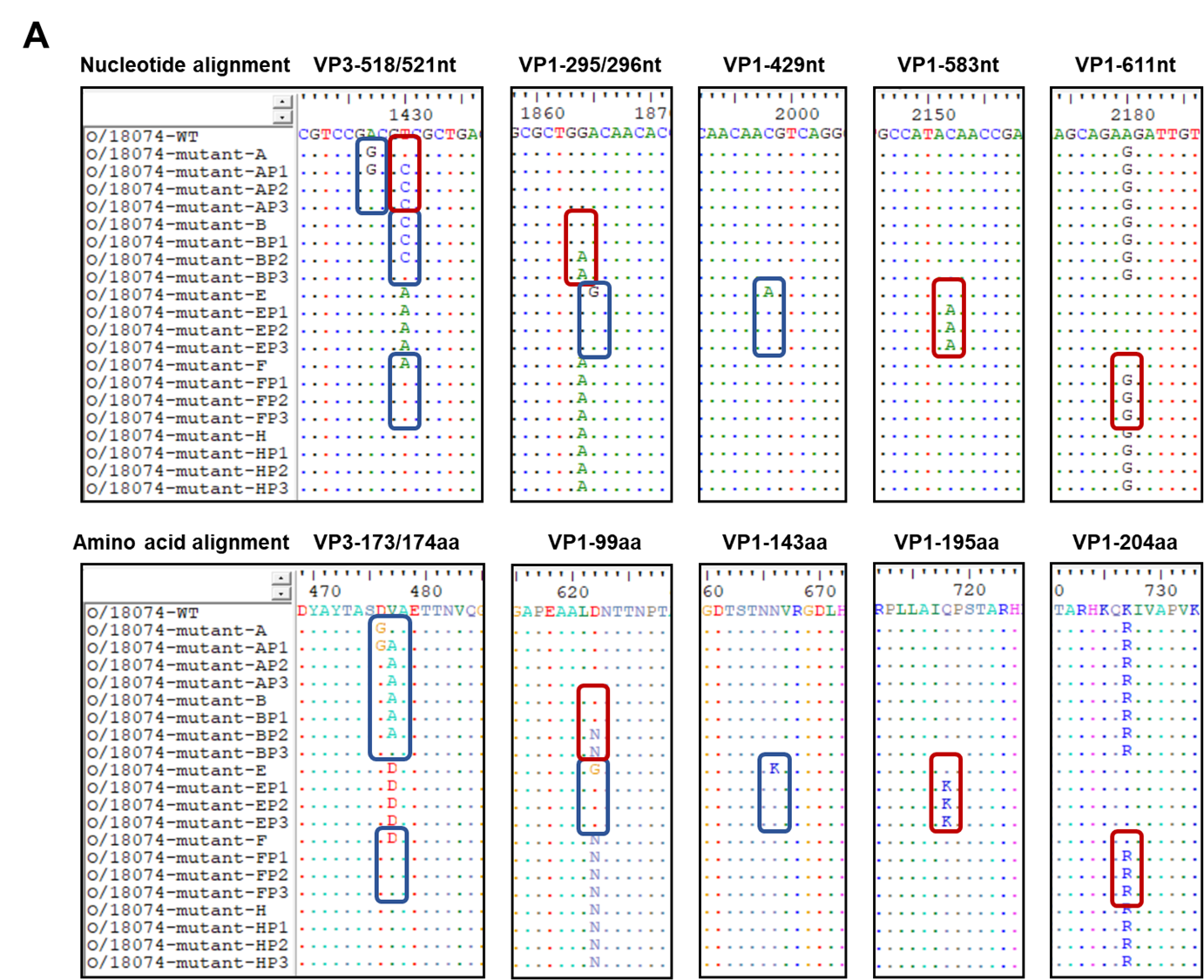
**

**
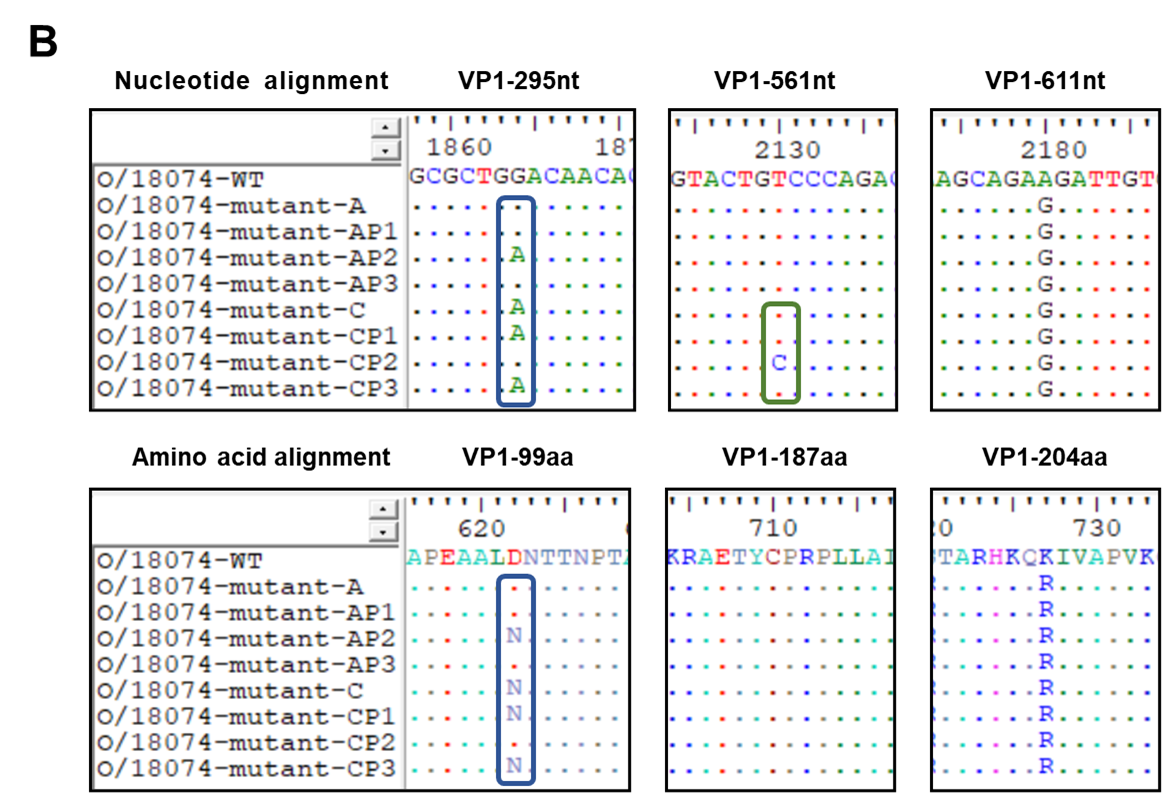
**


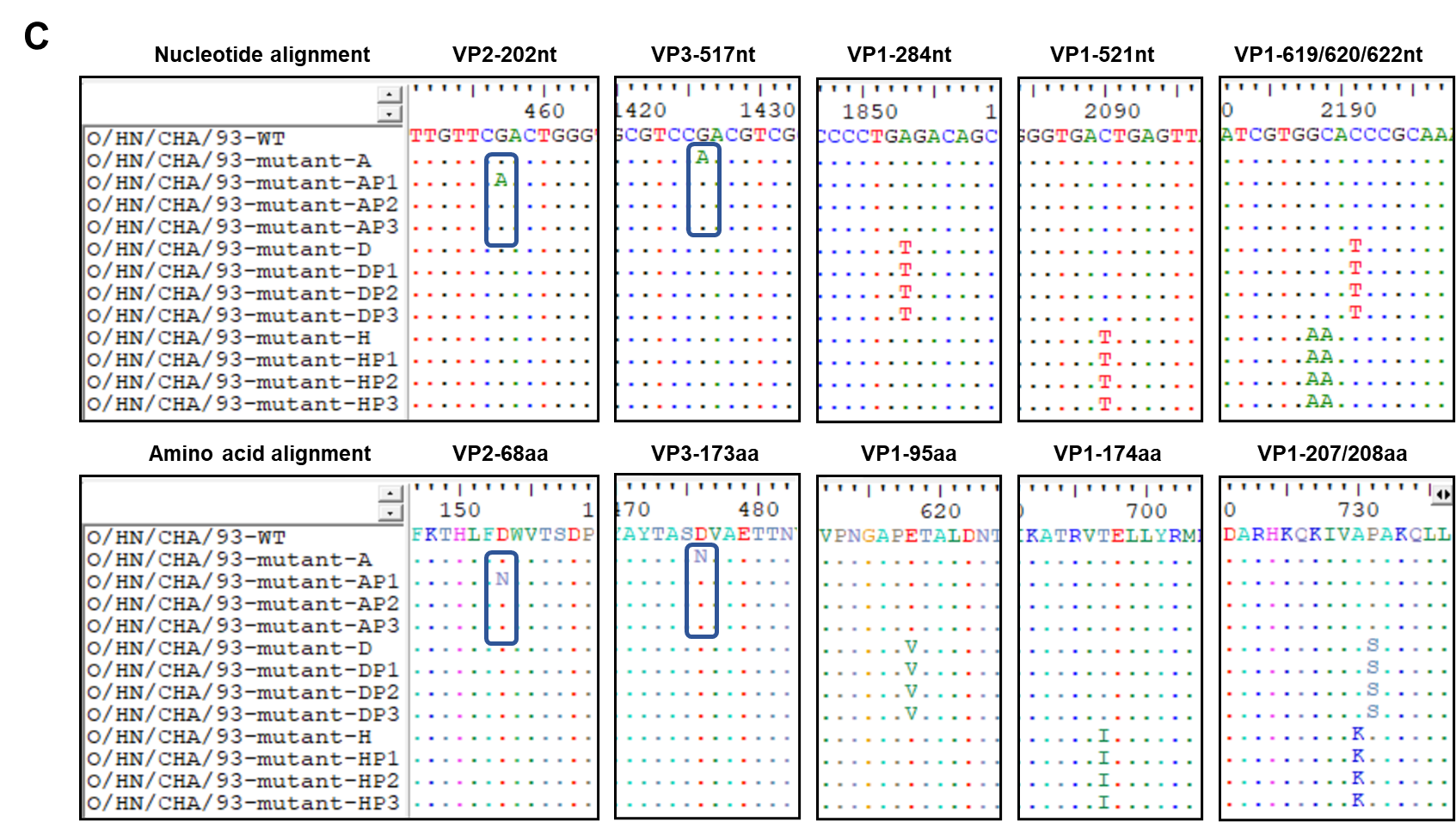


**
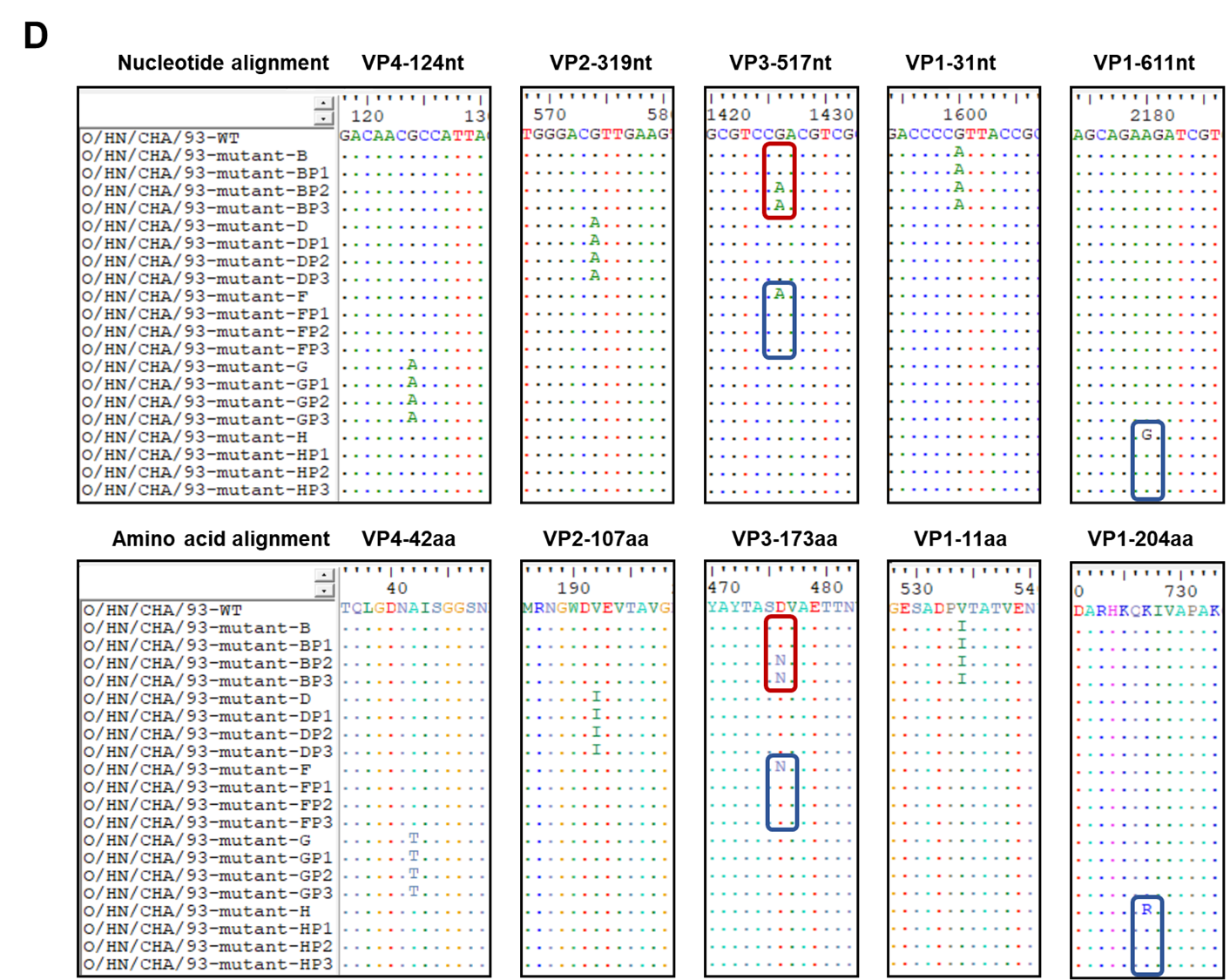
**


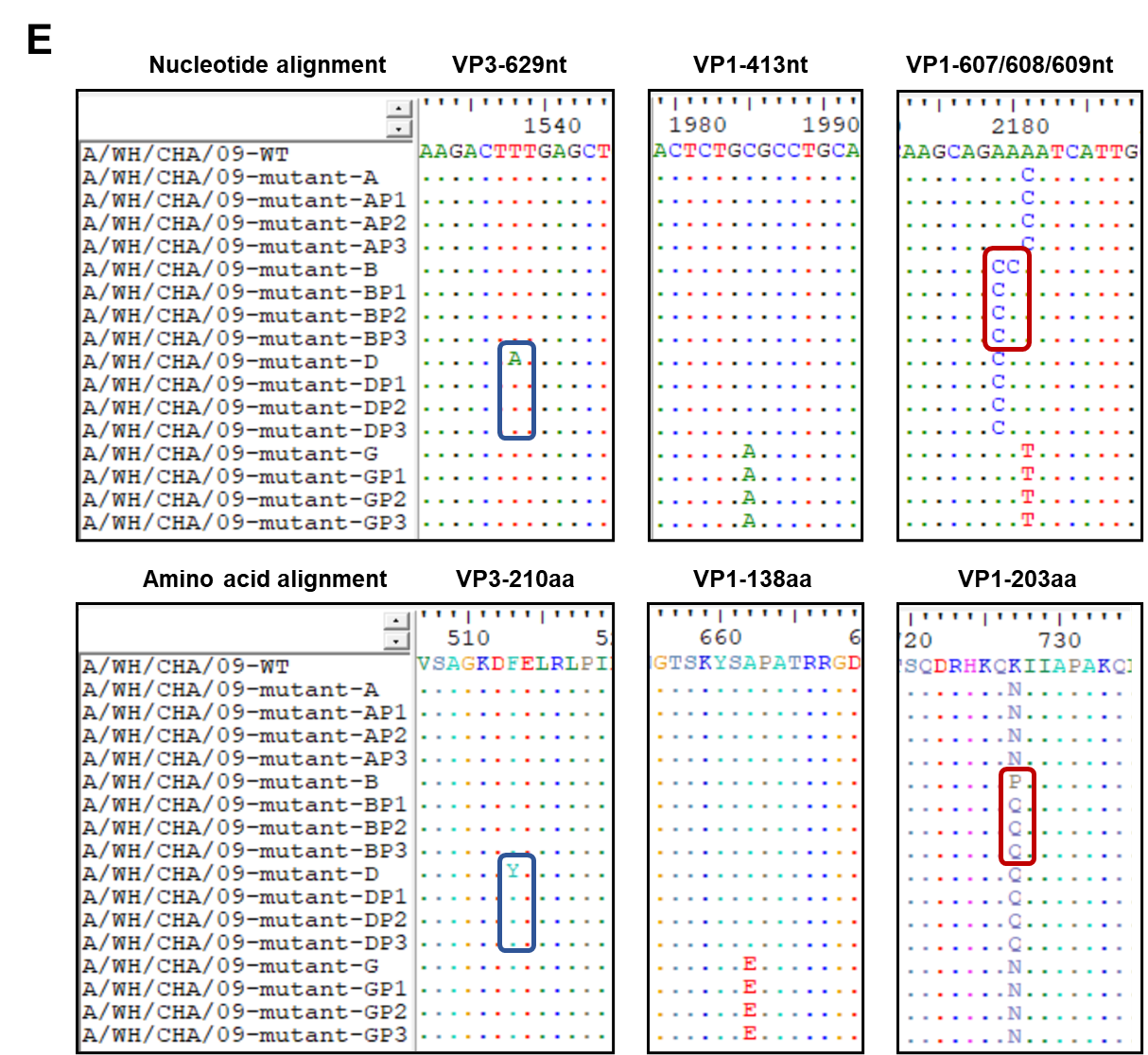


**Fig B. Genetic stability of amino acid substitutions in neutralization-escape mutants during serial passaging** **in BHK-21 cells.** Nineteen neutralization-escape mutants were subjected to three serial passages in BHK-21 cells and sequenced. Panels **A-E** show the sequencing comparisons of mutants from pO18-10-O/18074 **(A)**, pO18-17-O/18074 **(B)**, pO18-52-O/HN/CHA/93 **(C)**, pO18-53-O/HN/CHA/93 **(D)**, and pO18-10-A/WH/CHA/09 **(E)**. The P1 region of each virus was amplified by one-step reverse transcription-PCR (RT-PCR) using primers Pan204+ (ACCTCCAACGGGTGGTACGC) and NK61 (GACATGTCCTCTTGCATCTG) and verified by sequencing. Amino acid substitutions were determined by alignment with the wild-type and parent virus sequence. In the panels, red boxes indicate amino acid substitutions, blue boxes indicate reversion substitutions, and green boxes indicate synonymous nucleotide substitutions.


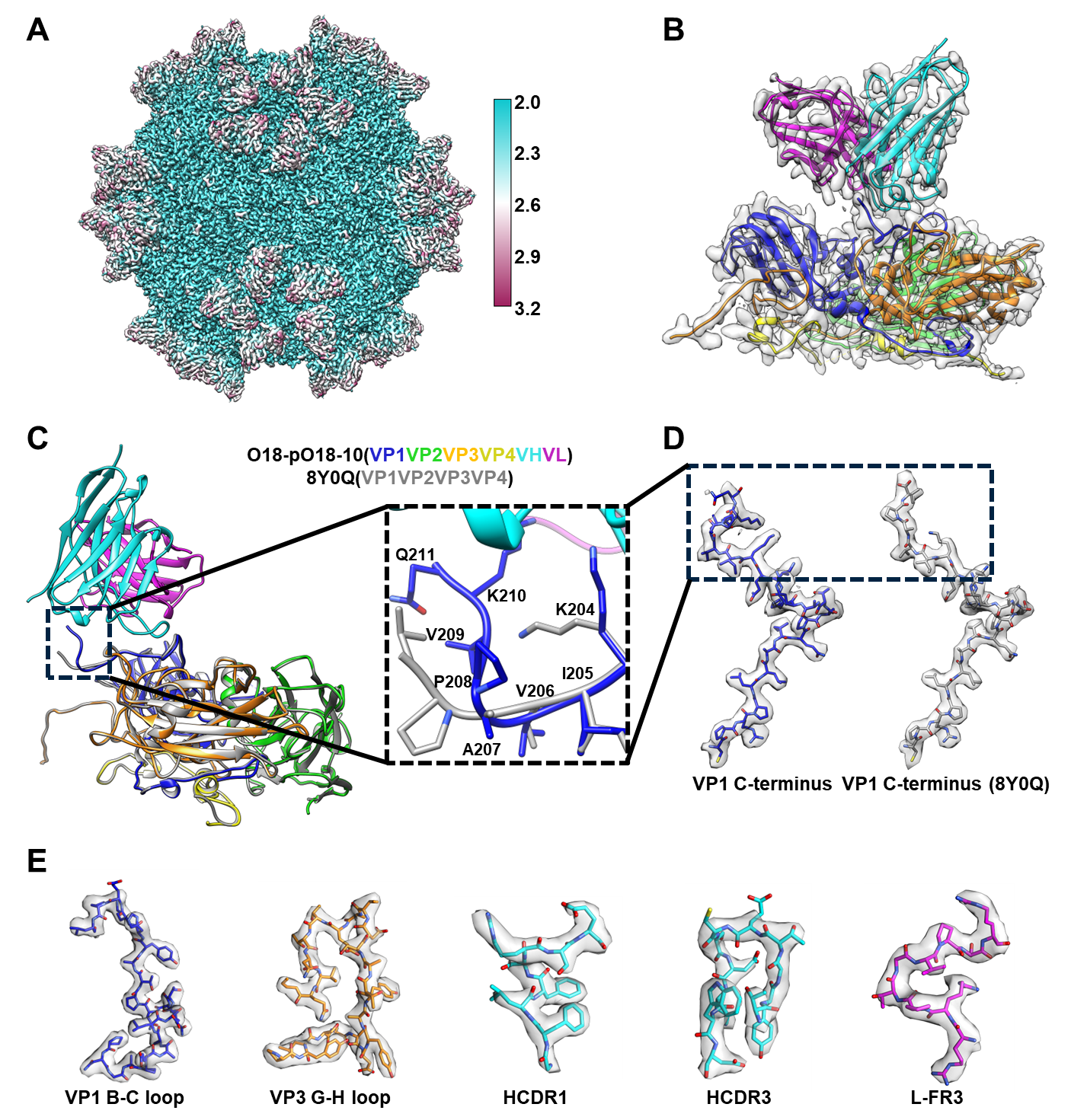


**Fig C. Structural features of the FMDV-O18-pO18-10 complex.** **(A)** Local resolution map of the intact FMDV-O18-pO18-10 complex reconstruction. **(B)** Cryo-EM density map for a single protomer in the complex, with atomic model fitted. **(C)** Structural comparison between the FMDV-O18-pO18-10 complex and the previously reported FMDV-O18 structure (PDB: 8Y0Q), with the VP1 C-terminal region magnified. **(D)** Close-up view of the VP1 C-terminus, showing the cryo-EM density and fitted model in both the FMDV-O18-pO18-10 complex and in 8Y0Q. **(E)** Key regions at the antibody-virus interface, with cryo-EM density and fitted models for the VP1 B-C loop, VP3 G-H loop, HCDR1, a segment of HCDR3, a segment of L-FR3.

**
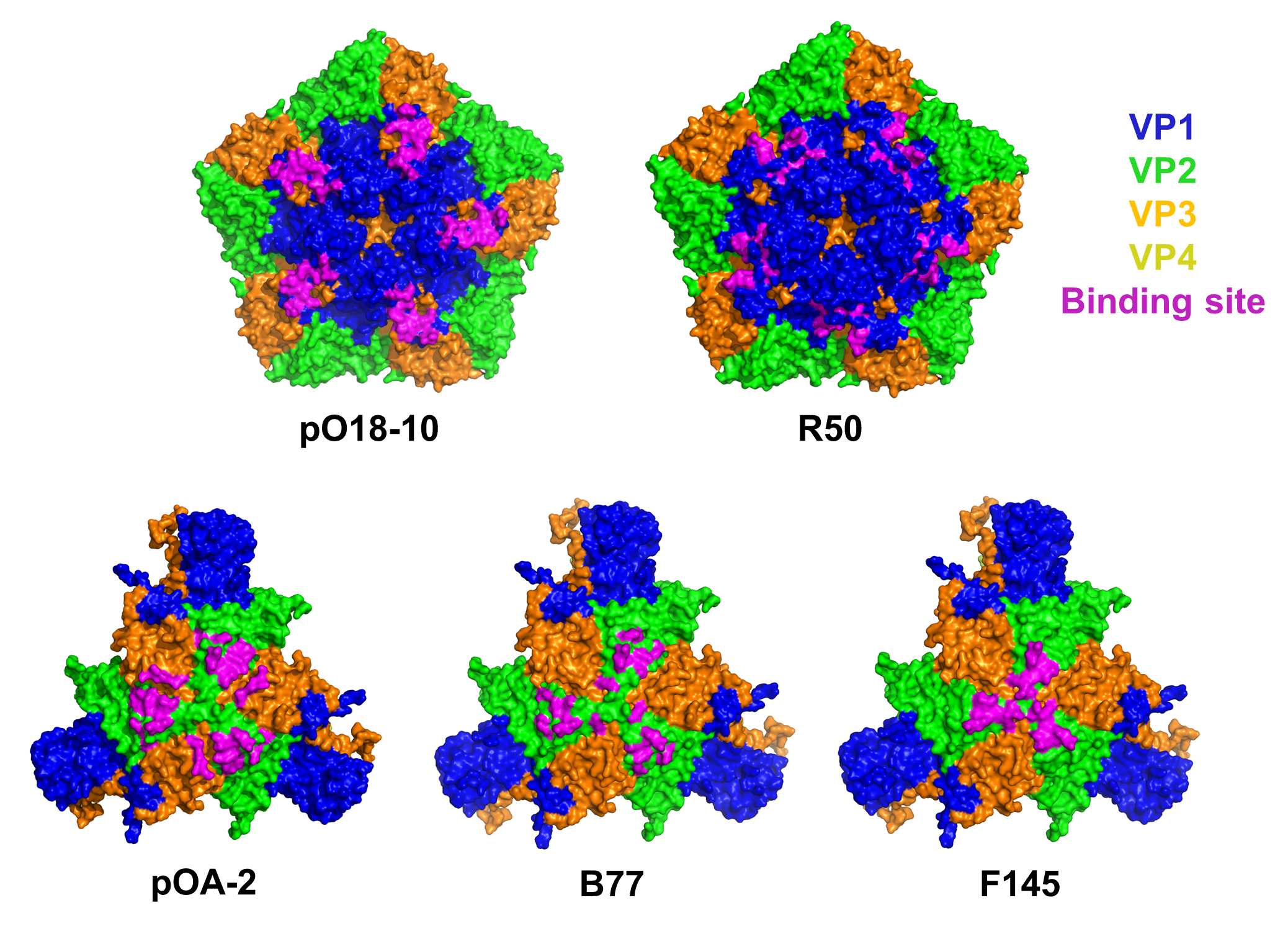
**

**Fig D. Antibody-FMDV complex structures and corresponding epitope footprints on the serotype O antigen.** Epitope footprints of the five mAbs pO18-10, pOA-2, R50, B77, and F145 mapped onto the surface of the serotype O FMDV antigen. VP1, VP2, VP3, and VP4 of the protomer are shown in blue, green, orange, and earthy yellow, respectively. Antibody-FMDV binding sites are highlighted in magenta.


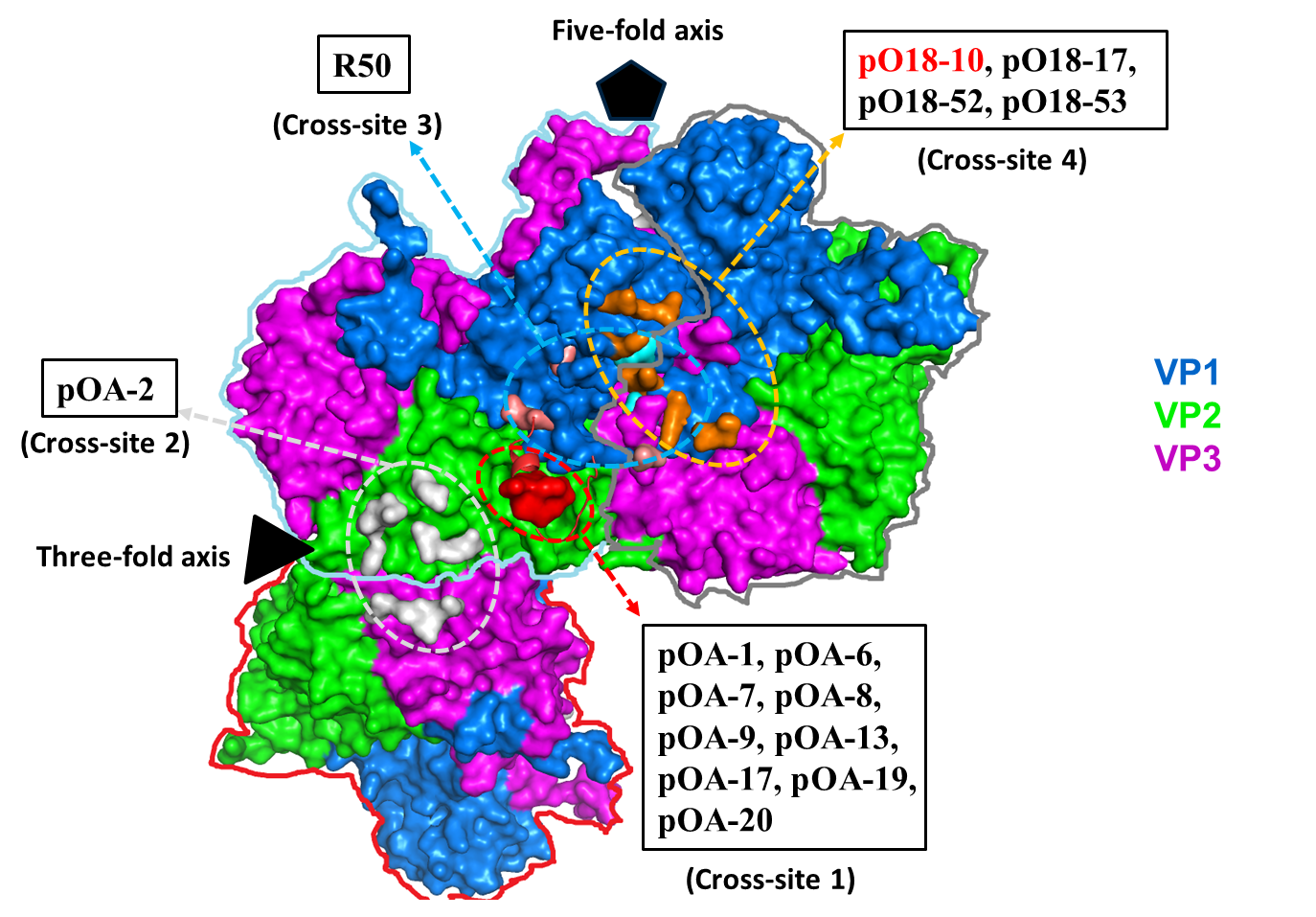


**Fig E.** **The recognized four cross-serotype antigenic structures of natural host bnAbs against FMDV.** Footprints of four cross-serotype antigenic sites on the surface of three protomers of FMDV. One protomer comprising VP1, VP2, VP3 and VP4 was circled in grey/blue/red line. Cross- site 1 consisted of VP1 143, 145-148 and 151 position residues was marked in red. The related bnAbs pOA-1, pOA-6, pOA-7, pOA-8, pOA-9, pOA-13, pOA-17, pOA-19, and pOA-20 were identified from 216 clonotype antibodies shared between the porcine O/18074- and A/AF72-specific B cell libraries. Cross-site 2 that consisted of VP2 65, 68, 71, 72, 77 and 195 position residues on one protomer and VP3 68, 69, 70 and 195 position residues on another protomer, was marked in white. The related bnAb pOA-2 was identified from the same set of 216 shared clonotype antibodies derived from the porcine O/18074- and A/AF72-specific B cell libraries. Cross-site 3 that consisted of VP1 50, 52, 94, 95, 157, 159 and 160 position residues on one protomer and VP3 173 and 177 position residues on another protomer, was marked in blue. The related bnAb R50 was isolated from a natural bovine host using single B cell antibody isolation technology. Cross-site 4 that consisted of VP1 197, 198, 202, 204, 205, 210 and VP3 173 position residues on one protomer and VP1 95 and 99 position residues on another protomer, was marked in orange. The related bnAbs pO18-10, pO18-17, pO18-52, and pO18-53 were identified in this study from the porcine O/18074-specific B cell library.


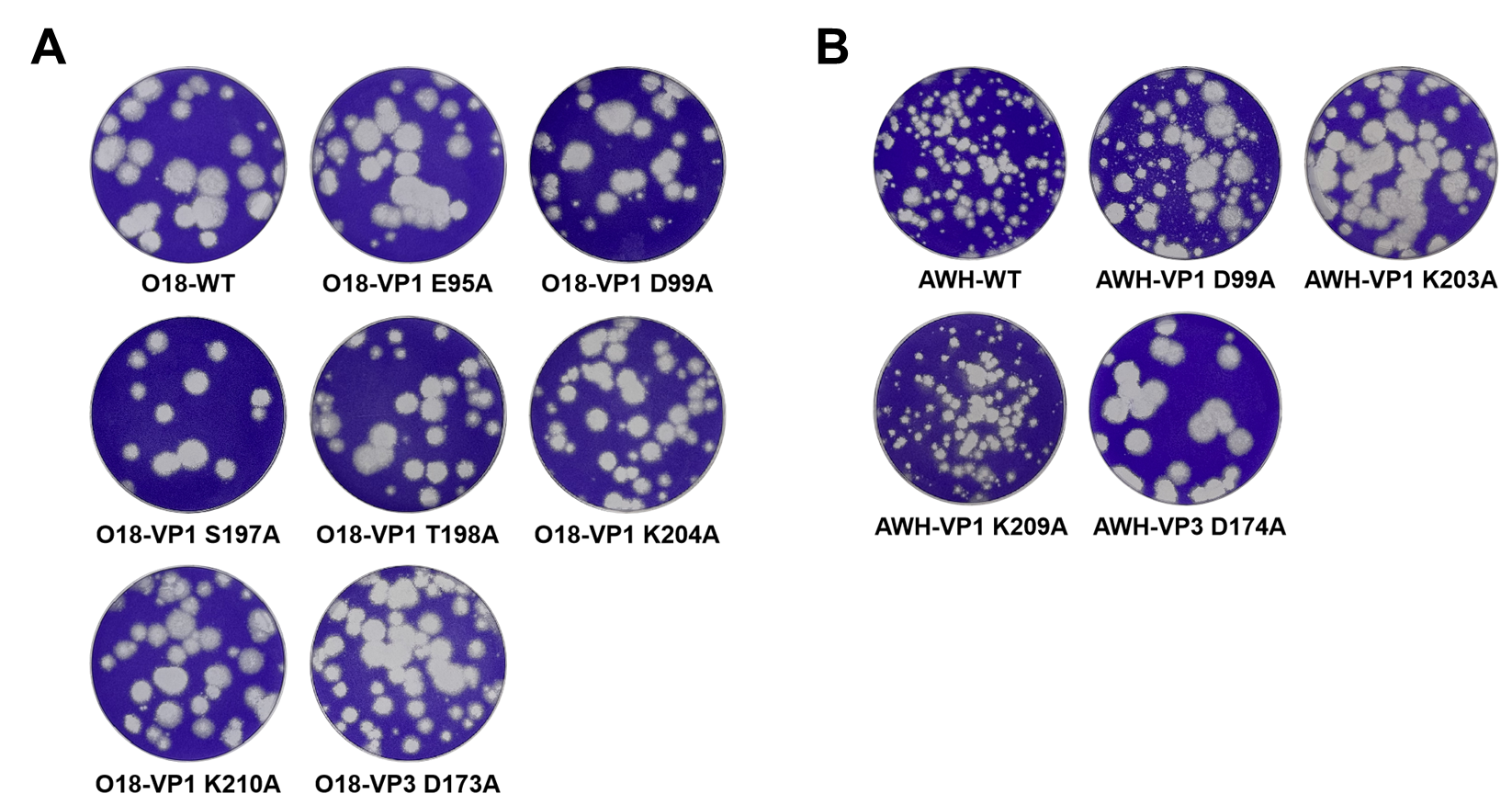


**Fig F.** **Identification of the rescued single-substitution mutants by plaque formation assay.** **(A)** The wild-type (O/18074) and rescued mutants (VP1 E95A, VP1 D99A, VP1 S197A, VP1 T198A, VP1 K204A, VP1 K210A and VP3 D173A) formed in BHK-21 cells, and the sizes were correlated to the CPE patterns. **(B)** The wild-type (A/WH/CHA/09 strain) and rescued mutants (VP1 D99A, VP1 K203A, VP1 K209A and VP3 D174A) formed in BHK-21 cells, and the sizes were correlated to the CPE patterns.


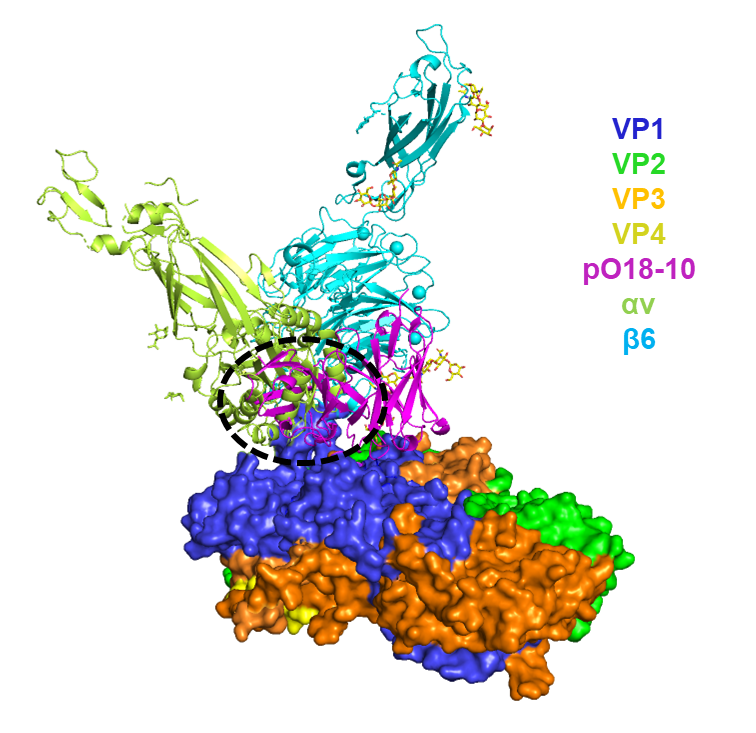


**Fig G. Binding modes of FMDV integrin receptor and antibody.** Binding modes of FMDV integrin receptor (αvβ6) and bnAb pO18-10. Superposition of FMDV-αvβ6 with FMDV-O18-pO18-10. VP1, VP2, VP3, and VP4 of the protomer are shown in blue, green, orange, and earthy yellow, respectively. The αv and β6 chains of integrin (αvβ6) and pO18-10 are drawn in cartoon representation and colored in cyan, light blue and magenta, respectively. Black dashed circles show significant clashes between antibody (pO18-10) and integrin receptor.


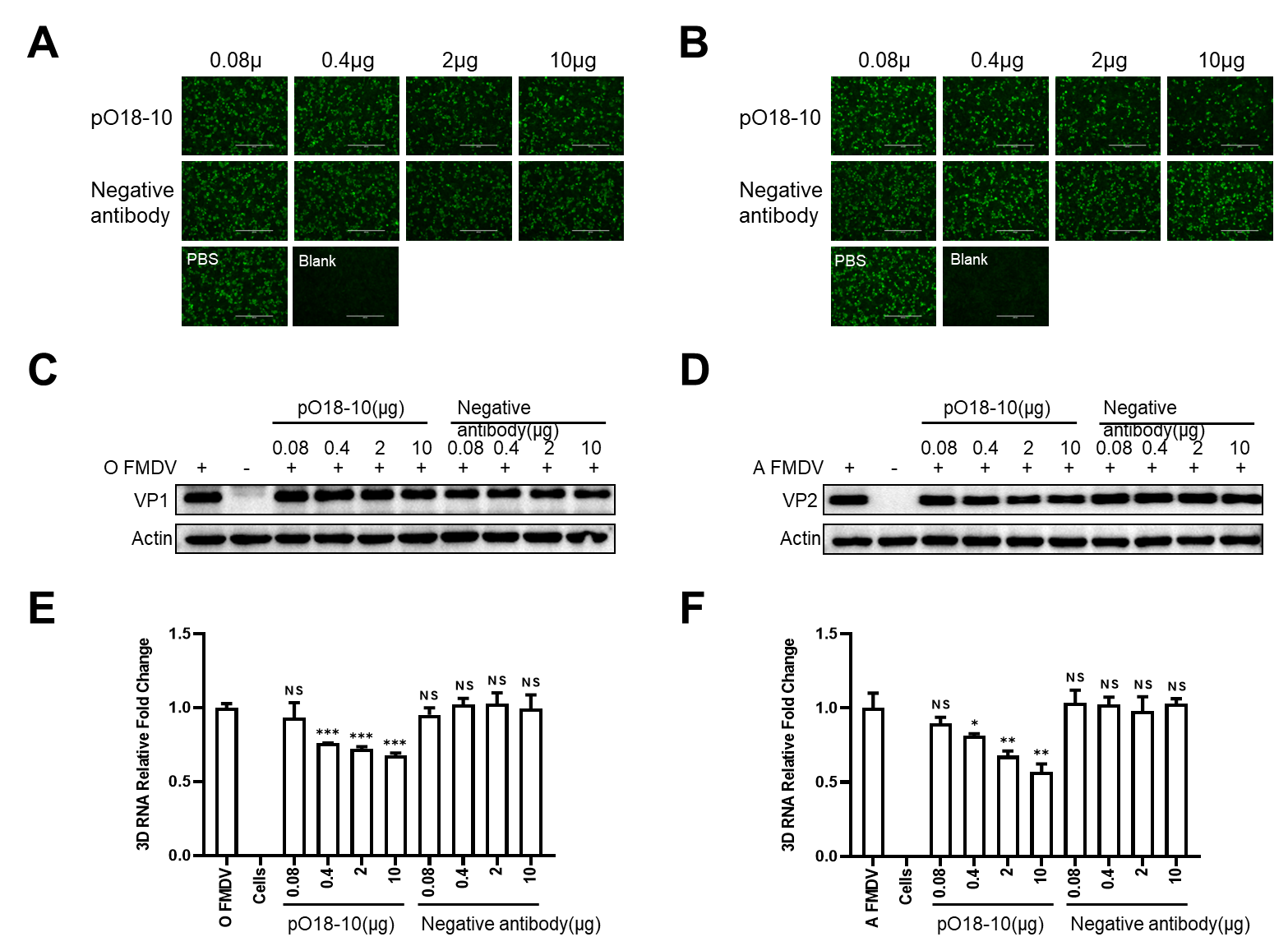
**Fig H.** **Effect of pO18-10 on virus at the post-attachment stage.** The BHK-21 cells were respectively incubated with FMDV serotype O (O/18074 strain) **(A, C, E)** or serotype A (A/WH/CHA/09 strain) **(B, D, F)** at 4°C for 1 h. Subsequently, the cells were treated with different amounts of pO18-10 at 4°C for 1 h, the cells were washed with cold PBS to remove unbound virus, then the cells were further cultured for 4 h. The viruses were quantified through detecting VP1 protein by IFA **(A, B)** and Western blotting **(C, D)** and 3D gene by qRT-PCR **(E, F)**. The experiments were independently conducted in triplicate. The data differences between conditions with virus only and different bnAb treatments were assessed using unpaired T-test (Holm-Sidak method, α = 0.05) in GraphPad Prism 9.5.1. *** Indicates an extremely significant difference at P<0.001. ** Indicates a very significant difference at P<0.01. * Indicates a significant difference at P<0.05. NS indicates no significant difference.

**
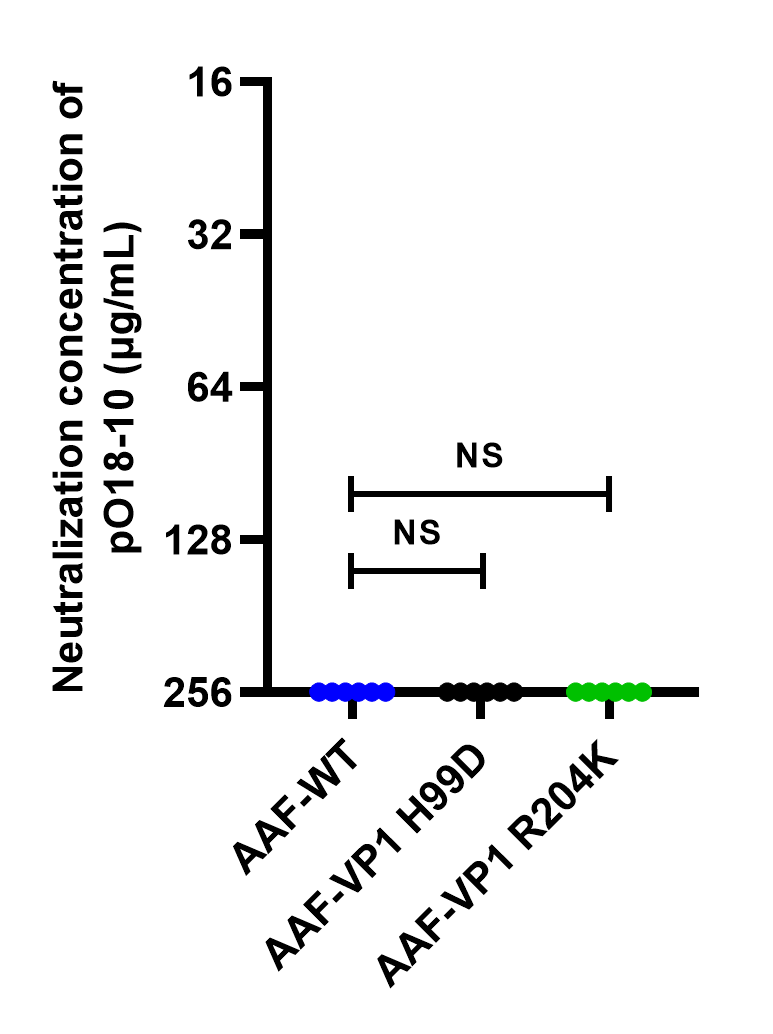
**

**Fig I. Validation of key pO18-10 residues against wild-type and mutant A/AF72 FMDV strains.** The wild-type (A/AF72) and rescued mutants (VP1 H99D and VP1 R204K) were generated in BHK-21 cells, and the neutralization efficacy of pO18-10 against both the wild-type and mutant viruses was evaluated using a microneutralization assay.

**Table A. Cryo-EM data collection and refinement statistics.**

| **Parameter** | **Value for FMDV-O18-pO18-10** |
| --- | --- |
| Data collection and processing |  |
| Magnification | 105,000 |
| Voltage (kV) | 300 |
| Electron exposure (e^–^/Å^2^) | 60 |
| Defocus range (*μ*m) | -2.0 to -1.0 |
| Pixel size (Å) | 0.83 |
| Symmetry imposed | I1 |
| Final particle images (no.) | 29102 |
| Map resolution (Å) | 2.27 |
| Map sharpening B factor (Å^2^) | -76.9 |
|  |  |
| Model Building and validation |  |
| Initial model used (PDB code) | 8Y0Q |
| MolProbity score | 1.56 |
| Clash score | 5.37 |
| Rotamer outliers (%) | 0.00 |
| R.M.S.D. of bond lengths (Å) | 0.004 |
| R.M.S.D. of angles (∘) | 0.600 |
| Favored (%) | 96.00 |
| Allowed (%) | 4.00 |
| Outliers (%) | 0.00 |

**Table B. Interface identification and interaction analysis of pO18-10 with FMDV O/18074.**

| **Interface** | | | | **Interaction** | | | |
| --- | --- | --- | --- | --- | --- | --- | --- |
| **O/18074** | **pO18-10** | **BSA(Å^2^)^a^** | **Percentage^b^** | **Type^c^** | **O/18074^d^** | **Dist.(Å)** | **pO18-10^e^** |
| **1: ILE194** | H chain | 38.17 | \|\|\|\| |  |  |  |  |
| **1: GLN195** | H chain | 3.68 | \| |  |  |  |  |
| **1: PRO196** | H chain | 9.89 | \|\| |  |  |  |  |
| **1: SER197** | H chain | 19.07 | \|\| | H  H | 1: SER197[N]  1: SER197[OG] | 3.08  3.76 | H: GLU106[OE2]  H: GLU106[OE1] |
| **1: THR198** | H chain | 20.84 | \|\|\| | H | 1: THR198[OG1] | 3.88 | H: GLU106[OE2] |
| **1: ARG200** | H chain | 0.74 | \| |  |  |  |  |
| **1: HIS201** | H chain | 38.81 | \|\|\|\|\| |  |  |  |  |
| **1: LYS202** | H chain | 58.89 | \|\|\|\|\| | H | 1: LYS202[N]  1: LYS202[O]  1: LYS202[O] | 3.50  2.96  3.46 | H: THR105[OG1]  H: THR105[N]  H: THR105[OG1] |
| **1: GLN203** | H chain | 21.18 | \|\| |  |  |  |  |
| **1: LYS204** | H chain | 141.94 | \|\|\|\|\|\|\| | H  H  H  S  S | 1: LYS204[NZ]  1: LYS204[NZ]  1: LYS204[NZ]  1: LYS204[NZ]  1: LYS204[NZ] | 2.90  2.85  3.01  2.90  2.85 | H: GLU34[OE1]  H: GLU34[OE2]  H: THR102[O]  H: GLU34[OE1]  H: GLU34[OE2] |
| **1: ILE205** | H chain | 3.67 | \| | H | 1: ILE205[O] | 3.75 | H: ARG32[NH2] |
| **1: PRO208** | H chain | 63.99 | \|\|\|\|\|\| |  |  |  |  |
| **1: VAL209** | H chain | 16.63 | \|\| |  |  |  |  |
| **1: LYS210** | H chain | 133.71 | \|\|\|\|\|\|\| | H  S  S | 1: LYS210[NZ]  1: LYS210[NZ]  1: LYS210[NZ] | 3.09  3.44  3.09 | H: ASP112[OD2]  H: ASP112[OD1]  H: ASP112[OD2] |
| **1: GLN211** | H chain | 74.46 | \|\|\|\| |  |  |  |  |
|  | H chain |  |  |  |  |  |  |
| **3: ASP173** | H chain | 19.86 | \|\|\| | H  H  S  S  S | 3: ASP173[OD1]  3: ASP173[OD2]  3: ASP173[OD1]  3: ASP173[OD1]  3: ASP173[OD2] | 3.41  3.35  3.79  3.41  3.35 | H: ARG32[NH2]  H: ARG32[NH1]  H: ARG32[NH1]  H: ARG32[NH2]  H: ARG32[NH1] |
| **3: VAL174** | H chain | 86.98 | \|\|\|\|\|\| |  |  |  |  |
| **3: ALA175** | H chain | 14.21 | \|\| |  |  |  |  |
| **3: THR177** | H chain | 29.12 | \|\|\| |  |  |  |  |
| **3: ARG218** | H chain | 29.55 | \|\| |  |  |  |  |
| **3: ALA219** | H chain | 7.15 | \| |  |  |  |  |
| **3: ARG218** | L chain | 11.45 | \| |  |  |  |  |
| **A: ILE48** | H chain | 62.57 | \| |  |  |  |  |
| **A: GLU95** | H chain | 127.67 | \|\|\|\|\| | H | A: GLU95[OE1] | 2.72 | H: TYR101[OH] |
| **A: ALA96** | H chain | 77.34 | \|\|\|\|\|\|\| |  |  |  |  |
| **A: ASP99** | H chain | 98.36 | \|\|\|\| | S | A: ASP99[OD2] | 3.21 | H: LYS110[NZ] |
| **A: ASN100** | H chain | 84.96 | \| |  |  |  |  |
| **A: LYS169** | H chain | 55.51 | \| |  |  |  |  |
| **A: GLU  46** | L chain | 19.48 | \|\|\| |  |  |  |  |
| **A: GLU47** | L chain | 23.45 | \|\|\|\| |  |  |  |  |
| **A: ASP99** | L chain | 36.92 | \|\|\|\| | S  S  S | A: ASP99[OD1]  A: ASP99[OD2]  A: ASP99[OD1] | 3.74  3.62  3.08 | L: ARG54[NH1]  L: ARG54[NH1]  L: ARG54[NH2] |
| **A: LYS169** | L chain | 16.61 | \|\|\| |  |  |  |  |
| **A: THR171** | L chain | 5.16 | \| |  |  |  |  |
| **A: ARG172** | L chain | 0.33 | \| |  |  |  |  |

a BSA: Buried Surface Area; b ||||: Buried area percentage, one bar per 10%. c H, Hydrogen bond; S, Salt bridge. d: Interactive atoms of amino acid side chain of O/18074. e: Interactive atoms of amino acid side chain of pO18-10.
