# Supplementary material for: Structural basis of a conserved, broad antigenic region surrounding the five-fold axis of foot-and-mouth disease virus revealed by swine neutralizing antibodies
Source: PLoS Pathog. 2026 Jul 14;22(7):e1014357. doi: 10.1371/journal.ppat.1014357 (PMC13367737; doi:10.1371/journal.ppat.1014357)
Supplement: Attachment — Submitted filename: Response to Reviewers.docx [file ppat.1014357.s004.docx]

Dear editors and reviewers:

Thank you for your letter and the reviewers' comments on our manuscript titled "Structural basis of a conserved, broad antigenic region surrounding the five-fold axis of foot-and-mouth disease virus revealed by swine neutralizing antibodies". The reviewers’ comments and suggestions have been extremely helpful and have substantially improved the clarity, rigor, and overall quality of our study.

We have carefully considered all comments and revised the manuscript accordingly. In addition, all figures have been thoroughly checked and adjusted using the NASEs tool to ensure compliance with journal requirements. The revised supplementary data and modified sections in the manuscript have been highlighted in yellow in the Supporting Information for ease of reference.

Detailed responses to each comment from the reviewers, together with a summary of the major revisions made to the manuscript, are provided below.

Best wishes

Kun Li

**Reviewers' Comments:**

Reviewer's Responses to Questions

**Part I - Summary**
Please use this section to discuss strengths/weaknesses of study, novelty/significance, general execution and scholarship.

Reviewer #1: This is an interesting study using porcine monoclonal antibodies to characterize broadly cross-reacting neutralizing antibodies. The revised manuscript has been significantly improved but some minor issues remain (see below).

Response:

We sincerely thank Reviewer #1 for the positive evaluation of our revised manuscript and for acknowledging the improvements made. We appreciate the remaining comments and have carefully addressed all minor issues, as detailed below.

Reviewer #2: No change
- The authors have addressed my questions.
The paper is now suitable for acceptance in my view.

Response:

We sincerely thank Reviewer #2 for the positive evaluation and are pleased that the manuscript is now considered suitable for acceptance.

Reviewer #3: The manuscript has been improved according to the comments indicated, and the points raised by this referee have also been addressed.
I recommend publication.

Response:

We sincerely thank Reviewer #3 for the positive feedback and recommendation for publication.

**Part II – Major Issues: Key Experiments Required for Acceptance**
Please use this section to detail the key new experiments or modifications of existing experiments that should be absolutely required to validate study conclusions.

Generally, there should be no more than 3 such required experiments or major modifications for a "Major Revision" recommendation. If more than 3 experiments are necessary to validate the study conclusions, then you are encouraged to recommend "Reject".

Reviewer #1: None

Reviewer #2: N/A

Reviewer #3: (No Response)

**Part III – Minor Issues: Editorial and Data Presentation Modifications**
Please use this section for editorial suggestions as well as relatively minor modifications of existing data that would enhance clarity.

Reviewer #1: Some minor issues that ought to be corrected.
1) In many places, including the title, the word “antigen” is used when I think it would be better as “antigenic”. See also lines 29,34, 38, 91, 96, 211 and possibly others, please check.
Response:

We thank the reviewer for this helpful suggestion. We have carefully checked the manuscript and revised “antigen” to “antigenic” where appropriate, including in the title and the indicated lines (29, 34, 38, 91, 96, and 211), as well as additional occurrences identified in lines 52, 83, 239, and 250. These revisions improve the accuracy and consistency of the terminology throughout the manuscript.

2) Line 49, I think “and Asia1” would be better
Response:

We thank the reviewer for this helpful suggestion. We have revised the text in Line 49 of the manuscript by changing it to “and Asia1”.

3) Line 82, change to “Distinct from….”
Response:

We thank the reviewer for the suggestion. We have revised the sentence in Line 82 accordingly to begin with “Distinct from…”.

4) Line 98, change to “can cross-react with…”
Response:

We thank the reviewer for the suggestion. We have revised the text in Line 98 accordingly by changing it to “can cross-react with…”.

5) Line 106 “the C-terminus” of what? I know it is VP1 but i think it should be stated.
Response:

We thank the reviewer for this helpful comment. We have revised the sentence in Line 106 to clarify that the “C-terminus” refers to the VP1 C-terminus.

6) In many places (e.g. line 145, 149, 161, 215, 224, 243, 244, 247, 346, 348, line 889 and probably elsewhere) amino acid changes or substitutions in proteins are referred to as mutations, this is incorrect. Mutations occur in nucleic acids. Please correct throughout.
Response:

We thank the reviewer for this important comment. We agree that “mutations” should be used to describe changes at the nucleic acid level rather than amino acid alterations in proteins. Accordingly, we have carefully revised the terminology throughout the manuscript, including all instances pointed out by the reviewer as well as other relevant sections, replacing “mutations” with more accurate terms such as “amino acid substitutions”, where appropriate, to ensure both accuracy and consistency in the manuscript.

7) Line 307, “secondly immunodominant” needs revising.
Response:

We thank the reviewer for the helpful suggestion. We have revised “secondly immunodominant” in Line 307 to “the second most immunodominant” for improved clarity and grammatical accuracy.

8) Table 1 title needs correction from “Procine” to “Porcine”.

Response:

We thank the reviewer for pointing out this typographical error. We have corrected “Procine” to “Porcine” in the title of Table 1.

Reviewer #2: N/A

Reviewer #3: (No Response)
