# Supplementary material for: Structural basis of a conserved, broad antigenic region surrounding the five-fold axis of foot-and-mouth disease virus revealed by swine neutralizing antibodies
Source: PLoS Pathog. 2026 Jul 14;22(7):e1014357. doi: 10.1371/journal.ppat.1014357 (PMC13367737; doi:10.1371/journal.ppat.1014357)
Supplement: Attachment — Submitted filename: Response to comments.docx [file ppat.1014357.s003.docx]

Dear editors and reviewers:

Thank you for your letter and the reviewers' comments on our manuscript titled "Structural basis of a conserved, broad antigen region surrounding the five-fold axis of foot-and-mouth disease virus revealed by swine neutralizing antibodies". These comments have been highly valuable and have substantially contributed to improving the clarity and rigor of our work, as well as providing important guidance for our research.

We have carefully considered all comments and have revised the manuscript accordingly. All figures have been thoroughly examined and adjusted using the PACE digital diagnostic tool, and the revised supplementary data have been highlighted in yellow within the Supporting Information. In response to the reviewers’ suggestions, we have further strengthened the manuscript by incorporating additional analyses and data.

Specifically, we sequenced viral genomes recovered from antibody-treated animals that ultimately succumbed to infection, assessed the stability of escape variants under antibody selection pressure, and provided local resolution analyses together with representative cryo-EM density maps of the epitope–paratope interface to support structural interpretation. In addition, we have revised and clarified the Data Availability statement to ensure transparency and accessibility. The main corrections in the paper along with our responses to editors' and reviewers' comments are outlined below.

Best wishes

Kun Li

**Journal Requirements:**

If the reviewer comments include a recommendation to cite specific previously published works, please review and evaluate these publications to determine whether they are relevant and should be cited. There is no requirement to cite these works unless the editor has indicated otherwise.

1) Please ensure that the CRediT author contributions listed for every co-author are completed accurately and in full.
At this stage, the following Authors/Authors require contributions: Qiongqiong Zhao, Shanquan Wu, Fengjuan Li, Lv Lv, Shulun Huang, Jiaxin Yang, Zhanhui Zhu, Ying Sun, Pinghua Li, Yimei Cao, Huifang Bao, Pu Sun, Xingwen Bai, Zhixun Zhao, Jing Zhang, Jinyan Wu, Hong Yuan, Jijun He, Xiangmin Li, Zengjun Lu, Dongsheng Lei, and Kun Li. Please ensure that the full contributions of each author are acknowledged in the "Add/Edit/Remove Authors" section of our submission form.
The list of CRediT author contributions may be found here: https://journals.plos.org/plospathogens/s/authorship#loc-author-contributions

Response:

Yes, we have checked all the authors and added their contributions.

2) We ask that a manuscript source file is provided at Revision. Please upload your manuscript file as a .doc, .docx, .rtf or .tex. If you are providing a .tex file, please upload it under the item type u2018LaTeX Source Fileu2019 and leave your .pdf version as the item type u2018Manuscriptu2019.

Response:
Yes. We confirm that the manuscript source file has been provided in .docx format at the time of revision.

3) Please provide an Author Summary. This should appear in your manuscript between the Abstract (if applicable) and the Introduction, and should be 150-200 words long. The aim should be to make your findings accessible to a wide audience that includes both scientists and non-scientists. Sample summaries can be found on our website under Submission Guidelines:
<https://journals.plos.org/plospathogens/s/submission-guidelines#loc-parts-of-a-submission>

Response:

Yes, we provided IMPORTANCE statement in our primary manuscript, which corresponds to the Author Summary. We made the change.

4) We have noticed that you have uploaded Supporting Information files, but you have not included a list of legends. Please add a full list of legends for your Supporting Information files after the references list.

Response:

Yes, we provided all the legends for the supporting information, which were named as “S1 Appendix.”.

5) Some material included in your submission may be copyrighted. According to PLOSu2019s copyright policy, authors who use figures or other material (e.g., graphics, clipart, maps) from another author or copyright holder must demonstrate or obtain permission to publish this material under the Creative Commons Attribution 4.0 International (CC BY 4.0) License used by PLOS journals. Please closely review the details of PLOSu2019s copyright requirements here: PLOS Licenses and Copyright. If you need to request permissions from a copyright holder, you may use PLOS's Copyright Content Permission form.
Please respond directly to this email and provide any known details concerning your material's license terms and permissions required for reuse, even if you have not yet obtained copyright permissions or are unsure of your material's copyright compatibility. Once you have responded and addressed all other outstanding technical requirements, you may resubmit your manuscript within Editorial Manager. 
Potential Copyright Issues:
i) Figure 1A. Please confirm whether you drew the images / clip-art within the figure panels by hand. If you did not draw the images, please provide (a) a link to the source of the images or icons and their license / terms of use; or (b) written permission from the copyright holder to publish the images or icons under our CC BY 4.0 license. Alternatively, you may replace the images with open source alternatives. See these open source resources you may use to replace images / clip-art:
- https://commons.wikimedia.org
- <https://openclipart.org/>.

Response:

We thank the Editorial Office for carefully reviewing the copyright status of the materials included in our submission. Regarding Figure 1A, the schematic elements were created using BioRender. The figure was generated in BioRender by Z. Zhao (2026) (<https://BioRender.com/7dik6qh>), and the corresponding attribution has been included in the figure legend.

6) In the online submission form, you indicated that  All other data supporting the findings of this study are available from the corresponding authors upon request.. All PLOS journals now require all data underlying the findings described in their manuscript to be freely available to other researchers, either
1. In a public repository
2. Within the manuscript itself
3. Uploaded as supplementary information.
This policy applies to all data except where public deposition would breach compliance with the protocol approved by your research ethics board. If your data cannot be made publicly available for ethical or legal reasons (e.g., public availability would compromise patient privacy), please explain your reasons by return email and your exemption request will be escalated to the editor for approval. Your exemption request will be handled independently and will not hold up the peer review process, but will need to be resolved should your manuscript be accepted for publication. One of the Editorial team will then be in touch if there are any issues.
Response:

Thank you for your message regarding the data availability policy. The antibody data supporting this study are subject to intellectual property restrictions under Patent No. ZL 2024 1 0612113.0. Consequently, these data are not deposited in a public repository. However, all underlying data will be freely available to researchers for non-commercial use upon request to the corresponding author. All other data supporting the findings of this study are within the paper and its [Supporting Information](https://journals.plos.org/plospathogens/article?id=10.1371/journal.ppat.1011132#sec036) files.

7) Thank you for stating 'The cryo-EM density map and 3D structure of the FMDV-O18-pO18-10 complex were deposited into the Electron Microscopy Data Bank (EMDB) and Protein Data Bank (PDB) with accession numbers EMD-66143 and 9WPH'. We found that this accession numbers directs to a 'dataset not found' page. Kindly fix it to point to an active link.

Response:

Thanks for the mention. We have made request of release and PDB data will be available one week later.

**Reviewers' Comments:**

Reviewer's Responses to Questions

**Part I - Summary**
Please use this section to discuss strengths/weaknesses of study, novelty/significance, general execution and scholarship.

Reviewer #1: The manuscript by Qiongqiong Zhao et al., describes a comprehensive study on the characterisation of neutralising monoclonal antibodies generated in pigs that are able to neutralise foot-and-mouth disease viruses (FMDVs) from different serotypes (O, A and Asia1. Normally there is little or no cross protection between different serotypes of FMDV. The authors identify key residues in the FMDVs which affect binding of the antibodies. In many ways the antibodies resemble other murine antibodies that neutralise FMDV and it has been possible to generate neutralisation-resistant mutants of the viruses with single amino acid substitutions. The authors have determined the interactions between the viruses and antibodies using cryo-EM, it is indicated that the antibodies likely hinder binding of the receptor to the virus. It is also shown that treatment of mice with these antibodies protects against FMDV infection.

Response:

Yes, thanks for the comments.

Reviewer #2: I recommend accept with major revisions.

The paper describes the properties of an interesting and potentially valuable reagent. The monoclonal antibody identified cross-reacts with a number of FMDV serotypes, in which respect it is very unusual.
I believe the experiments are all carried out to an acceptable standard and broadly justify the main conclusions drawn.

The reason why I ask for major revisions is not that I am requesting further experiments, I think it is a well rounded study, nevertheless as it stands the paper has some notable shortcomings, some being listed below. If these are addressed I believe the paper warrants publication in PLOS Pathogens.

Response:

Thanks very much for the advices. These comments are valuable and contributed great for improvement of the manuscript.

Reviewer #3: The manuscript by Zhao et al describes the production and characterization of four porcine-derived monoclonal antibodies that broadly neutralize multiple representative strains from FMDV serotypes O and A (bnAbs). These bnAbs recognize a conserved antigen region with a key determinant residue 204 (K) at the VP1 C-terminus. On of them, bnAb pO18-10, which competed with antibodies recognizing the G-H loop, C-terminus on VP1 and epitopes on VP3, and exhibited the broadest neutralization breadth, including cross-neutralization against a serotype Asia 1 strain.
Cryo-electron microscopy structure of pO18-10 in complex with virions (146S) from FMDV serotype O revealed a novel cross-protomer antigen region located around the icosahedral five-fold axis of the viral capsid, involving interactions with the C-terminus of VP1 and the G-H loop of VP3 from one protomer, as well as the E-F loop of VP1 from an adjacent protomer.
The VP1 residues 204 (K) and 210 (K) were identified as key determinants of this cross-serotype antigen site. Furthermore, evidence is provided supporting that pO18-10 neutralizes FMDV by directly blocking viral attachment. In vivo, pO18-10 provided robust prophylactic and therapeutic protection against FMDV-induced mortality and clinical disease in mice.
The authors conclude that these bnAbs are candidates for immunodiagnostic application and the cross-serotype antigenic structure for universal FMDV vaccine design.

This is an interesting, comprehensive and, in my opinion, well conducted work. The topic is also relevant: the identification and characterization of FMDV antigenic sites capable to elicit antibodies that neutralize isolates from different serotypes.
Nevertheless, there are conceptual aspects and issues regarding data and manuscript presentation that have to be addressed before the manuscript can be considered for publication.

Response:

Yes, thanks for the comments.

**Part II – Major Issues: Key Experiments Required for Acceptance**
Please use this section to detail the key new experiments or modifications of existing experiments that should be absolutely required to validate study conclusions.

Generally, there should be no more than 3 such required experiments or major modifications for a "Major Revision" recommendation. If more than 3 experiments are necessary to validate the study conclusions, then you are encouraged to recommend "Reject".

Reviewer #1:

1) In Tables 1 and 2, the amino acid substitutions that confer resistance are listed, however, it would be good if the codon changes were also indicated so that it was easy for the reader to tell if single or multiple nt changes were required to bring about the change. Note, the virus was passaged in cells in the presence of the antibody so there was the opportunity for multiple changes to occur.

Response:

Yes, thank you for this valuable suggestion. We have now included the codon changes in Tables 1 and 2, allowing readers to clearly see whether each amino acid substitution arises from single or multiple nucleotide changes.

2) The selection of neutralisation resistant mutants was performed in BHK cells. It is rather easy for FMDV to adapt to the use of heparan sulfate (HS), as a receptor in BHK cells, this is commonly associated with changes at residues 56 or 58 in VP3. Such a change was not observed but maybe the viruses used were already adapted to use HS. The authors should comment on this. It is important for the suggestion that the neutralisation was achieved through blocking of the interaction with the integrin receptors.

Response:

Yes, we agree with this comment. Accordingly, we have added the following discussion to the revised manuscript (Lines 372-380): “It is well established that FMDV can readily adapt to use heparan sulfate (HS) as a receptor during passage in BHK cells, a process frequently associated with amino acid substitutions at residues 56 (H56R) or 58 (K56E) of VP3 (Lee G. Viruses. 2020; Subramaniam S. Virus Genes. 2017). In our study, however, sequencing of the neutralisation-resistant mutants showed that residues 56 and 58 in VP3 remained unchanged. One possible explanation is that the parental virus used in this study had already adapted to BHK cells and acquired the ability to utilize HS as a receptor prior to the selection experiment. Furthermore, structural superposition between FMDV-integrin and FMDV-antibody complexes revealed notable obvious clashes between pO18-10 and the integrin receptor, suggesting that the neutralisation was achieved through blocking of the interaction with the integrin receptors.”

3) In the mice that survived for longer with the virus in the presence of the antibody (see Figure 6 A), did the virus that eventually killed the mice have any sequence changes from the challenge virus? Are such viruses now resistant to neutralisation by the administered antibody?

Response:

Yes, we thank the reviewer for this insightful comment, as it can highlight the differences in viral mutation characteristics and mutation rates under antibody pressure in vivo versus in vitro. To determine whether the viruses responsible for lethality in suckling mice that survived longer in the presence of the antibody had acquired sequence changes relative to the original challenge virus, we performed additional experiments and sequencing analyses. In parallel prophylactic and therapeutic experiments, a total of 7 viral isolates were obtained from mice treated with PBS as controls, and 18 lethal viral isolates were obtained from mice infected in the presence of the pO18-10 antibody. Sequencing analysis showed that most of isolates remained genetically stable in vivo, including 5/7 isolates in the control group and 8/18 isolates in prophylactic and therapeutic groups. The mutated isolates showed synonymous mutaions at positions VP2-384 nt, VP4-15 nt, VP4-195 nt, VP2-196 nt, VP2-384 nt, VP2-618 nt, VP3-243 nt, and VP1-444 nt. Only seven isolates were observed with nonsynonymous substitutions, showing residues at positions 4 (A→T/V), 194 (I→T) and 197(S→R) on VP1, as well as residue 60(D→G) on VP3. The detail results were shown as the below figure.

As for “Are such viruses now resistant to neutralisation by the administered antibody?”. Unluckily, we did not try to recover the variants from the lethal mice. However, given to many nonsynonymous mutations outside the antigenic region, the variants could not escape pO18-10-mediated neutralization.

**Fig legend: Sequence analysis of viral strain variations in neonatal suckling KM mice infected in the presence of pO18-10.** Nucleotide and amino acid variations in viral sequences associated with mortality in suckling KM mice are shown for the prophylactic **(A)** and therapeutic **(B)** groups. The P1 region of each virus was amplified by one-step reverse transcription-PCR (RT-PCR) using primers Pan204+ (ACCTCCAACGGGTGGTACGC) and NK61 (GACATGTCCTCTTGCATCTG) and verified by sequencing. Mutated residues were determined by alignment with sequences from the PBS control group. In the panels, red boxes indicate mutated residues, and green boxes indicate synonymous mutations.

Reviewer #2: The major issues to be addressed are:  

1) presentation. The paper must be throughly checked and the english corrected throughout. This could be classed as a minor issue, however the reality is that there are significant ambiguities introduced which could mislead the reader. There are far too many issues to detail them all. But for example the final paragraph of introduction contains at least six significant grammatical errors. Perhaps the manuscript was assembled in a hurry. 
Example of misleading text: Line 147 “204 (K) at the VP1 C-terminus,” - residue 204 is not at the C-terminus, is is towards the C_terminus. This occurs at least twice (eg it is referred to as ‘C-terminal’ on line 156 - this is even more misleading). 
Response:

Thank you for your thorough review and constructive feedback. Regarding the grammatical errors throughout the manuscript, we have conducted a comprehensive review and correction of the text.

2) Structural analysis. On line 164 the resolution is quoted at 2.27 Å. This is fine as far as it goes but in reality it is useless - what is the resolution of the regions of interest?  There is nowhere any indication of the resolution in different regions, a local resolution map is essential in such an analysis, and there should be pictures of the density in the epitope/paratope regions. Since the antibody sits quite 'high' and not in close contact with the bulk of the capsid this is essential evidence. I would suspect that the density for in these regions is at much lower resolution. What scraps of evidence are presented - an overview of density - seems to fit with this concern, although it is impossible to tell. I am happy to believe the broad messages of the paper, but it is impossible to know if any of the structural results are justified by the data. For instance in native FMDV the C-terminus of VP1 is generally not well ordered.... is it re-ordered here? In addition there is no discussion of whether antibody binding leads to a change in conformation of any of the regions defining the epitope, I suspect there are significant changes at the VP1 C-terminus at least, but it is impossible to say.
Response:

We thank the reviewer for raising this important point regarding the interpretation of the cryo-EM map. We agree that the global resolution alone is insufficient to access the local map quality, particularly at the epitope-paratope interface. To address this, we have added a new figure (Fig C panels A in S1 Text) presenting a local resolution analysis, which shows that resolution across the reconstruction ranges from ~2.0 Å to ~3.2 Å, including at the interaction interface. This result supports reliable modeling in these regions. We now also provide representative cryo-EM density, fitted with atomic models, for the O18-pO18-10 protomer, the VP1 C-terminal region, and the antibody-virus interface (Fig C panels B-D in S1 Text). Although the antibody binds in an elevated position relative to the capsid, the density at the interface is well-defined, allowing unambiguous assignment of the contacts (Fig C panels C and E in S1 Text). Furthermore, in contrast to the previously reported O18 structure (PDB: 8Y0Q) where this region is poorly ordered, the VP1 C-terminus in our complex displays clear density, enabling its modeling (Fig C panels D and E in S1 Text). A direct comparison of the two structures shows a clear difference in the conformation of the VP1 C-terminus (Fig C panels E in S1 Text). Together, these additional analyses strengthen the structural conclusions presented in the manuscript. We added the following comment in the manuscript (lines 174-182).

3) Line 300: I find this section very worrying. Alphafold is not an experimental method. the authors have determined the structure of the antibody complex (and have built a model based on this for the epitope and paratope) - there is no sign of an interaction with the VP1 GH loop in that, so it is simply not acceptable to use a method such as alphafold, which is very poor at predicting Ab/Ag complexes (especially novel ones), as an argument that there is a direct interaction. In contrast, steric interference is a perfectly reasonable argument. So the alpha fold stuff should be removed.

Response:

We thank the reviewer for this helpful suggestion. We agree with the reviewer’s concern regarding the use of AlphaFold predictions to infer antibody-antigen interactions. Accordingly, we have removed the AlphaFold-based analyses and the corresponding discussion from the revised manuscript, as well as Fig J panels B and C in S1 Text.

Reviewer #3:

1) The connection of experiments detailed in this manuscript with those parented in their previous article (PLoS Pathogens; <https://doi.org/10.1371/journal.ppat.1012623> ), should be clearly stated and critically discussed by the authors. Are the chronotypes used in this manuscript those shown in the previous article (Fig. 1) in which the analyses were focused in the common chronotypes? If so, which was the reason for focusing the search of widely broad neutralizing antibodies on type O-specific chronotypes rather than in common chronotypes?

Response:

We thank the reviewer for this insightful comment. In our previous study (PLoS Pathogens; <https://doi.org/10.1371/journal.ppat.1012623>), we focused on common chronotypes to investigate conserved antigenic structures between O and A serotypes. Specifically, we screened 20 IgG antibodies from 216 shared clones derived from the O/18074- and A/AF72-specific B cell libraries and identified 12 broadly neutralizing antibodies (bnAbs) across both serotypes using virus neutralization assays. In the present study, our objective shifted to examining antigenic variation among classical and circulating O serotype Cathay topotype strains. To this end, we screened 57 IgG antibodies from 2014 clones within the O/18074-specific B cell library. This analysis unexpectedly yielded four bnAbs targeting the VP1-C terminus, including pO18-10, which also neutralizes Asia1 viruses. Importantly, the emphasis on type O-specific chronotypes was not driven by a targeted search for bnAbs; rather, these antibodies were identified serendipitously. We have now clarified this point and explicitly discussed its implications in the revised manuscript (lines 320-326): “Although previous analyses focused on common chronotypes (PLoS Pathogens; <https://doi.org/10.1371/journal.ppat.1012623>), the incidental identification of bnAbs from type O-specific chronotypes suggests that restricting analyses to common clonotypes may underestimate the breadth of neutralizing responses. The cross-serotype activity of pO18-10 further indicates that rare, serotype-restricted clones can recognize structurally conserved epitopes. Together, these findings highlight a potential limitation of current screening strategies and suggest that integrating both common and serotype-specific chronotypes may be necessary to fully define the landscape of protective antibody responses.”

2) The rationale behind considering as remarkable the cross protection afforded by mA pO18-10 against the Asia1 JS 05 isolate, should be discussed. According to the limited sequence alignment in Fig.4, that of Asia1/JS/05 is not clearly more diverse than those of the other type O isolates compared. This alignment should incorporate additional Asia 1 sequences to understand the extent of the cross protection claimed, as well as include additional Asia 1 isolates to support the conclusion.

Response:

Yes, we thank the reviewer for this point. Asia1 was withdrawn from China’s vaccination programme in 2018 and has not been detected in clinical surveillance since then; moreover, work with live Asia1 virus in China requires approval from the Ministry of Agriculture and Rural Affairs and our reference laboratory currently holds only the Asia1/JS/05 isolate, so regulatory and material constraints prevented experimental testing against additional Asia1 strains. To address the reviewer’s concern, we have expanded the sequence analysis by including all available Asia1 VP1 antigenic‑region sequences from GenBank (Fig. 4D). This broader alignment shows that VP1 residue 99, while not strictly conserved in Asia 1, tends toward Asp (D) similar to type O viruses; VP1 residue 210 is conserved as Lys (K) across Asia1 and is consistent with O and A types; and VP3 residue 173, although variable, also tends toward Asp (D) as seen in O and A strains. Because mAb pO18‑10 recognizes epitopes that include these positions, the observed similarity at VP1‑210 and the shared tendencies at VP1‑99 and VP3‑173 provide a plausible molecular basis for cross‑neutralization of multiple Asia 1 strains by pO18‑10.

Therefore, we added the description (lines 235-236) in main text as follows, “For Asia1, VP1-99 shows a bias toward Asp (D), VP1-210 is conserved as Lys (K), and VP3-173, although variable, also tends toward Asp (D), consistent with patterns observed in types O and A.”

3) The stability upon growth passages of the mutations selected in the neutralization-scape mutants (Table 1), as well as that of the site-directed mutants should be tested to confirm the residue changes shown in Tables 1 and 2, and Fig.4.

Response:

We thank the reviewer for this valuable suggestion. To evaluate the genetic stability of the mutations identified in the neutralization-escape mutants (Tables 1 and 2), we performed three additional serial passages for 19 mutants and re-sequenced the corresponding regions. Among these, seven mutants (7/19) remained genetically stable during passaging, with no changes detected at the originally identified mutation sites. In contrast, twelve mutants (12/19) exhibited alterations at the mutated positions during viral propagation. Of these, ten mutants ultimately showed reversion mutations at the mutated sites during passaging. These reversions occurred at VP3-173 in pO18-10-O/18074-mutant-A; VP3-174 in pO18-10-O/18074-mutant-B; VP1-99 and VP1-143 in pO18-10-O/18074-mutant-E; VP3-174 in pO18-10-O/18074-mutant-F; VP1-99 in pO18-17-O/18074-mutant-A; VP2-68 and VP3-173 in pO18-52-O/HN/CHA/93-mutant-A; VP3-173 in pO18-53-O/HN/CHA/93-mutant-F; VP1-204 in pO18-53-O/HN/CHA/93-mutant-H; and VP3-210 in pO18-10-A/WH/CHA/09-mutant-D. In addition, a synonymous mutation (VP1-T561C) was detected in pO18-17-O/18074-mutant-C at passage 2, which reverted to the original nucleotide by passage 3. These results indicate that a subset of escape mutations is genetically stable, whereas others show a tendency to revert during viral replication. The corresponding results have now been incorporated into the revised manuscript (Fig B panel A-E in S1 Text) and described in the main text (lines 157-167). In addition, for all site-directed mutants shown in Fig. 4, the rescued viruses were further passaged for five generations following successful virus rescue, after which the relevant regions were re-sequenced. The single-residue substitutions remained genetically stable during propagation, with no evidence of reversion to the wild-type sequence or the emergence of additional mutations.

**Part III – Minor Issues: Editorial and Data Presentation Modifications**
Please use this section for editorial suggestions as well as relatively minor modifications of existing data that would enhance clarity.

Reviewer #1:

a) To treat pigs or cattle with monoclonal antibodies would presumably be very expensive, is it realistic to think this approach could replace the use of vaccines to combat FMD? The authors should comment.
Response:

We thank the reviewer for this important comment. We agree that the large-scale use of monoclonal antibodies (mAbs) in livestock would currently be cost-prohibitive, making them unlikely to replace vaccination as the primary strategy for FMD control. Vaccination remains the most practical and cost-effective approach for population-level protection. However, accumulating evidence indicates that mAbs can serve as valuable complementary tools. Passive antibody administration has been shown to confer rapid, short-term protection, which may be particularly useful in emergency outbreak settings, for high-value animals, or as a bridge before vaccine-induced immunity develops. In addition, advances in antibody engineering are improving production efficiency and in vivo stability, enhancing their practical potential. We therefore view mAbs not as a replacement for vaccines, but as a targeted adjunct strategy under specific scenarios. We added the following comment in the manuscript (lines 362-365): “While mAbs are unlikely to replace vaccination, they could provide rapid, short-term protection in outbreaks, serve as a bridge until vaccine-induced immunity develops, or protect high-value animals, highlighting their potential as a complementary tool in FMD control.”

b) Although the text is understandable throughout there are many minor errors in the use of English.

Response:

We thank the reviewer for this observation. We have carefully revised the manuscript to correct minor grammatical and stylistic errors and have ensured that the text is now polished and consistent, improving clarity and readability throughout.

Reviewer #2:

a) LIne 168: It is completely misleading to to quote the BSA to 2 decimal places - this is a measure that is very sensitive to coordinate error and we have no idea how reliable the model is. And so I would be very cautious to say this is the biggest contact area seen so far.
Response:

Thanks for the comments and we modified the modified the description. “total surface area of 1452.35 Å²” were corrected as “total surface area of approximately 1452 Å²”. Additionally, “These antigenic footprints show more extensive coverage than any other serotype-specific or cross-serotype antibody-FMDV complexes currently available in the PDB.”

b) Line 171 onwards: “The interaction is predominantly mediated by the VH domain (92.2%), with a smaller contribution from VL (7.8%), as shown by PISA (Fig 3A and Supplemental Table 2). The antibody VH and VL mediate equal contact area with complex FMDV-O18-pO18-10, and make 9 hydrogen bonds and 9 salt bridges at the interface (Table 3).” - These two sentences are contradictory, I suspect the first is correct.
Response:

Thank you for pointing out this inconsistency. Upon verification, the first sentence is correct, whereas the second sentence contained contradictory information, which has now been revised accordingly. The corrected text is as follows: “In the FMDV-O18–pO18-10 complex, the VH domain accounts for the majority of the buried surface area at the interface, forming 9 hydrogen bonds and 8 salt bridges, whereas the VL domain contributes only 3 salt bridges and plays a minor role in the interaction (Table 3).”

c) Line 216: “VP1204 (K) and VP1210 (D)” - Earlier VP1 residue 210 is defined a lysine. (also in many places the difference in residues numbers between serotypes in the C-terminal region of VP1 makes the text confusing).

Response:

Thank you for pointing this out. We have corrected Line 216 by changing “_VP1_210 (D)” to “_VP1_210 (K)”. Compared with the O/18074 strain, the A/WH/CHA/09 strain contains deletions at residues 140 and 141 and an insertion at residue 197 in the VP1 protein. Consequently, _VP1_203 and _VP1_209 of the A/WH/CHA/09 strain correspond to _VP1_204 and _VP1_210 of the O/18074 strain, respectively. This has now been clarified in lines 219-221.

Reviewer #3:

a) Line 143. The reason for using different type O viruses (Table 1, parent virus) for mAb scape mutants should be clarified.
Response:

We thank the reviewer for this comment. The O/HN/CHA/93 strain is highly stable during passaging and is therefore routinely used for the selection of escape mutants under antibody pressure. However, as shown in Fig. 1B, pO18-10 and pO18-17 exhibit poor neutralizing activity against O/HN/CHA/93. To increase the likelihood of successfully isolating escape mutants for these two antibodies, we instead performed escape mutant selection using the O/18074 strain, against which they display stronger neutralizing activity.

b) The quality of the downloaded Figures is poor, particularly that of Fig.4, in which reading the X-axis and Y-axis title, and understanding the meaning of the logos in section D are very difficult.
Response:

Thank you for your careful evaluation. We have regenerated all figures at higher resolution and improved their overall clarity, including axis labels and annotations. For Fig. 4D, this panel presents an alignment of viral capsid protein sequences available in GenBank (NCBI, as of June 30th, 2025). VP1 is predominantly 213 amino acids in serotypes O and A, and 211 amino acids in serotype Asia1; VP3 lengths are 220, 221, and 219 amino acids for serotypes O, A, and Asia1, respectively. The figure has been redrawn accordingly to accurately reflect these features. A corresponding description has been added to the revised manuscript (lines 229-234).

c) Line 149. Check the meaning of the sentence: “The VP1 C-terminal 204 (K) was observed in the four bnAbs-escaping mutants, suggesting …… “
Response:

Thank you for your careful review. We have revised the sentence to clarify its meaning. The updated version now reads: “All four bnAbs-escaping mutants carried a mutation at residue 204 (K) towards the VP1 C-terminus, highlighting its critical role in antibody-mediated neutralization.”

d) Line 150, replace antibodies by mAbs
Response:

We thank the reviewer for this helpful suggestion. To ensure consistency throughout the manuscript, we have replaced “antibodies” with “bnAbs” in the revised version.

e) Please, indicate the procedure for production of serotype A site-directed mutatants (Fig.4).
Were the mutations introduced in the site-directed FMDV mutants (Fig. 4) confirmed by nucleotide sequencing of the whole P1 region?
Response:

Thank you for this valuable comment. Serotype A site-directed mutants were generated using an infectious clone–based reverse genetics system. Briefly, mutations encoding the desired amino acid substitutions were introduced by PCR-mediated site-directed mutagenesis into the full-length cDNA clone, followed by cloning into the corresponding plasmid backbone. Recombinant viruses were rescued by transfection of linearized plasmids into BSR/T7 cells and subsequent passaging in BHK-21 cells. A detailed description of these procedures has been added to the Methods section of the revised manuscript. Yes, all site-directed FMDV mutants were verified by nucleotide sequencing of the entire P1-coding region, confirming the presence of the intended mutations and the absence of unintended substitutions.

f) Check the consistency of the sentence “Alignment of amino acid sequences …… (lines 211-215).
Response:

We thank the reviewer for the suggestion. We have revised the sentence for clarity and consistency. The updated sentence now reads: “Alignment of viral capsid protein sequences available in GenBank (NCBI, as of June 30^th^, 2025) revealed that _VP1_204 (K) is conserved in both serotypes O and A, whereas _VP1_210 (K) and _VP3_173 (D) are highly conserved across serotypes O, A, and Asia1.”

g) Line 219. VP1 position 95 does not seem to significantly reduce virus neutralization titer in Fig.4E.
Response:

We thank the reviewer for this careful observation. Neutralization differences between the wild type and its mutants were analyzed using an unpaired t-test with the Holm–Sidak method (α = 0.05) in GraphPad Prism (v9.5.1). The results indicate that the mutation at VP1 position 95 shows a statistically significant difference compared with the wild type (P = 0.0470), as indicated by a single asterisk in Fig. 4E (P < 0.05).

h) The origin of the mAbs other than those reported here shown in Fig. S3, should be indicated in both the figure legend and the Discussion section.
Response:

We thank the reviewer for this helpful suggestion. We have now clarified the origin of the mAbs shown in Fig E in S1 Text (previously Fig. S3) in both the figure legend and the Discussion section. Specifically, we have added the following text to lines 302-305 of the revised manuscript: “Specifically, bnAbs targeting cross-site 1 and cross-site 2 were derived from 216 clonotype antibodies shared between the porcine O/18074- and A/AF72-specific B cell libraries; the cross-site 3-related bnAb R50 was isolated from a natural bovine host using single B cell antibody isolation; and bnAbs targeting cross-site 4 were identified in this study from the porcine O/18074-specific B cell library.”

i) Comment to lines 340 to 343: prophylactic peptide vaccines targeted to VP1 GH loop in combination to T-cell peptides can confer solid experimental protection in swine.
Response:

We thank the reviewer for this insightful comment. We modified the statement and added the references (lines 366-372). “Evidence suggests that prophylactic peptide vaccines targeting the VP1 G-H loop in combination with T-cell peptides may confer effective experimental protection in swine (Wang CY. Vaccine. 2002; Cao N. ACS Nano. 2025). Furthermore, while linear epitopes of the G-H loop provide a foundation, the inclusion of complex conformational epitopes is likely critical for eliciting high-titer neutralizing antibodies. Given that single-epitope strategies often prove insufficient in livestock, future vaccine design should focus on multi-component platforms that combine T-cell stimulation with structurally authentic B-cell epitopes to ensure a more comprehensive and durable immune response.”

j) The use of the abbreviations mAb, MaAb and bnAbs, as well as the term “antibodies” should be harmonized along the text.
As this is a competitive topic, the authors should extensively review potential related publications.

Response:

We thank the reviewer for this helpful suggestion. We have revised the manuscript to ensure consistent terminology throughout. Specifically, antibodies that have not yet been evaluated in neutralization assays are now uniformly referred to as monoclonal antibodies (mAbs), whereas those demonstrated to broadly neutralize different serotypes of FMDV are consistently designated as broadly neutralizing antibodies (bnAbs).
