# Supplementary material for: Structural basis of a conserved, broad antigenic region surrounding the five-fold axis of foot-and-mouth disease virus revealed by swine neutralizing antibodies
Source: PLoS Pathog. 2026 Jul 14;22(7):e1014357. doi: 10.1371/journal.ppat.1014357 (PMC13367737; doi:10.1371/journal.ppat.1014357)
Supplement: Peer review history

# Peer review: Decision Letter 0

**DOI:** 10.1371/journal.ppat.1014357.r001

**Authors:** George Belov, Ashley St. John

19 Jan 2026

-->PPATHOGENS-D-25-02382

Structural basis of a conserved, broad antigen region surrounding the five-fold axis of foot-and-mouth disease virus revealed by swine neutralizing antibodies

PLOS Pathogens

Dear Dr. Li,

Thank you for submitting your manuscript to PLOS Pathogens. After careful consideration, we feel that it has merit but does not fully meet PLOS Pathogens's publication criteria as it currently stands. Therefore, we invite you to submit a revised version of the manuscript that addresses the points raised during the review process.

The reviewers appreciated the discovery and characterization of  broadly neutralizing anti-FMDV antibodies, but also indicated important shortcomings of how the data are presented and interpreted.

Please submit your revised manuscript by Mar 20 2026 11:59PM. If you will need more time than this to complete your revisions, please reply to this message or contact the journal office at plospathogens@plos.org. When you're ready to submit your revision, log on to https://www.editorialmanager.com/ppathogens/ and select the 'Submissions Needing Revision' folder to locate your manuscript file.

Please include the following items when submitting your revised manuscript:

* A letter that responds to each point raised by the editor and reviewer(s). You should upload this letter as a separate file labeled 'Response to Reviewers'. This file does not need to include responses to any formatting updates and technical items listed in the 'Journal Requirements' section below.

* A marked-up copy of your manuscript that highlights changes made to the original version. You should upload this as a separate file labeled 'Revised Manuscript with Track Changes'.

* An unmarked version of your revised paper without tracked changes. You should upload this as a separate file labeled 'Manuscript'.

If you would like to make changes to your financial disclosure, competing interests statement, or data availability statement, please make these updates within the submission form at the time of resubmission. Guidelines for resubmitting your figure files are available below the reviewer comments at the end of this letter.

We look forward to receiving your revised manuscript.

Kind regards,

George A. Belov, PhD

Academic Editor

PLOS Pathogens

Ashley St. John

Section Editor

PLOS Pathogens

Sumita Bhaduri-McIntosh

Editor-in-Chief

PLOS Pathogens

orcid.org/0000-0003-2946-9497

Michael Malim

-->Editor-in-Chief

PLOS Pathogens

orcid.org/0000-0002-7699-2064

**Journal Requirements:**

If the reviewer comments include a recommendation to cite specific previously published works, please review and evaluate these publications to determine whether they are relevant and should be cited. There is no requirement to cite these works unless the editor has indicated otherwise.

1) Please ensure that the CRediT author contributions listed for every co-author are completed accurately and in full.

At this stage, the following Authors/Authors require contributions: Qiongqiong Zhao, Shanquan Wu, Fengjuan Li, Lv Lv, Shulun Huang, Jiaxin Yang, Zhanhui Zhu, Ying Sun, Pinghua Li, Yimei Cao, Huifang Bao, Pu Sun, Xingwen Bai, Zhixun Zhao, Jing Zhang, Jinyan Wu, Hong Yuan, Jijun He, Xiangmin Li, Zengjun Lu, Dongsheng Lei, and Kun Li. Please ensure that the full contributions of each author are acknowledged in the "Add/Edit/Remove Authors" section of our submission form.

The list of CRediT author contributions may be found here: https://journals.plos.org/plospathogens/s/authorship#loc-author-contributions

2) We ask that a manuscript source file is provided at Revision. Please upload your manuscript file as a .doc, .docx, .rtf or .tex. If you are providing a .tex file, please upload it under the item type u2018LaTeX Source Fileu2019 and leave your .pdf version as the item type u2018Manuscriptu2019.

3) Please provide an Author Summary. This should appear in your manuscript between the Abstract (if applicable) and the Introduction, and should be 150-200 words long. The aim should be to make your findings accessible to a wide audience that includes both scientists and non-scientists. Sample summaries can be found on our website under Submission Guidelines:

https://journals.plos.org/plospathogens/s/submission-guidelines#loc-parts-of-a-submission

4) We have noticed that you have uploaded Supporting Information files, but you have not included a list of legends. Please add a full list of legends for your Supporting Information files after the references list.

5) Some material included in your submission may be copyrighted. According to PLOSu2019s copyright policy, authors who use figures or other material (e.g., graphics, clipart, maps) from another author or copyright holder must demonstrate or obtain permission to publish this material under the Creative Commons Attribution 4.0 International (CC BY 4.0) License used by PLOS journals. Please closely review the details of PLOSu2019s copyright requirements here: PLOS Licenses and Copyright. If you need to request permissions from a copyright holder, you may use PLOS's Copyright Content Permission form.

Please respond directly to this email and provide any known details concerning your material's license terms and permissions required for reuse, even if you have not yet obtained copyright permissions or are unsure of your material's copyright compatibility. Once you have responded and addressed all other outstanding technical requirements, you may resubmit your manuscript within Editorial Manager.

Potential Copyright Issues:

i) Figure 1A. Please confirm whether you drew the images / clip-art within the figure panels by hand. If you did not draw the images, please provide (a) a link to the source of the images or icons and their license / terms of use; or (b) written permission from the copyright holder to publish the images or icons under our CC BY 4.0 license. Alternatively, you may replace the images with open source alternatives. See these open source resources you may use to replace images / clip-art:

- https://commons.wikimedia.org

- https://openclipart.org/.

6) In the online submission form, you indicated that  All other data supporting the findings of this study are available from the corresponding authors upon request.. All PLOS journals now require all data underlying the findings described in their manuscript to be freely available to other researchers, either

1. In a public repository

2. Within the manuscript itself

3. Uploaded as supplementary information.

This policy applies to all data except where public deposition would breach compliance with the protocol approved by your research ethics board. If your data cannot be made publicly available for ethical or legal reasons (e.g., public availability would compromise patient privacy), please explain your reasons by return email and your exemption request will be escalated to the editor for approval. Your exemption request will be handled independently and will not hold up the peer review process, but will need to be resolved should your manuscript be accepted for publication. One of the Editorial team will then be in touch if there are any issues.

7) Thank you for stating 'The cryo-EM density map and 3D structure of the FMDV-O18-pO18-10 complex were deposited into the Electron Microscopy Data Bank (EMDB) and Protein Data Bank (PDB) with accession numbers EMD-66143 and 9WPH'. We found that this accession numbers directs to a 'dataset not found' page. Kindly fix it to point to an active link.

**Reviewers' Comments:**

Reviewer's Responses to Questions

**Part I - Summary**

Please use this section to discuss strengths/weaknesses of study, novelty/significance, general execution and scholarship.

Reviewer #1: The manuscript by Qiongqiong Zhao et al., describes a comprehensive study on the characterisation of neutralising monoclonal antibodies generated in pigs that are able to neutralise foot-and-mouth disease viruses (FMDVs) from different serotypes (O, A and Asia1. Normally there is little or no cross protection between different serotypes of FMDV. The authors identify key residues in the FMDVs which affect binding of the antibodies. In many ways the antibodies resemble other murine antibodies that neutralise FMDV and it has been possible to generate neutralisation-resistant mutants of the viruses with single amino acid substitutions. The authors have determined the interactions between the viruses and antibodies using cryo-EM, it is indicated that the antibodies likely hinder binding of the receptor to the virus. It is also shown that treatment of mice with these antibodies protects against FMDV infection.

Reviewer #2: I recommend accept with major revisions.

The paper describes the properties of an interesting and potentially valuable reagent. The monoclonal antibody identified cross-reacts with a number of FMDV serotypes, in which respect it is very unusual.

I believe the experiments are all carried out to an acceptable standard and broadly justify the main conclusions drawn.

The reason why I ask for major revisions is not that I am requesting further experiments, I think it is a well rounded study, nevertheless as it stands the paper has some notable shortcomings, some being listed below. If these are addressed I believe the paper warrants publication in PLOS Pathogens.

Reviewer #3: The manuscript by Zhao et al describes the production and characterization of four porcine-derived monoclonal antibodies that broadly neutralize multiple representative strains from FMDV serotypes O and A (bnAbs). These bnAbs recognize a conserved antigen region with a key determinant residue 204 (K) at the VP1 C-terminus. On of them, bnAb pO18-10, which competed with antibodies recognizing the G-H loop, C-terminus on VP1 and epitopes on VP3, and exhibited the broadest neutralization breadth, including cross-neutralization against a serotype Asia 1 strain.

Cryo-electron microscopy structure of pO18-10 in complex with virions (146S) from FMDV serotype O revealed a novel cross-protomer antigen region located around the icosahedral five-fold axis of the viral capsid, involving interactions with the C-terminus of VP1 and the G-H loop of VP3 from one protomer, as well as the E-F loop of VP1 from an adjacent protomer.

The VP1 residues 204 (K) and 210 (K) were identified as key determinants of this cross-serotype antigen site. Furthermore, evidence is provided supporting that pO18-10 neutralizes FMDV by directly blocking viral attachment. In vivo, pO18-10 provided robust prophylactic and therapeutic protection against FMDV-induced mortality and clinical disease in mice.

The authors conclude that these bnAbs are candidates for immunodiagnostic application and the cross-serotype antigenic structure for universal FMDV vaccine design.

This is an interesting, comprehensive and, in my opinion, well conducted work. The topic is also relevant: the identification and characterization of FMDV antigenic sites capable to elicit antibodies that neutralize isolates from different serotypes.

Nevertheless, there are conceptual aspects and issues regarding data and manuscript presentation that have to be addressed before the manuscript can be considered for publication.

**********

**Part II – Major Issues: Key Experiments Required for Acceptance**

Please use this section to detail the key new experiments or modifications of existing experiments that should be absolutely required to validate study conclusions.

Generally, there should be no more than 3 such required experiments or major modifications for a "Major Revision" recommendation. If more than 3 experiments are necessary to validate the study conclusions, then you are encouraged to recommend "Reject".

Reviewer #1: 1) In Tables 1 and 2, the amino acid substitutions that confer resistance are listed, however, it would be good if the codon changes were also indicated so that it was easy for the reader to tell if single or multiple nt changes were required to bring about the change. Note, the virus was passaged in cells in the presence of the antibody so there was the opportunity for multiple changes to occur.

2) The selection of neutralisation resistant mutants was performed in BHK cells. It is rather easy for FMDV to adapt to the use of heparan sulfate (HS), as a receptor in BHK cells, this is commonly associated with changes at residues 56 or 58 in VP3. Such a change was not observed but maybe the viruses used were already adapted to use HS. The authors should comment on this. It is important for the suggestion that the neutralisation was achieved through blocking of the interaction with the integrin receptors.

3) In the mice that survived for longer with the virus in the presence of the antibody (see Figure 6 A), did the virus that eventually killed the mice have any sequence changes from the challenge virus? Are such viruses now resistant to neutralisation by the administered antibody?

Reviewer #2: The major issues to be addressed are:

1) presentation. The paper must be throughly checked and the english corrected throughout. This could be classed as a minor issue, however the reality is that there are significant ambiguities introduced which could mislead the reader.There are far too many issues to detail them all. But for example the final paragraph of introduction contains at least six significant grammatical errors. Perhaps the manuscript was assembled in a hurry.

Example of misleading text: Line 147 “204 (K) at the VP1 C-terminus,” - residue 204 is not at the C-terminus, is is towards the C_terminus. This occurs at least twice (eg it is referred to as ‘C-terminal’ on line 156 - this is even more misleading).

2. Structural analysis. On line 164 the resolution is quoted at 2.27 Å. This is fine as far as it goes but in reality it is useless - what is the resolution of the regions of interest? There is nowhere any indication of the resolution in different regions, a local resolution map is essential in such an analysis, and there should be pictures of the density in the epitope/paratope regions. Since the antibody sits quite 'high' and not in close contact with the bulk of the capsid this is essential evidence. I would suspect that the density for in these regions is at much lower resolution. What scraps of evidence are presented - an overview of density - seems to fit with this concern, although it is impossible to tell. I am happy to believe the broad messages of the paper, but it is impossible to know if any of the structural results are justified by the data. For instance in native FMDV the C-terminus of VP1 is generally not well ordered.... is it re-ordered here? In addition there is no discussion of whether antibody binding leads to a change in conformation of any of the regions defining the epitope, I suspect there are significant changes at the VP1 C-terminus at least, but it is impossible to say.

3. Line 300: I find this section very worrying. Alphafold is not an experimental method. the authors have determined the structure of the antibody complex (and have built a model based on this for the epitope and paratope) - there is no sign of an interaction with the VP1 GH loop in that, so it is simply not acceptable to use a method such as alphafold, which is very poor at predicting Ab/Ag complexes (especially novel ones), as an argument that there is a direct interaction. In contrast, steric interference is a perfectly reasonable argument. So the alpha fold stuff should be removed.

Reviewer #3: The connection of experiments detailed in this manuscript with those parented in their previous article (PLoS Pathogens; https://doi.org/10.1371/journal.ppat.1012623 ), should be clearly stated and critically discussed by the authors. Are the chronotypes used in this manuscript those shown in the previous article (Fig. 1) in which the analyses were focused in the common chronotypes? If so, which was the reason for focusing the search of widely broad neutralizing antibodies on type O-specific chronotypes rather than in common chronotypes?

The rationale behind considering as remarkable the cross protection afforded by mA pO18-10 against the Asia1 JS 05 isolate, should be discussed. According to the limited sequence alignment in Fig.4, that of Asia1/JS/05 is not clearly more diverse than those of the other type O isolates compared. This alignment should incorporate additional Asia 1 sequences to understand the extent of the cross protection claimed, as well as include additional Asia 1 isolates to support the conclusion.

The stability upon growth passages of the mutations selected in the neutralization-scape mutants (Table 1), as well as that of the site-directed mutants should be tested to confirm the residue changes shown in Tables 1 and 2, and Fig.4.

**********

**Part III – Minor Issues: Editorial and Data Presentation Modifications**

Please use this section for editorial suggestions as well as relatively minor modifications of existing data that would enhance clarity.

Reviewer #1: a) To treat pigs or cattle with monoclonal antibodies would presumably be very expensive, is it realistic to think this approach could replace the use of vaccines to combat FMD? The authors should comment.

b) Although the text is understandable throughout there are many minor errors in the use of English.

Reviewer #2: LIne 168: It is completely misleading to to quote the BSA to 2 decimal places - this is a measure that is very sensitive to coordinate error and we have no idea how reliable the model is. And so I would be very cautious to say this is the biggest contact area seen so far.

Line 171 onwards:“The interaction is predominantly mediated by the VH domain (92.2%), with a smaller contribution from VL (7.8%), as shown by PISA (Fig 3A and Supplemental Table 2). The antibody VH and VL mediate equal contact area with complex FMDV-O18-pO18-10, and make 9 hydrogen bonds and 9 salt bridges at the interface (Table 3).”- These two sentences are contradictory, I suspect the first is correct.

Line 216: “VP1204 (K) and VP1210 (D)” - Earlier VP1 residue 210 is defined a lysine.(also in many places the difference in residues numbers between serotypes in the C-terminal region of VP1 makes the text confusing).

Reviewer #3: Line 143. The reason for using different type O viruses (Table 1, parent virus) for mAb scape mutants should be clarified.

The quality of the downloaded Figures is poor, particularly that of Fig.4, in which reading the X-axis and Y-axis title, and understanding the meaning of the logos in section D are very difficult.

Line 149. Check the meaning of the sentence: “The VP1 C-terminal 204 (K) was observed in the four bnAbs-escaping mutants, suggesting …… “

Line 150, replace antibodies by mAbs

Please, indicate the procedure for production of serotype A site-directed mutatants (Fig.4).

Were the mutations introduced in the site-directed FMDV mutants (Fig. 4) confirmed by nucleotide sequencing of the whole P1 region?

Check the consistency of the sentence “Alignment of amino acid sequences …… (lines 211-215).

Line 219. VP1 position 95 does not seem to significantly reduce virus neutralization titer in Fig.4E.

The origin of the mAbs other than those reported here shown in Fig. S3, should be indicated in both the figure legend and the Discussion section.

Comment to lines 340 to 343: prophylactic peptide vaccines targeted to VP1 GH loop in combination to T-cell peptides can confer solid experimental protection in swine.

The use of the abbreviations mAb, MaAb and bnAbs, as well as the term “antibodies” should be harmonized along the text.

As this is a competitive topic, the authors should extensively review potential related publications.

**********

PLOS authors have the option to publish the peer review history of their article (what does this mean?). If published, this will include your full peer review and any attached files.

If you choose “no”, your identity will remain anonymous but your review may still be made public.

**Do you want your identity to be public for this peer review?**  For information about this choice, including consent withdrawal, please see our Privacy Policy.

Reviewer #1: No

Reviewer #2: No

Reviewer #3: No

**Figure resubmission:**

-->While revising your submission, we strongly recommend that you use PLOS’s NAAS tool (https://ngplosjournals.pagemajik.ai/artanalysis) to test your figure files. NAAS can convert your figure files to the TIFF file type and meet basic requirements (such as print size, resolution), or provide you with a report on issues that do not meet our requirements and that NAAS cannot fix.

After uploading your figures to PLOS’s NAAS tool - https://ngplosjournals.pagemajik.ai/artanalysis, NAAS will process the files provided and display the results in the "Uploaded Files" section of the page as the processing is complete. If the uploaded figures meet our requirements (or NAAS is able to fix the files to meet our requirements), the figure will be marked as "fixed" above. If NAAS is unable to fix the files, a red "failed" label will appear above. When NAAS has confirmed that the figure files meet our requirements, please download the file via the download option, and include these NAAS processed figure files when submitting your revised manuscript.

**Reproducibility:**

To enhance the reproducibility of your results, we recommend that authors of applicable studies deposit laboratory protocols in protocols.io, where a protocol can be assigned its own identifier (DOI) such that it can be cited independently in the future. Additionally, PLOS ONE offers an option to publish peer-reviewed clinical study protocols. Read more information on sharing protocols at https://plos.org/protocols?utm_medium=editorial-email&utm_source=authorletters&utm_campaign=protocols

# Author comment: Author response to Decision Letter 1

**DOI:** 10.1371/journal.ppat.1014357.r002

20 Apr 2026

Attachment Submitted filename: Response to comments.docx

# Peer review: Decision Letter 1

**DOI:** 10.1371/journal.ppat.1014357.r003

**Authors:** George Belov, Ashley St. John, George Belov, Ashley St. John

26 May 2026

PPATHOGENS-D-25-02382R1

Structural basis of a conserved, broad antigen region surrounding the five-fold axis of foot-and-mouth disease virus revealed by swine neutralizing antibodies

PLOS Pathogens

Dear Dr. Li,

Thank you for submitting your manuscript to PLOS Pathogens. After careful consideration, we feel that it has merit but does not fully meet PLOS Pathogens's publication criteria as it currently stands. Therefore, we invite you to submit a revised version of the manuscript that addresses the points raised during the review process.

Please submit your revised manuscript by Jul 25 2026 11:59PM. If you will need more time than this to complete your revisions, please reply to this message or contact the journal office at plospathogens@plos.org. When you're ready to submit your revision, log on to https://www.editorialmanager.com/ppathogens/ and select the 'Submissions Needing Revision' folder to locate your manuscript file.

Please include the following items when submitting your revised manuscript:

* A letter that responds to each point raised by the editor and reviewer(s). You should upload this letter as a separate file labeled 'Response to Reviewers'. This file does not need to include responses to any formatting updates and technical items listed in the 'Journal Requirements' section below.

* A marked-up copy of your manuscript that highlights changes made to the original version. You should upload this as a separate file labeled 'Revised Manuscript with Track Changes'.

* An unmarked version of your revised paper without tracked changes. You should upload this as a separate file labeled 'Manuscript'.

If you would like to make changes to your financial disclosure, competing interests statement, or data availability statement, please make these updates within the submission form at the time of resubmission. Guidelines for resubmitting your figure files are available below the reviewer comments at the end of this letter.

As the corresponding author, your ORCID iD is verified in the submission system and will appear in the published article. PLOS supports the use of ORCID, and we encourage all coauthors to register for an ORCID iD and use it as well. Please encourage your coauthors to verify their ORCID iD within the submission system before final acceptance, as unverified ORCID iDs will not appear in the published article. Only the individual author can complete the verification step; PLOS staff cannot verify ORCID iDs on behalf of authors.

We look forward to receiving your revised manuscript.

Kind regards,

George A. Belov, PhD

Academic Editor

PLOS Pathogens

Ashley St. John

Section Editor

PLOS Pathogens

Sumita Bhaduri-McIntosh

Editor-in-Chief

PLOS Pathogens

orcid.org/0000-0003-2946-9497

Michael Malim

Editor-in-Chief

PLOS Pathogens

orcid.org/0000-0002-7699-2064

If the reviewer comments include a recommendation to cite specific previously published works, please review and evaluate these publications to determine whether they are relevant and should be cited. There is no requirement to cite these works unless the editor has indicated otherwise.

**Reviewers' Comments:**

Reviewer's Responses to Questions

**Part I - Summary**

Please use this section to discuss strengths/weaknesses of study, novelty/significance, general execution and scholarship.

Reviewer #1: This is an interesting study using porcine monoclonal antibodies to characterize broadly cross-reacting neutralizing antibodies. The revised manuscript has been significantly improved but some minor issues remain (see below).

Reviewer #2: No change

- The authors have addressed my questions.

The paper is now suitable for acceptance in my view.

Reviewer #3: The manuscript has been improved according to the comments indicated, and the points raised by this referee have also been addressed.

I recommend publication.

**********

**Part II – Major Issues: Key Experiments Required for Acceptance**

Please use this section to detail the key new experiments or modifications of existing experiments that should be absolutely required to validate study conclusions.

Generally, there should be no more than 3 such required experiments or major modifications for a "Major Revision" recommendation. If more than 3 experiments are necessary to validate the study conclusions, then you are encouraged to recommend "Reject".

Reviewer #1: None

Reviewer #2: N/A

Reviewer #3: (No Response)

**********

**Part III – Minor Issues: Editorial and Data Presentation Modifications**

Please use this section for editorial suggestions as well as relatively minor modifications of existing data that would enhance clarity.

Reviewer #1: Some minor issues that ought to be corrected.

1) In many places, including the title, the word “antigen” is used when I think it would be better as “antigenic”. See also lines 29,34, 38, 91, 96, 211 and possibly others, please check.

2) Line 49, I think “and Asia1” would be better

3) Line 82, change to “Distinct from….”

4) Line 98, change to “can cross-react with…”

5) Line 106 “the C-terminus” of what? I know it is VP1 but i think it should be stated.

6) In many places (e.g. line 145, 149, 161, 215, 224, 243, 244, 247, 346, 348, line 889 and probably elsewhere) amino acid changes or substitutions in proteins are referred to as mutations, this is incorrect. Mutations occur in nucleic acids. Please correct throughout.

7) Line 307, “secondly immunodominant” needs revising.

8) Table 1 title needs correction from “Procine” to “Porcine”.

Reviewer #2: N/A

Reviewer #3: (No Response)

**********

PLOS authors have the option to publish the peer review history of their article (what does this mean?). If published, this will include your full peer review and any attached files.

If you choose “no”, your identity will remain anonymous but your review may still be made public.

**Do you want your identity to be public for this peer review?**  For information about this choice, including consent withdrawal, please see our Privacy Policy.

Reviewer #1: No

Reviewer #2: No

Reviewer #3: **Yes:**  Francisco Sobrino

[NOTE: If reviewer comments were submitted as an attachment file, they will be attached to this email and accessible via the submission site. Please log into your account, locate the manuscript record, and check for the action link "View Attachments". If this link does not appear, there are no attachment files.]

**Figure resubmission:**

-->While revising your submission, we strongly recommend that you use PLOS’s NAAS tool (https://ngplosjournals.pagemajik.ai/artanalysis) to test your figure files. NAAS can convert your figure files to the TIFF file type and meet basic requirements (such as print size, resolution), or provide you with a report on issues that do not meet our requirements and that NAAS cannot fix.

After uploading your figures to PLOS’s NAAS tool - https://ngplosjournals.pagemajik.ai/artanalysis, NAAS will process the files provided and display the results in the "Uploaded Files" section of the page as the processing is complete. If the uploaded figures meet our requirements (or NAAS is able to fix the files to meet our requirements), the figure will be marked as "fixed" above. If NAAS is unable to fix the files, a red "failed" label will appear above. When NAAS has confirmed that the figure files meet our requirements, please download the file via the download option, and include these NAAS processed figure files when submitting your revised manuscript.

**Reproducibility:**

To enhance the reproducibility of your results, we recommend that authors of applicable studies deposit laboratory protocols in protocols.io, where a protocol can be assigned its own identifier (DOI) such that it can be cited independently in the future. Additionally, PLOS ONE offers an option to publish peer-reviewed clinical study protocols. Read more information on sharing protocols at https://plos.org/protocols?utm_medium=editorial-email&utm_source=authorletters&utm_campaign=protocols

# Author comment: Author response to Decision Letter 2

**DOI:** 10.1371/journal.ppat.1014357.r004

30 May 2026

Attachment Submitted filename: Response to Reviewers.docx

# Editor report: Decision Letter 2

**DOI:** 10.1371/journal.ppat.1014357.r005

**Authors:** George Belov, Ashley St. John, George Belov, Ashley St. John, George Belov, Ashley St. John

7 Jun 2026

Dear Dr Li,

We are pleased to inform you that your manuscript 'Structural basis of a conserved, broad antigenic region surrounding the five-fold axis of foot-and-mouth disease virus revealed by swine neutralizing antibodies' has been provisionally accepted for publication in PLOS Pathogens.

Before your manuscript can be formally accepted you will need to complete some formatting changes, which you will receive in a follow up email. A member of our team will be in touch with a set of requests.

Please note that your manuscript will not be scheduled for publication until you have made the required changes, so a swift response is appreciated.

IMPORTANT: The editorial review process is now complete. PLOS will only permit corrections to spelling, formatting or significant scientific errors from this point onwards. Requests for major changes, or any which affect the scientific understanding of your work, will cause delays to the publication date of your manuscript.

Should you, your institution's press office or the journal office choose to press release your paper, you will automatically be opted out of early publication. We ask that you notify us now if you or your institution is planning to press release the article. All press must be co-ordinated with PLOS.

Thank you again for supporting Open Access publishing; we are looking forward to publishing your work in PLOS Pathogens.

Best regards,

George A. Belov, PhD

Academic Editor

PLOS Pathogens

Ashley St. John

Section Editor

PLOS Pathogens

Sumita Bhaduri-McIntosh

Editor-in-Chief

PLOS Pathogens

orcid.org/0000-0003-2946-9497

Michael Malim

Editor-in-Chief

PLOS Pathogens

orcid.org/0000-0002-7699-2064

***********************************************************

Reviewer Comments (if any, and for reference):

# Editor report: Acceptance letter

**DOI:** 10.1371/journal.ppat.1014357.r006

**Authors:** George Belov, Ashley St. John, George Belov, Ashley St. John, George Belov, Ashley St. John

Dear Dr Li,

We are delighted to inform you that your manuscript, "Structural basis of a conserved, broad antigenic region surrounding the five-fold axis of foot-and-mouth disease virus revealed by swine neutralizing antibodies," has been formally accepted for publication in PLOS Pathogens.

We have now passed your article onto the PLOS Production Department who will complete the rest of the pre-publication process. All authors will receive a confirmation email upon publication.

The corresponding author will soon be receiving a typeset proof for review, to ensure errors have not been introduced during production. Please review the PDF proof of your manuscript carefully, as this is the last chance to correct any scientific or type-setting errors. Please note that major changes, or those which affect the scientific understanding of the work, will likely cause delays to the publication date of your manuscript. Note: Proofs for Front Matter articles (Pearls, Reviews, Opinions, etc...) are generated on a different schedule and may not be made available as quickly.

Soon after your final files are uploaded, the early version of your manuscript, if you opted to have an early version of your article, will be published online. The date of the early version will be your article's publication date. The final article will be published to the same URL, and all versions of the paper will be accessible to readers.

For Research Articles, you will receive an invoice from PLOS for your publication fee after your manuscript has reached the completed accept phase. If you receive an email requesting payment before acceptance or for any other service, this may be a phishing scheme. Learn how to identify phishing emails and protect your accounts at https://explore.plos.org/phishing.

Thank you again for supporting open-access publishing; we are looking forward to publishing your work in PLOS Pathogens.

Best regards,

Sumita Bhaduri-McIntosh

Editor-in-Chief

PLOS Pathogens

orcid.org/0000-0003-2946-9497

Michael Malim

Editor-in-Chief

PLOS Pathogens

orcid.org/0000-0002-7699-2064
